# Supplementary figures and images for: Signal denoising through topographic modularity of neural circuits
Source: eLife. 2023 Jan 26;12:e77009. doi: 10.7554/eLife.77009 (PMC9981157; doi:10.7554/eLife.77009)

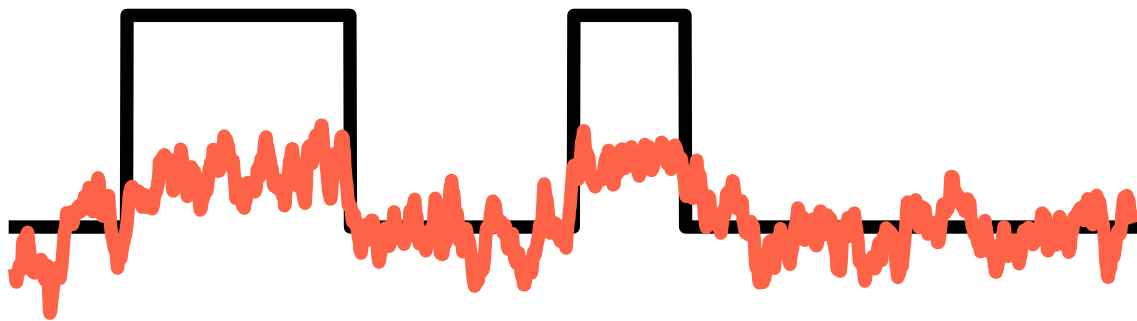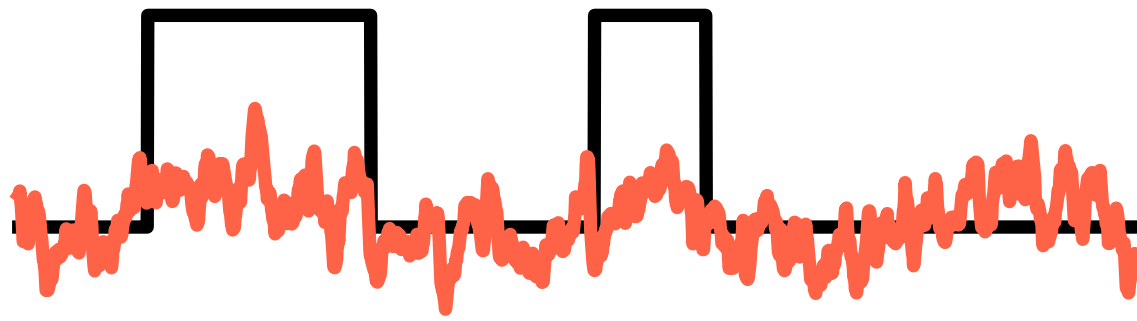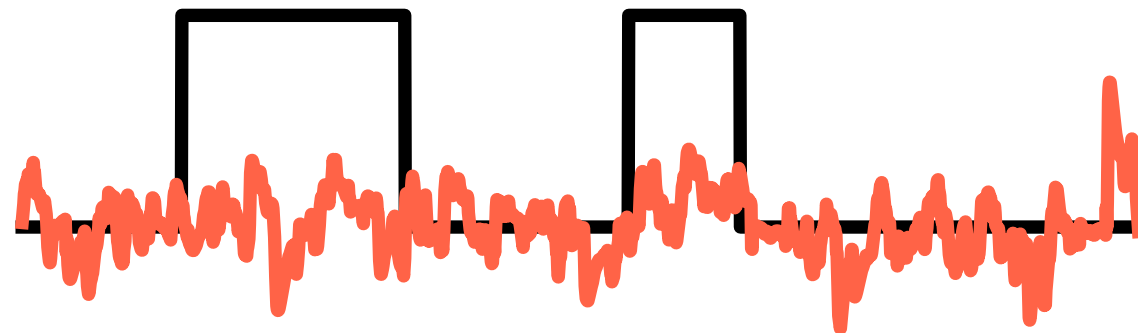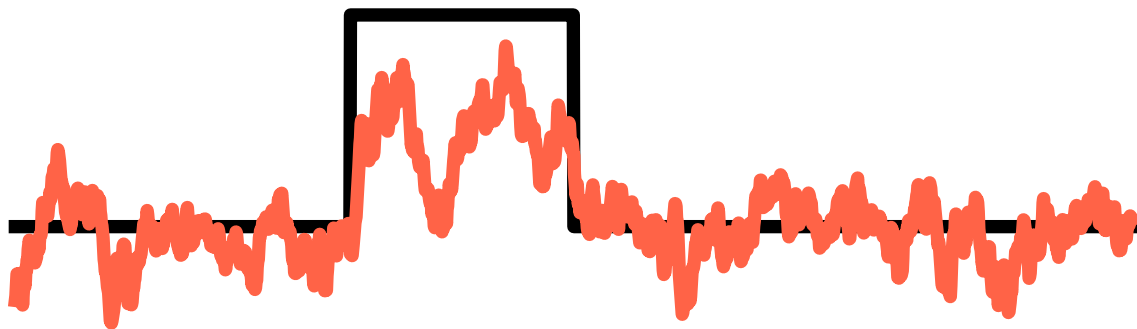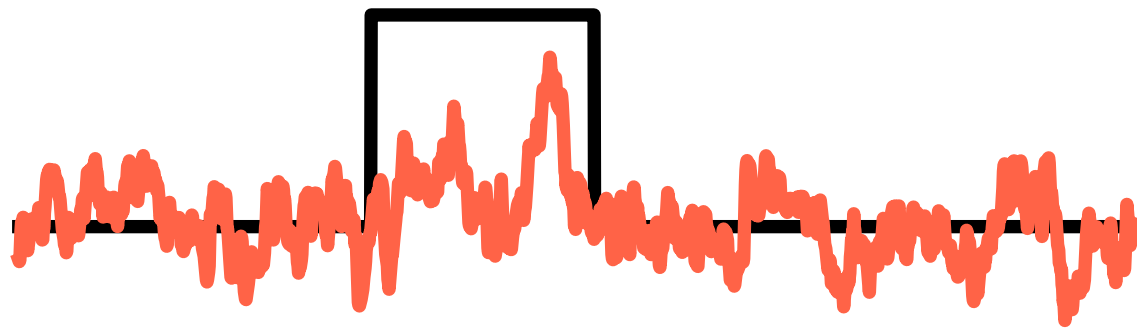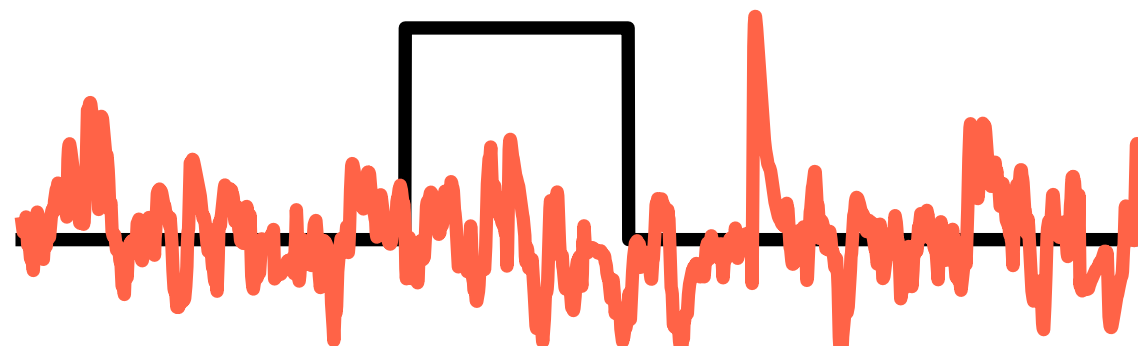

Supplement: Figure 1—source data 1. [file elife-77009-fig1-data1.zip › figure1/plots/fig1_b.pdf]

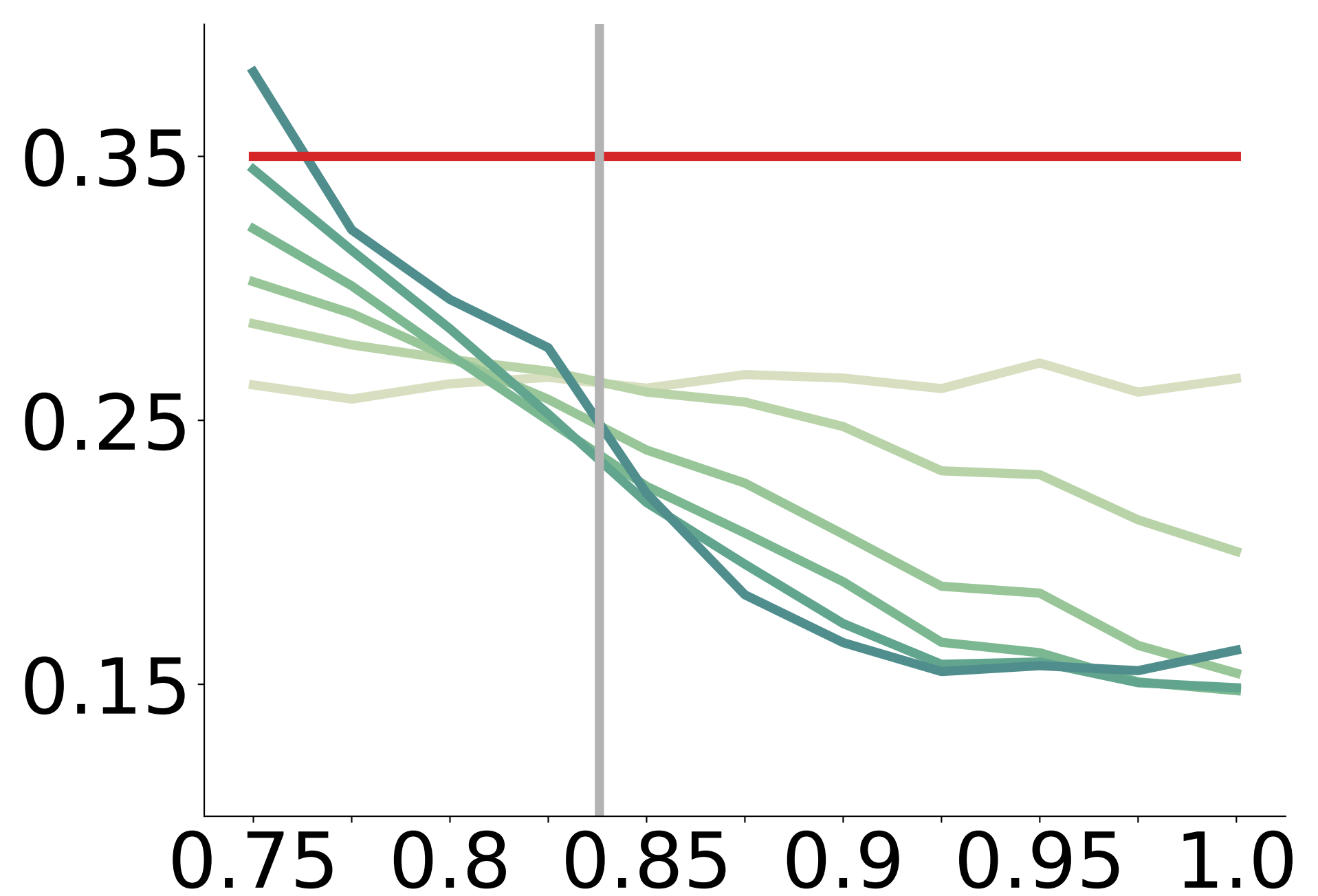

Supplement: Figure 1—source data 1. [file elife-77009-fig1-data1.zip › figure1/plots/fig1_c.pdf]

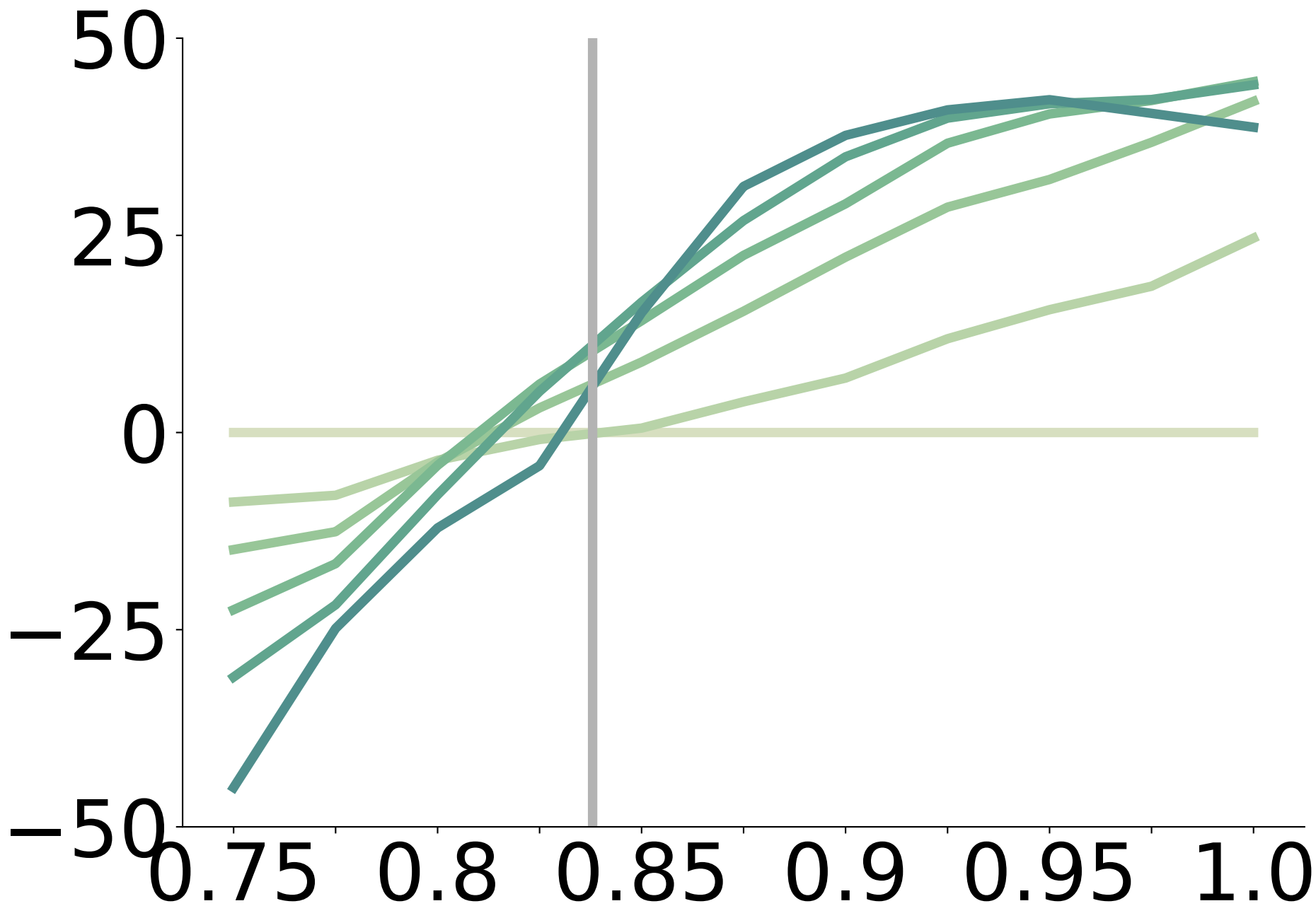

Supplement: Figure 1—source data 1. [file elife-77009-fig1-data1.zip › figure1/plots/fig1_d.pdf]

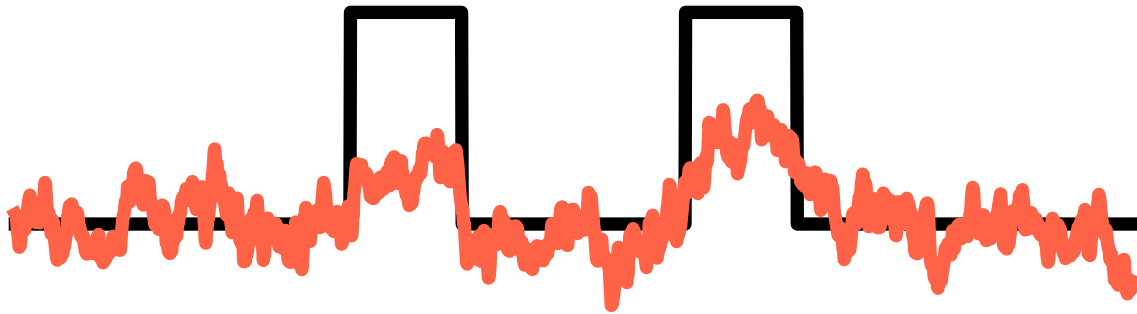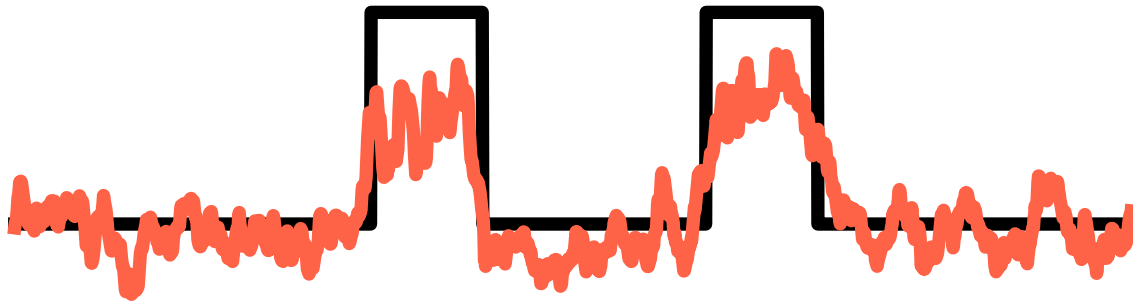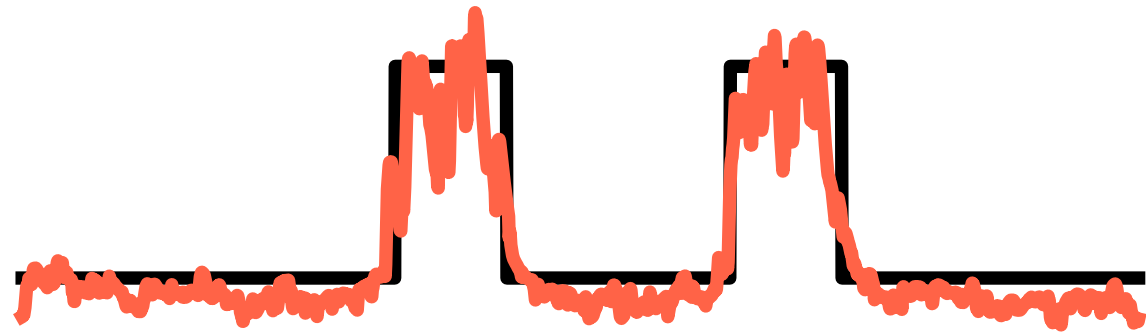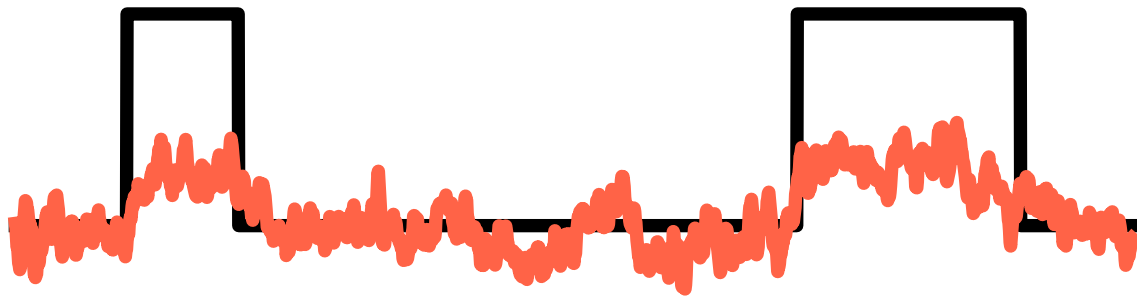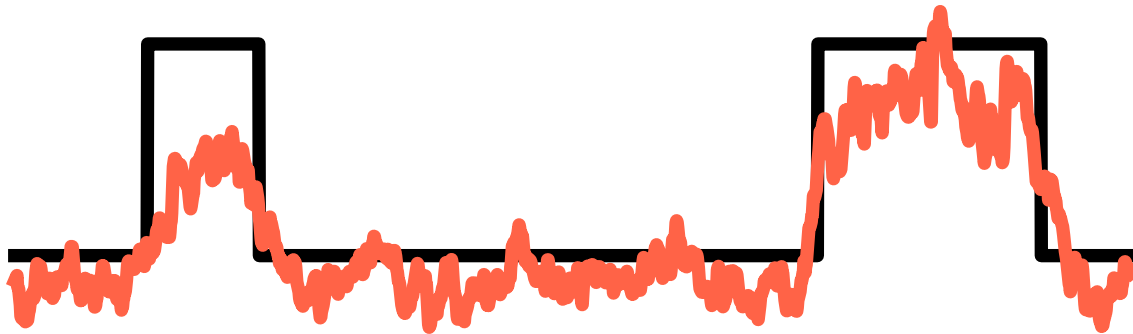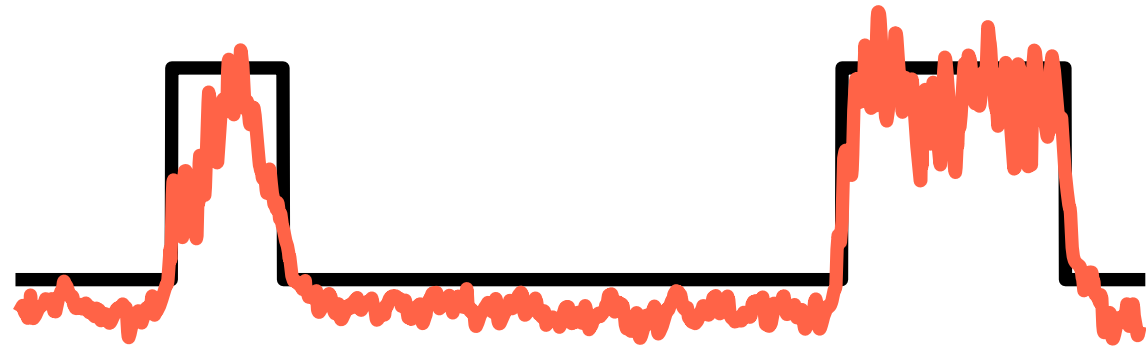

Supplement: Figure 1—source data 1. [file elife-77009-fig1-data1.zip › figure1/plots/fig1_e.pdf]

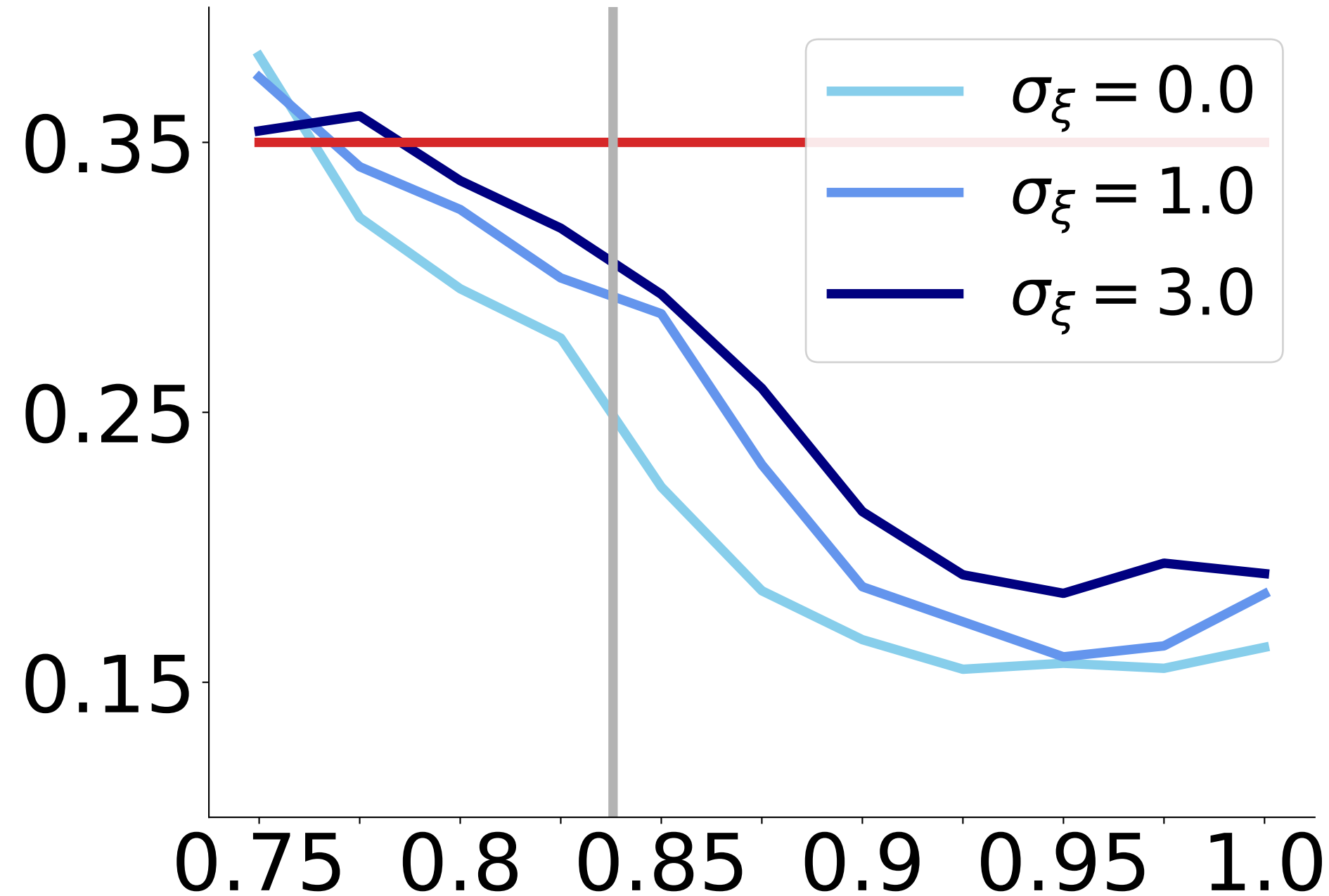

Supplement: Figure 1—source data 1. [file elife-77009-fig1-data1.zip › figure1/plots/fig1_f.pdf]

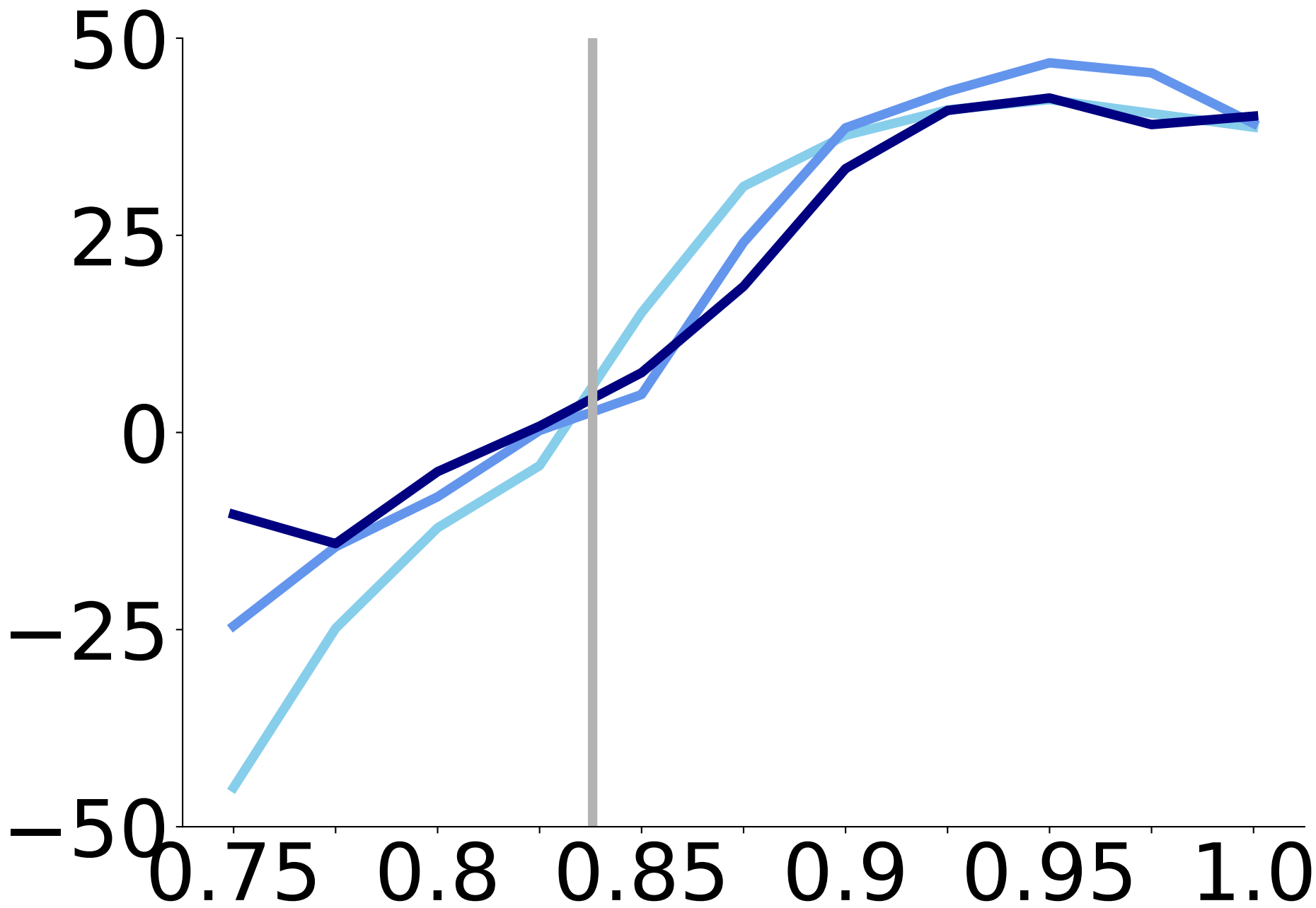

Supplement: Figure 1—source data 1. [file elife-77009-fig1-data1.zip › figure1/plots/fig1_g.pdf]

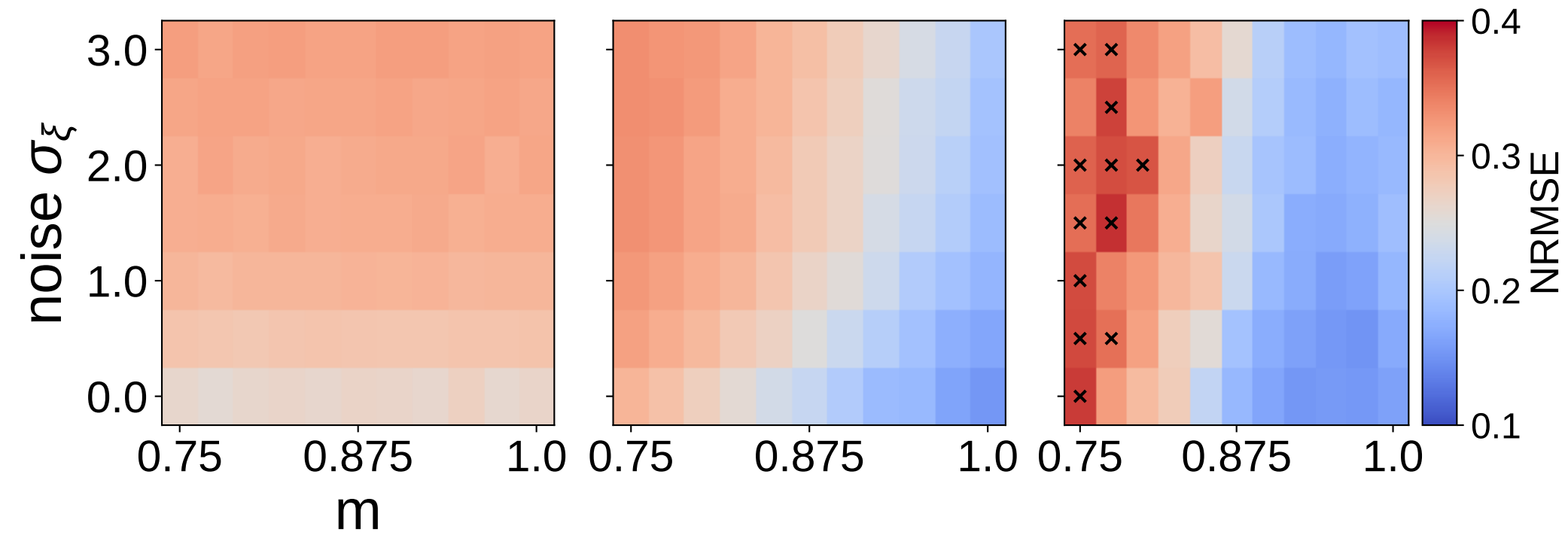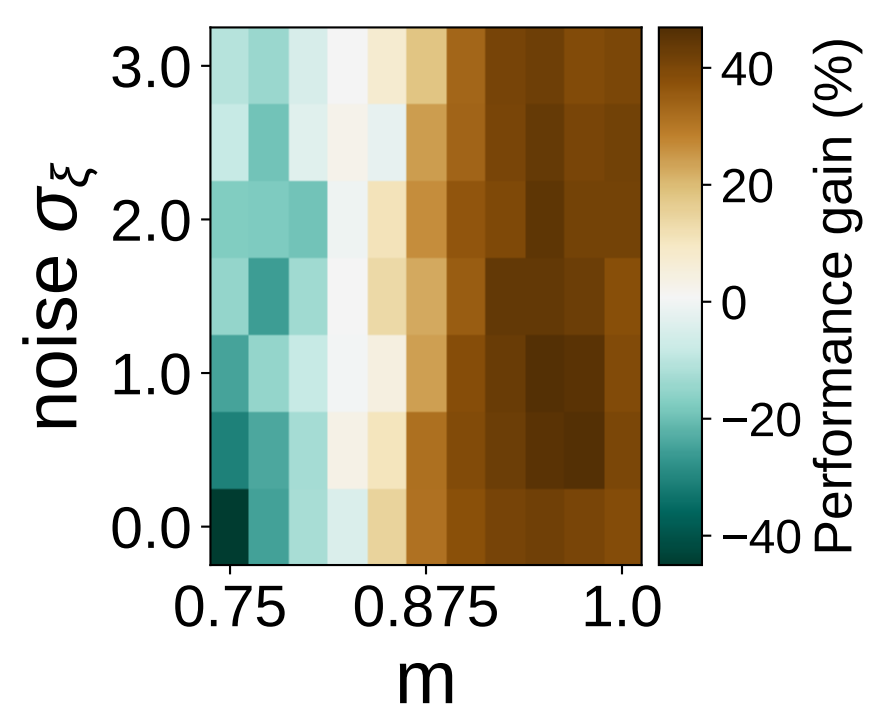

Supplement: Figure 1—source data 1. [file elife-77009-fig1-data1.zip › figure1/plots/fig1s1.pdf]

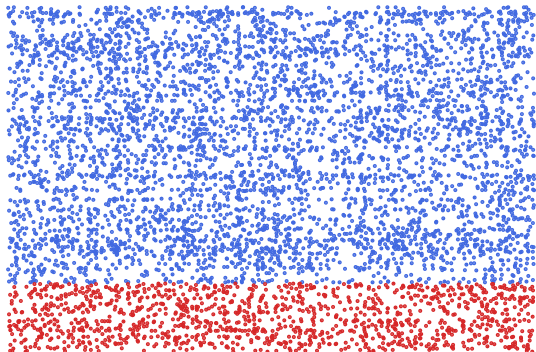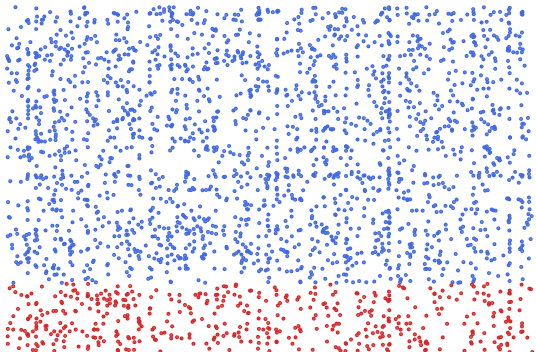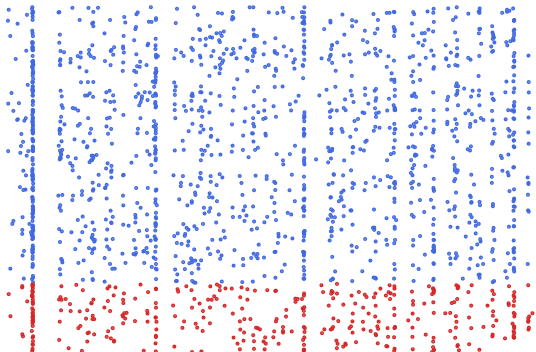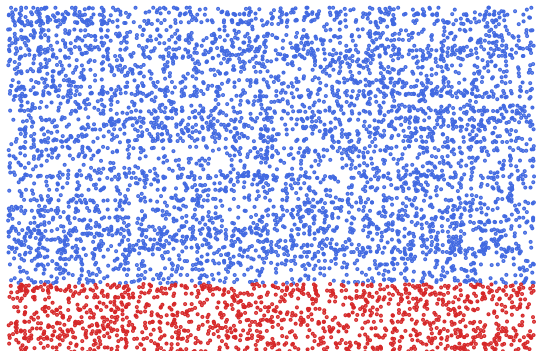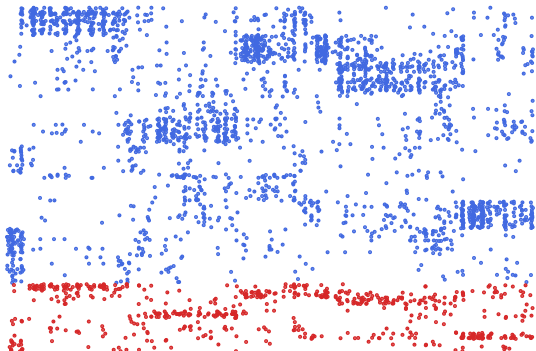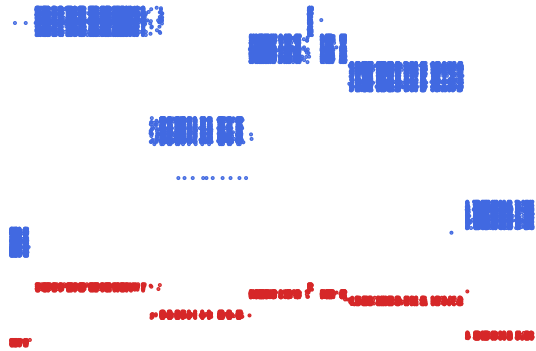

Supplement: Figure 2—source data 1. [file elife-77009-fig2-data1.zip › figure2/plots/fig2_a.pdf]

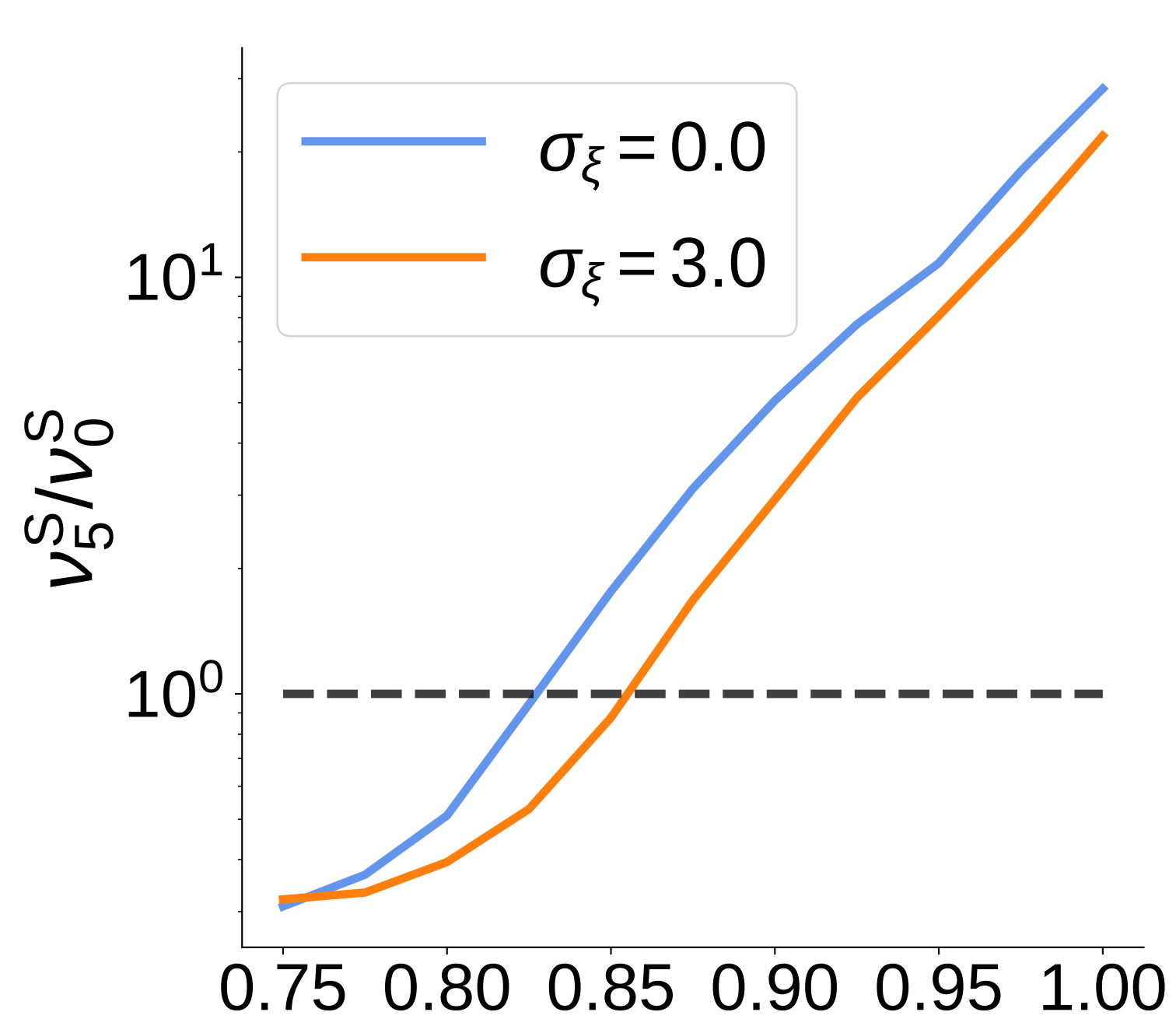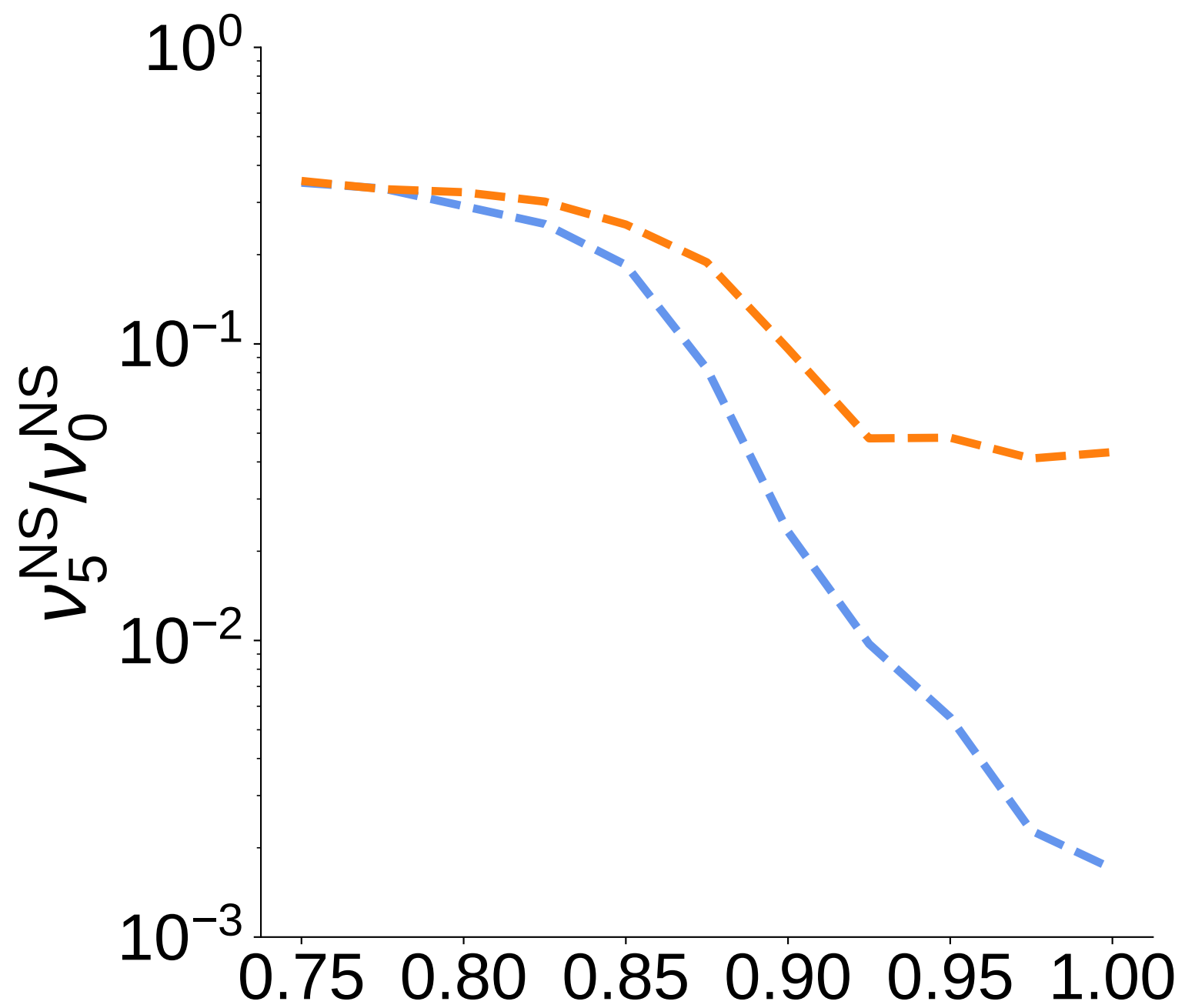

Supplement: Figure 2—source data 1. [file elife-77009-fig2-data1.zip › figure2/plots/fig2_b.pdf]

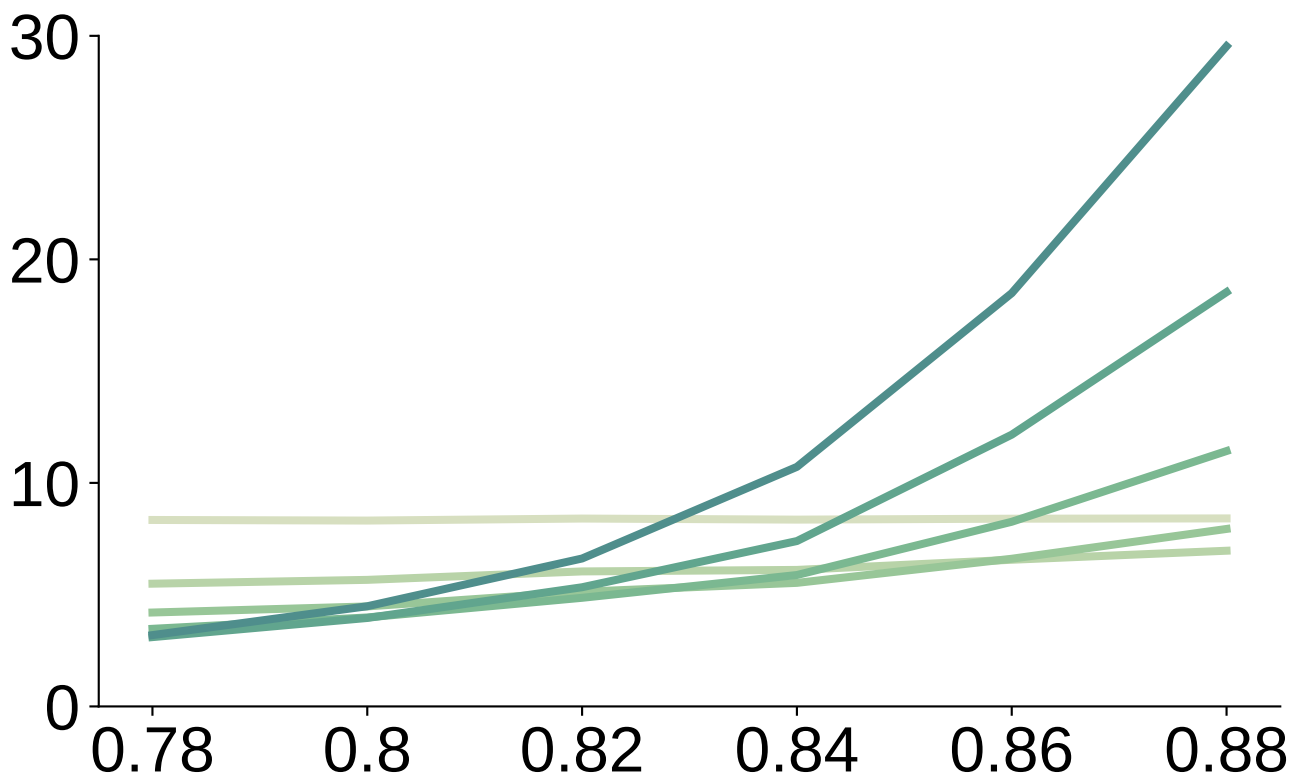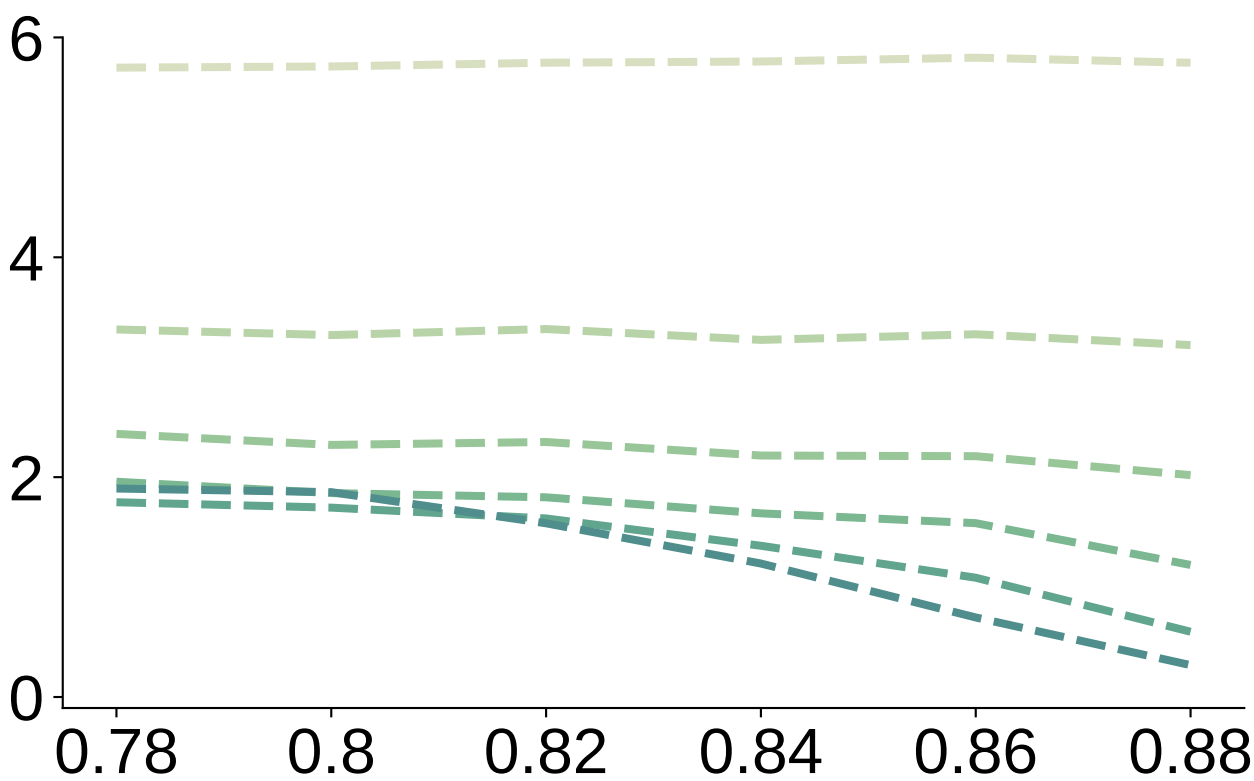

Supplement: Figure 2—source data 1. [file elife-77009-fig2-data1.zip › figure2/plots/fig2_c_sim.pdf]

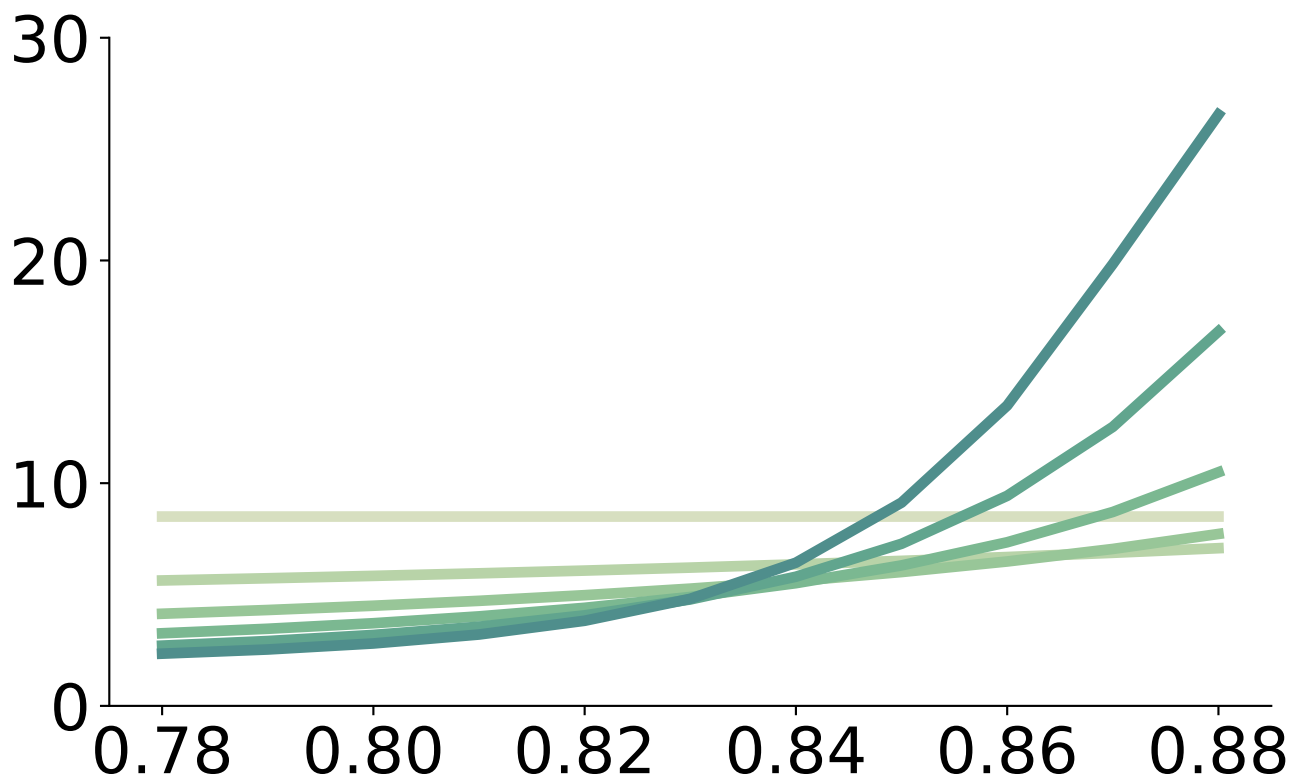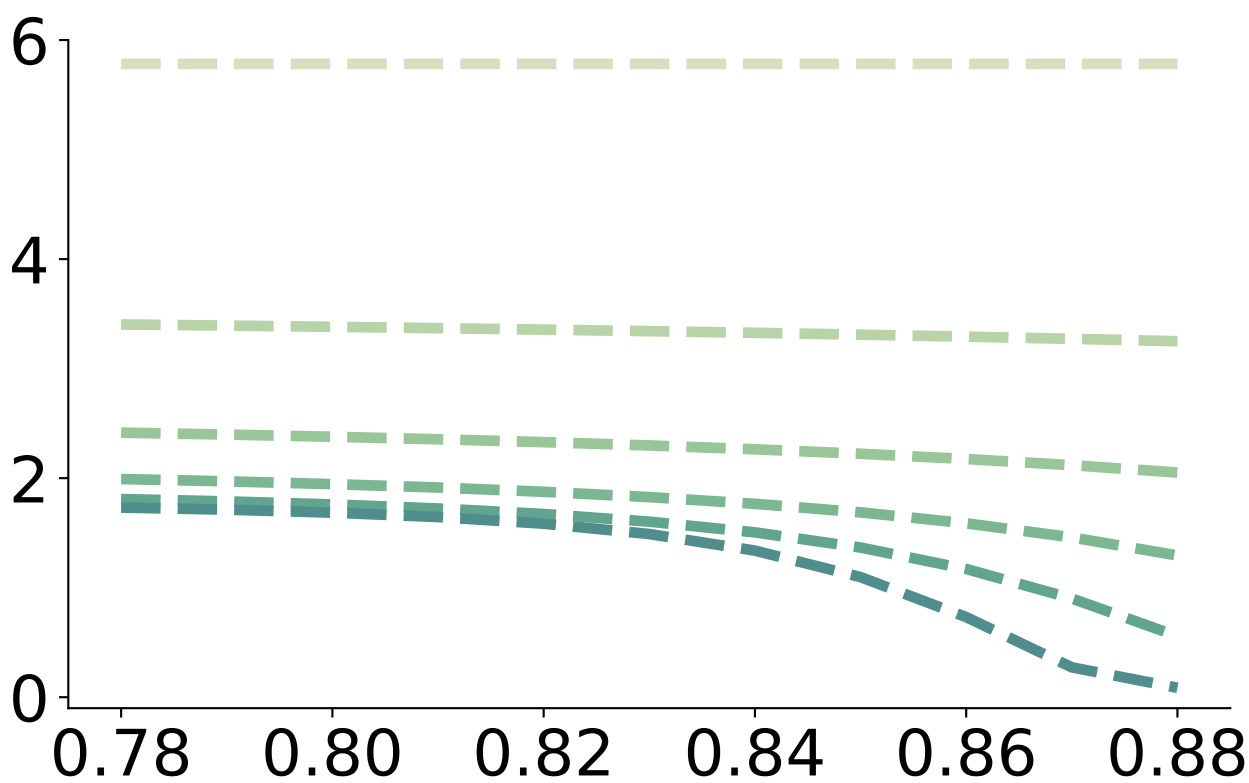

Supplement: Figure 2—source data 1. [file elife-77009-fig2-data1.zip › figure2/plots/fig2_c_theory.pdf]

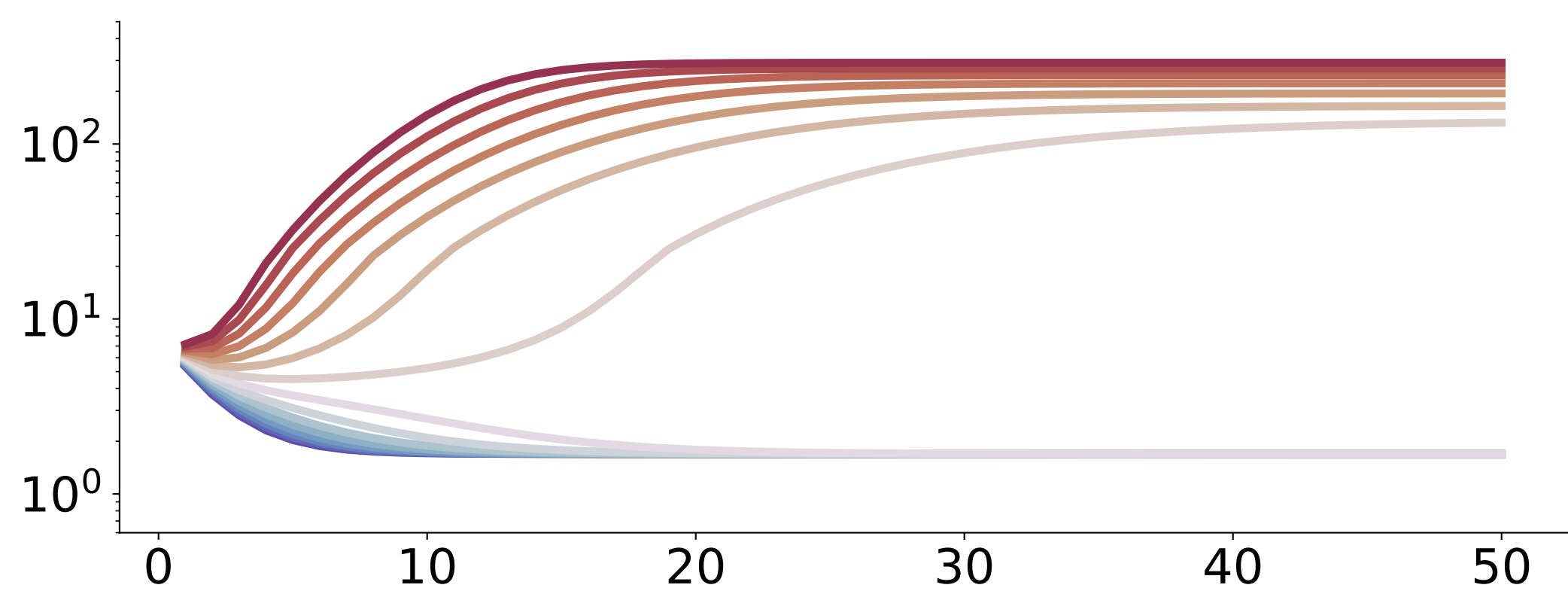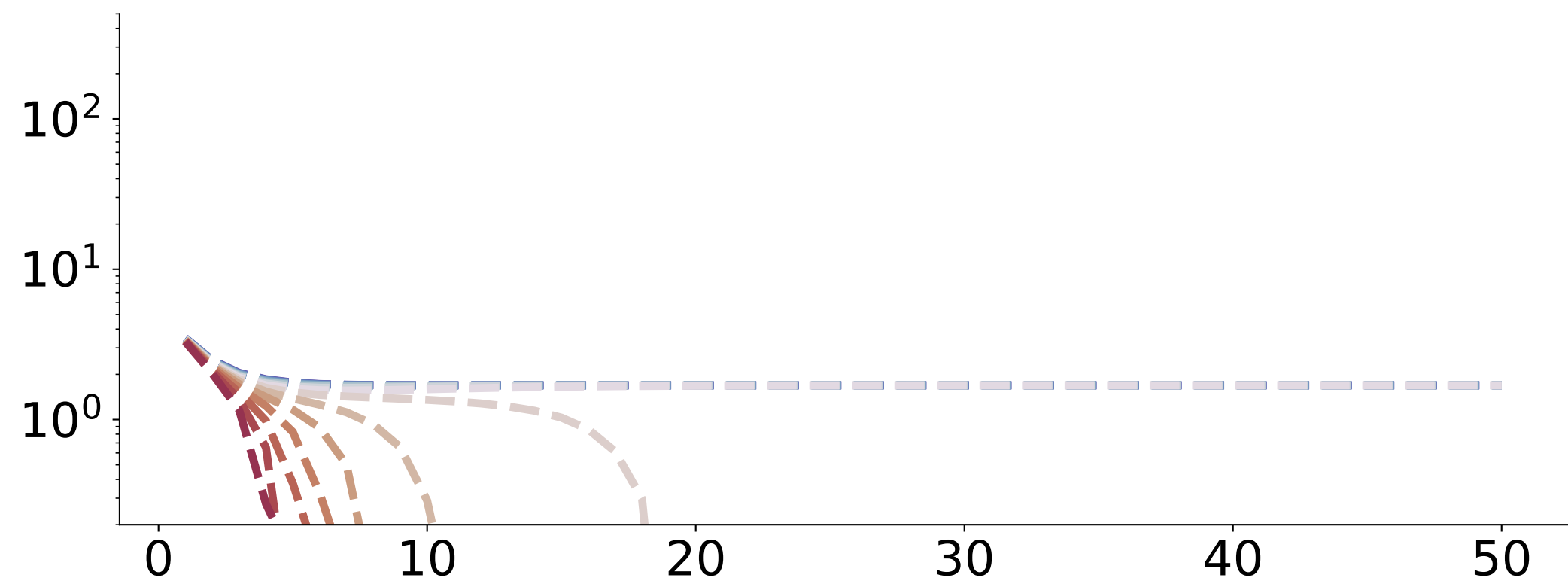

Supplement: Figure 2—source data 1. [file elife-77009-fig2-data1.zip › figure2/plots/fig2_d.pdf]

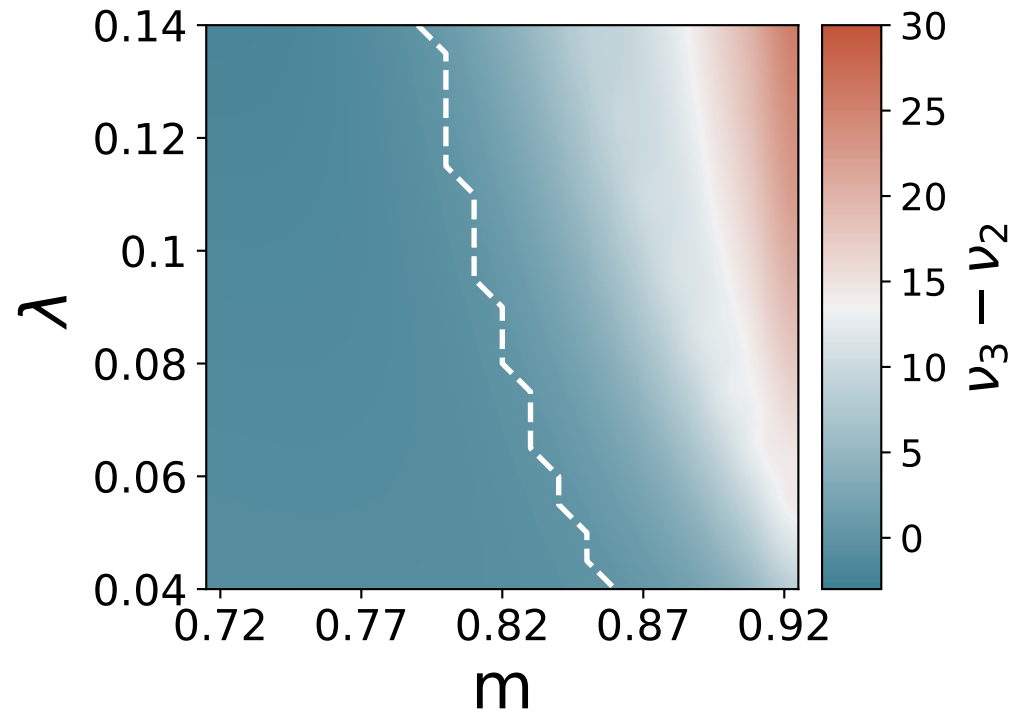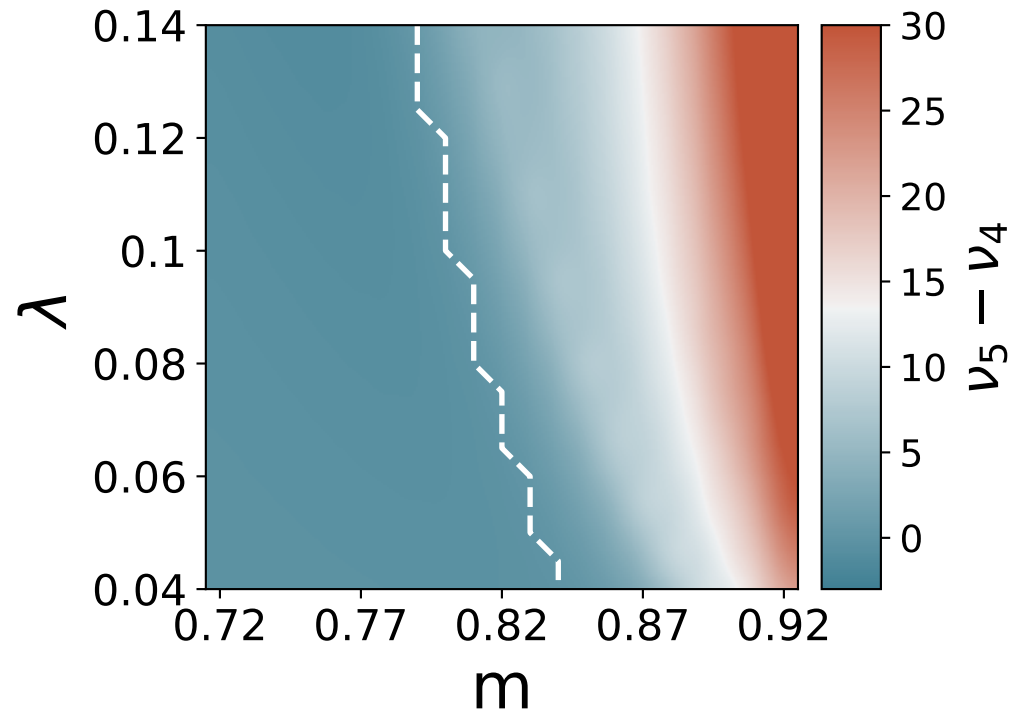

Supplement: Figure 2—source data 1. [file elife-77009-fig2-data1.zip › figure2/plots/fig2_s1.pdf]

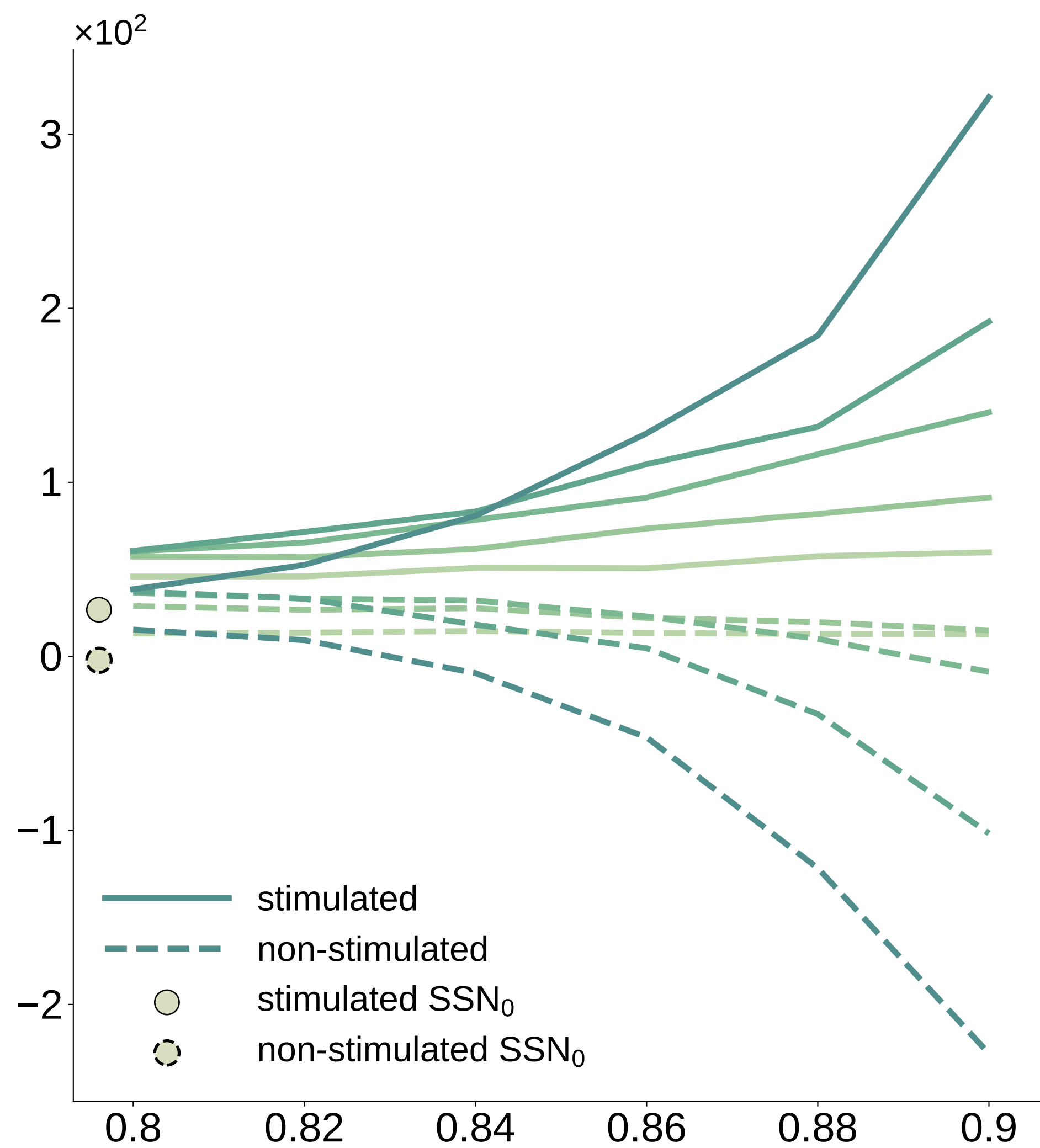

Supplement: Figure 3—source data 1. [file elife-77009-fig3-data1.zip › figure3/plots/fig3_a.pdf]

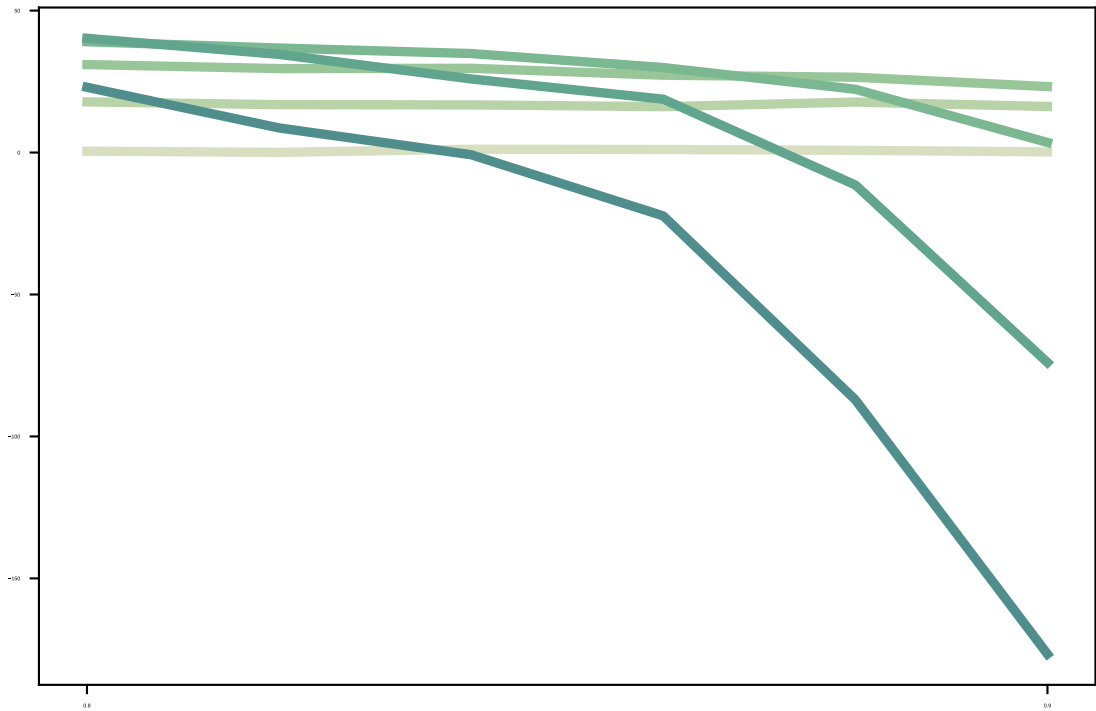

Supplement: Figure 3—source data 1. [file elife-77009-fig3-data1.zip › figure3/plots/fig3_a_inset.pdf]

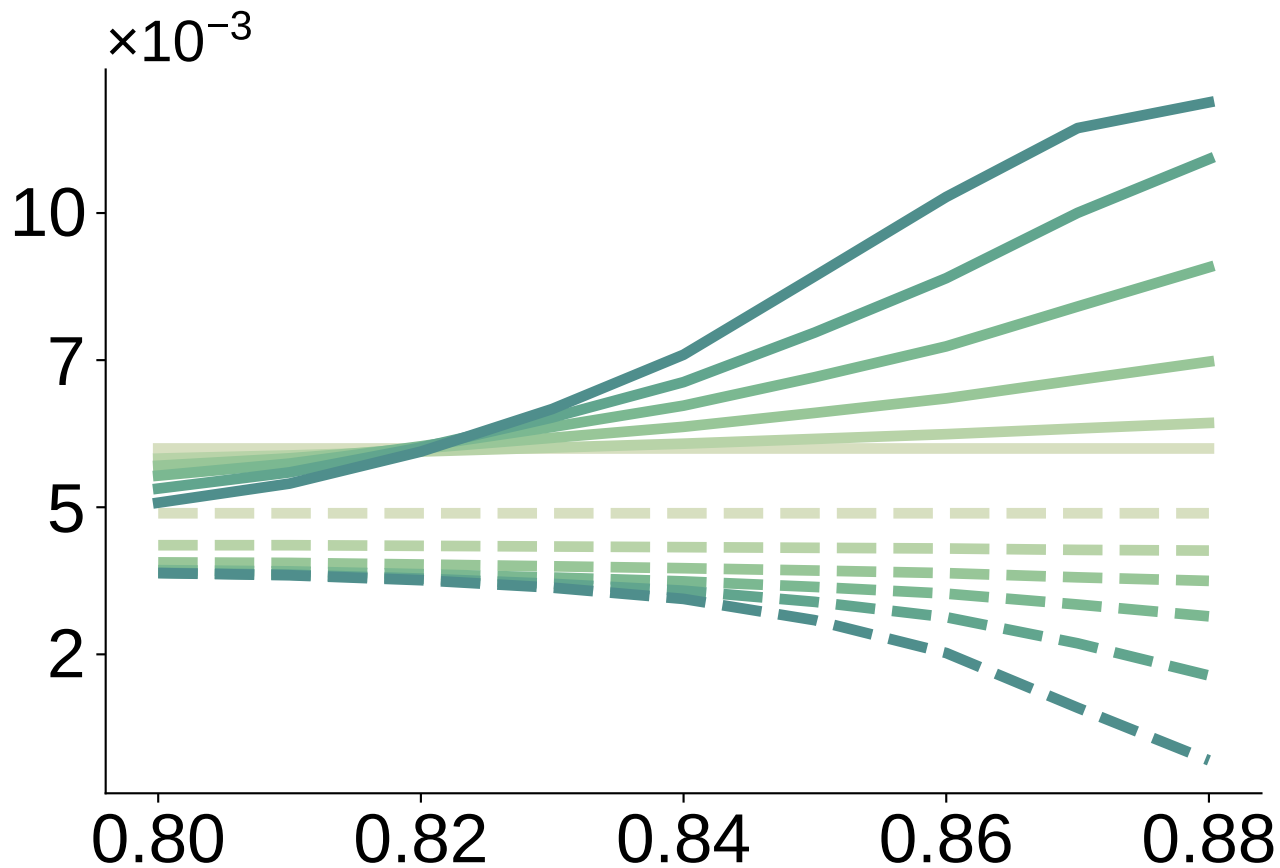

Supplement: Figure 3—source data 1. [file elife-77009-fig3-data1.zip › figure3/plots/fig3_b.pdf]

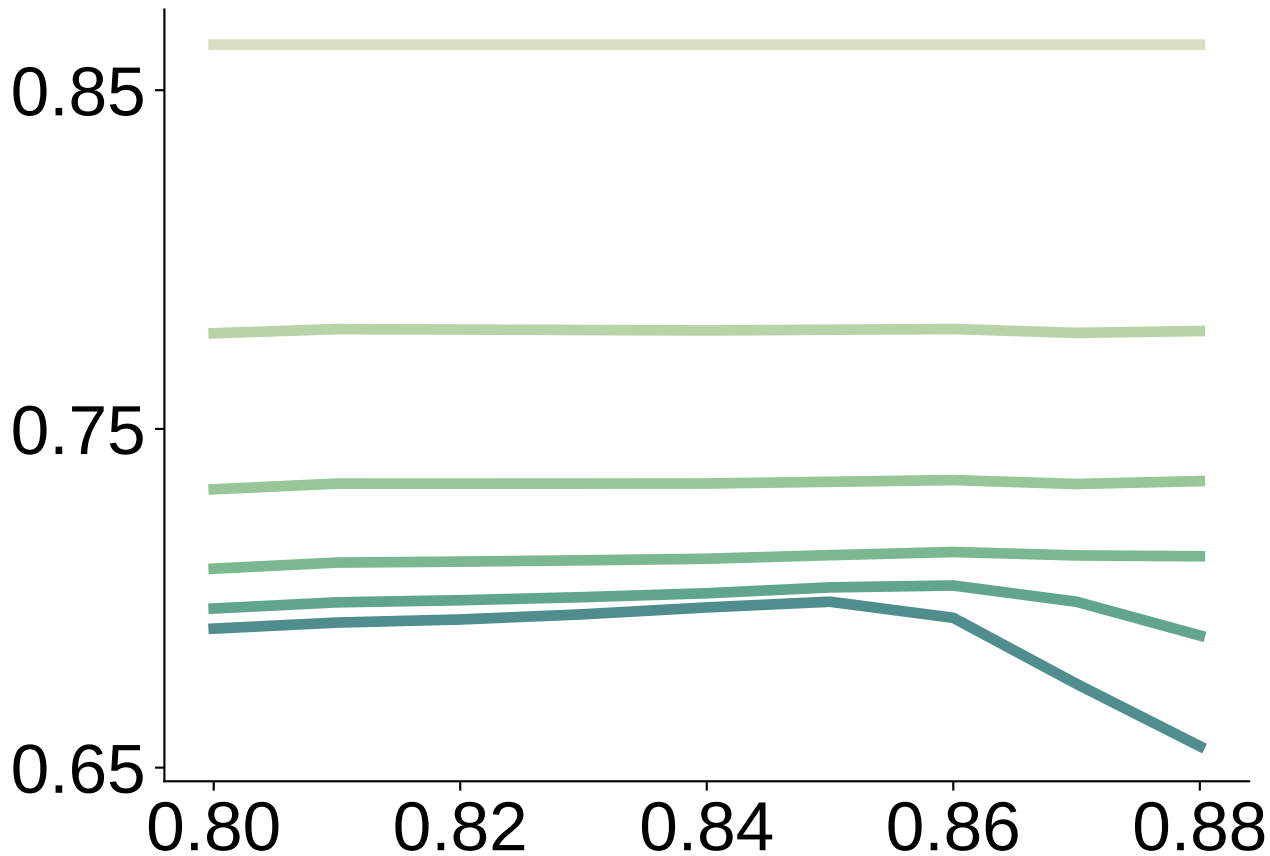

Supplement: Figure 3—source data 1. [file elife-77009-fig3-data1.zip › figure3/plots/fig3_c.pdf]

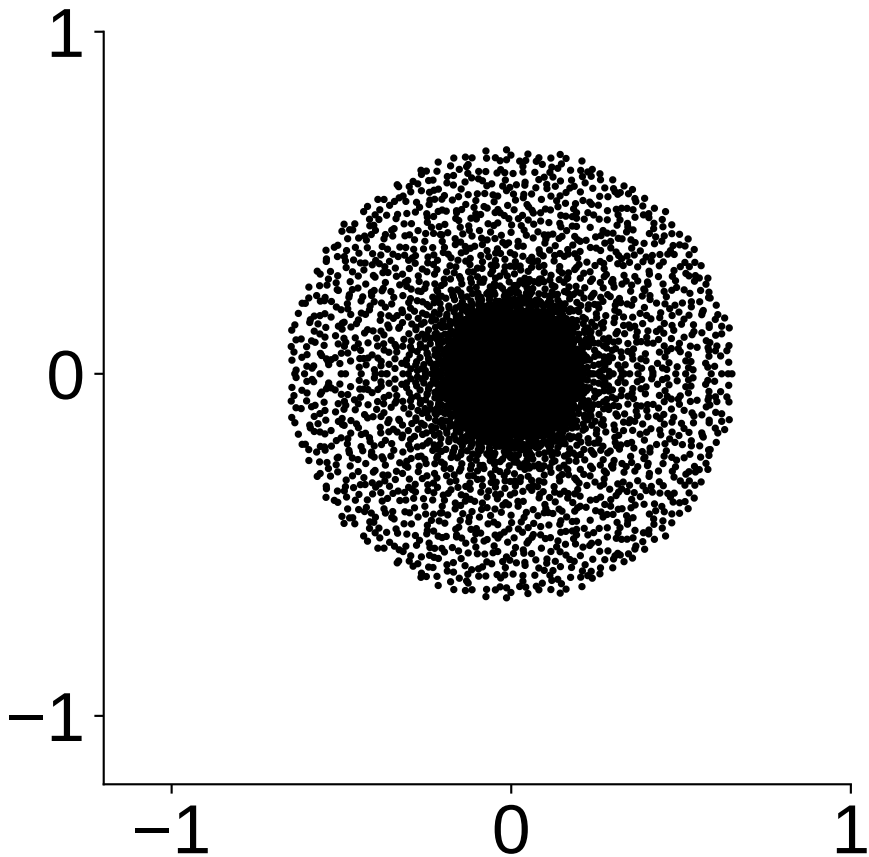

Supplement: Figure 3—source data 1. [file elife-77009-fig3-data1.zip › figure3/plots/fig3_d_m=0.8.pdf]

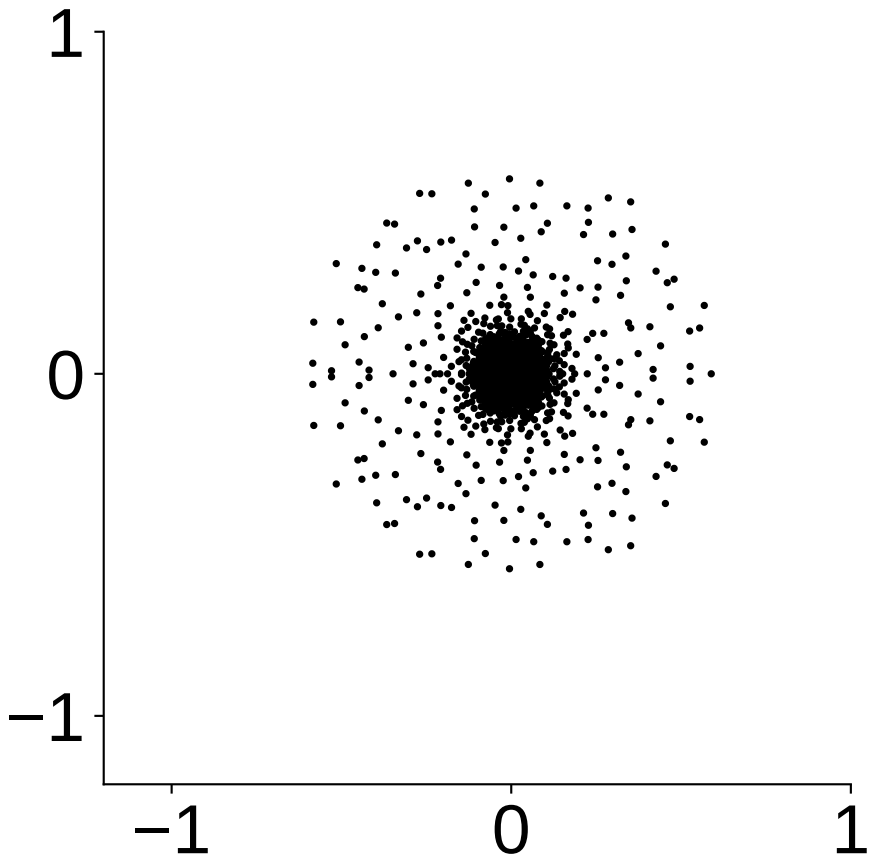

Supplement: Figure 3—source data 1. [file elife-77009-fig3-data1.zip › figure3/plots/fig3_d_m=0.9.pdf]

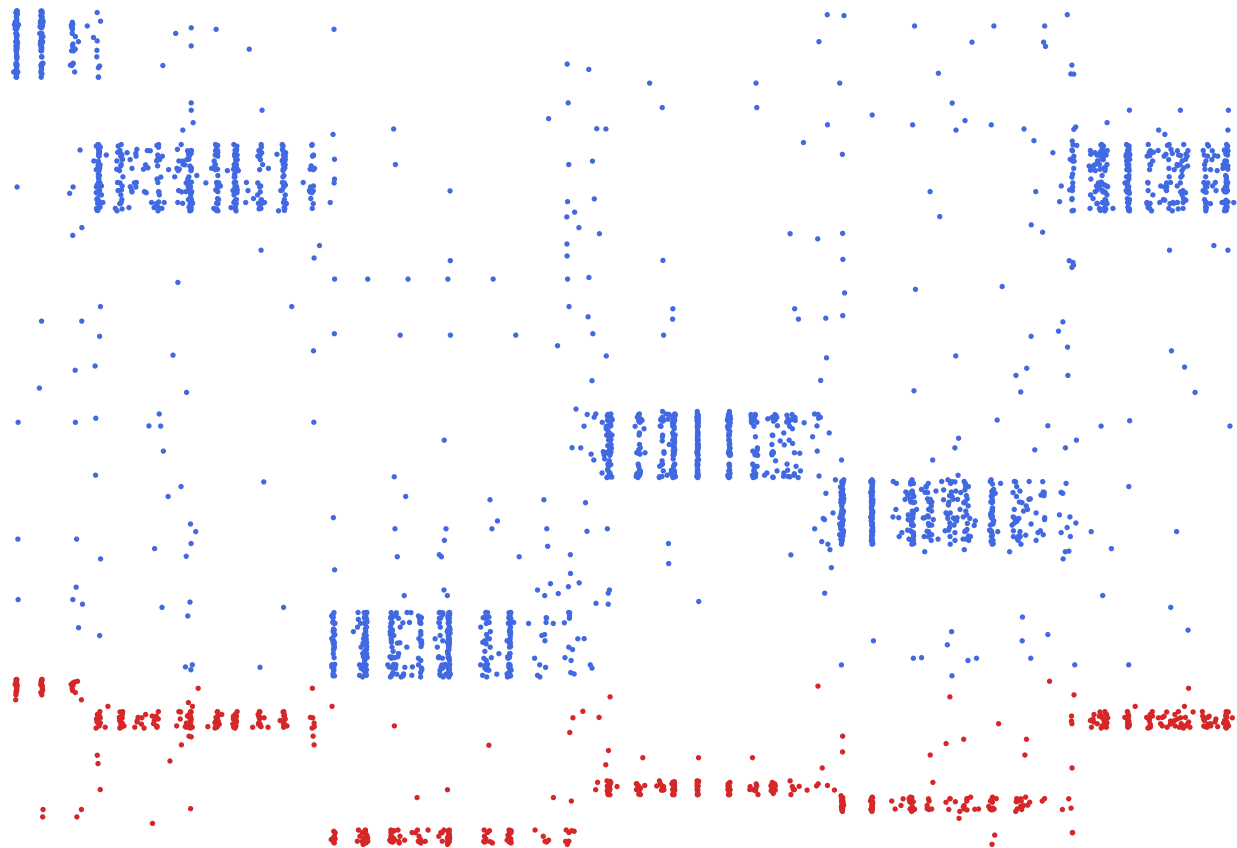

Supplement: Figure 4—source data 1. [file elife-77009-fig4-data1.zip › figure4/plots/fig4_a.pdf]

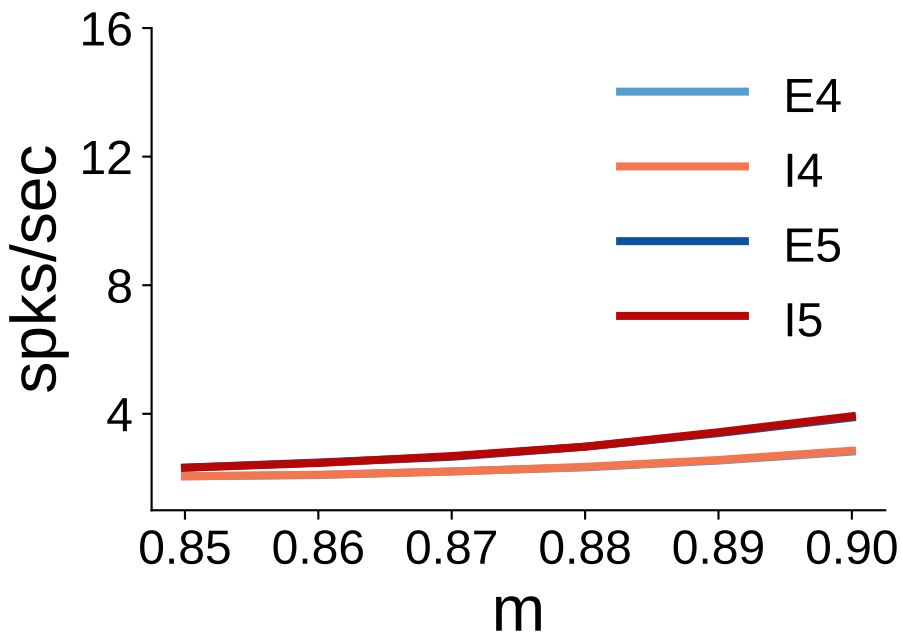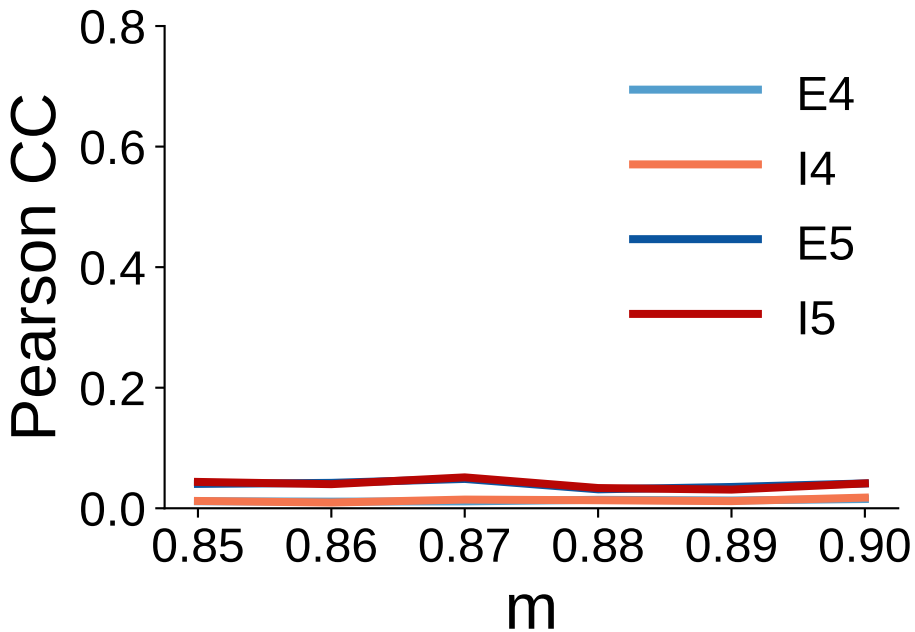

Supplement: Figure 4—source data 1. [file elife-77009-fig4-data1.zip › figure4/plots/fig4_s1_a.pdf]

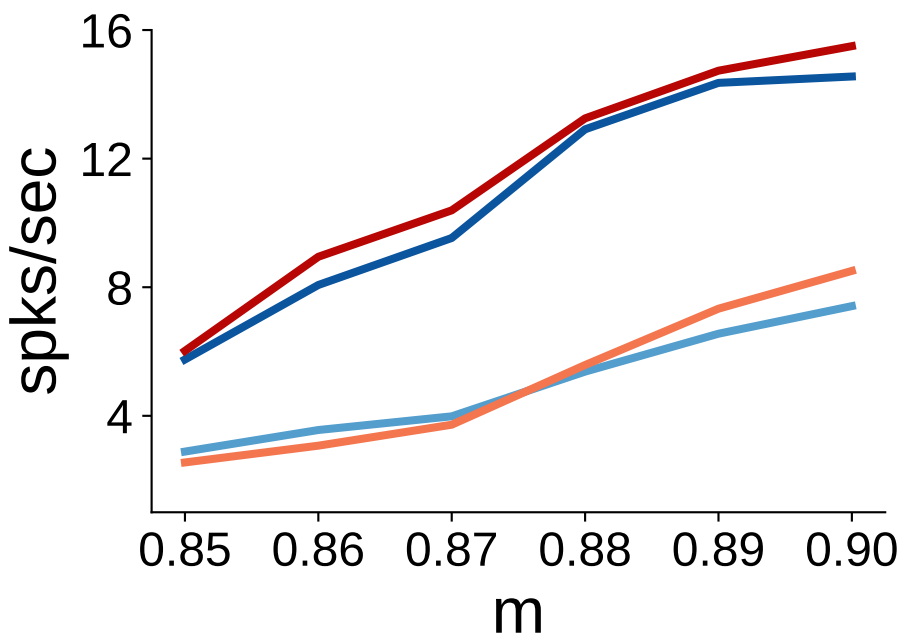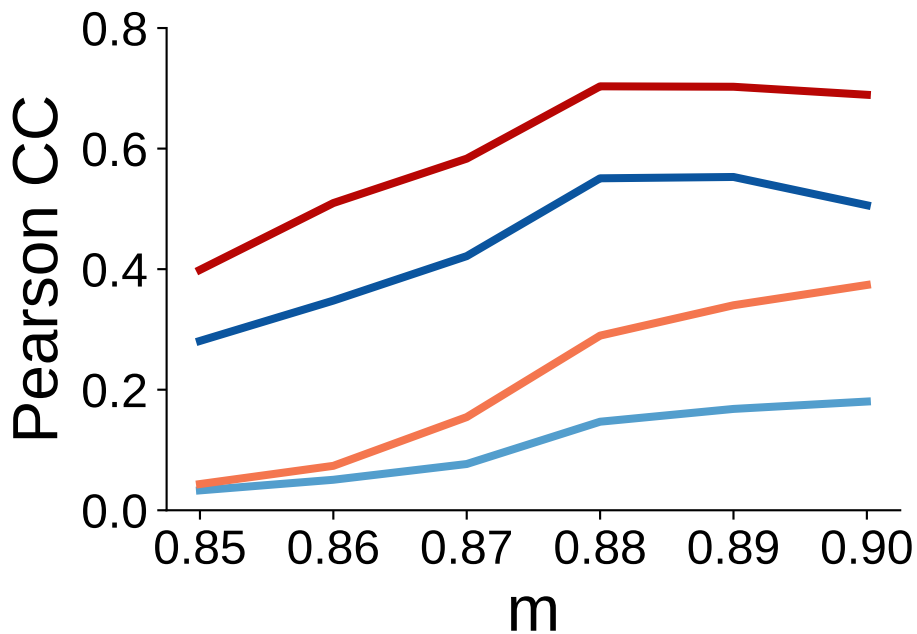

Supplement: Figure 4—source data 1. [file elife-77009-fig4-data1.zip › figure4/plots/fig4_s1_b.pdf]

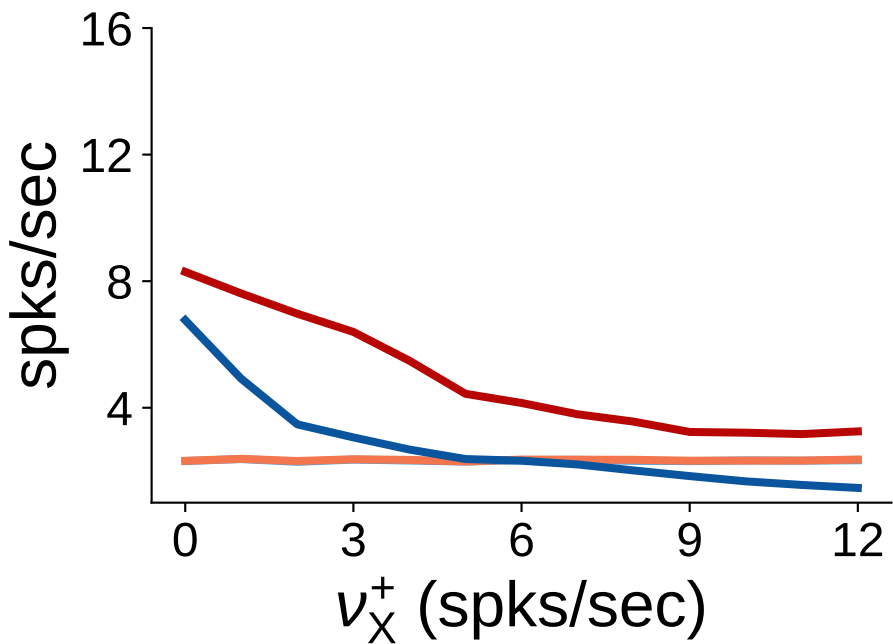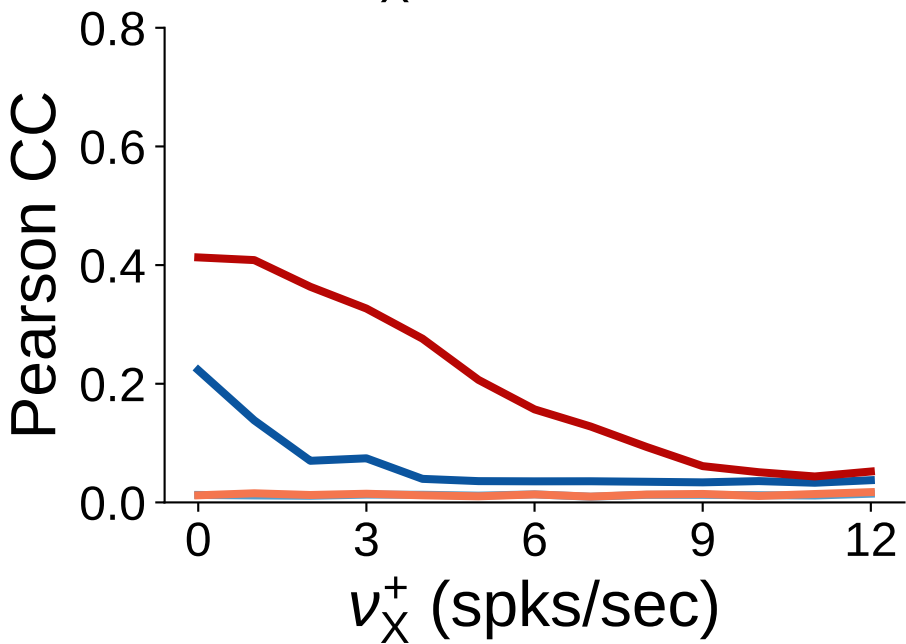

Supplement: Figure 4—source data 1. [file elife-77009-fig4-data1.zip › figure4/plots/fig4_s1_c.pdf]

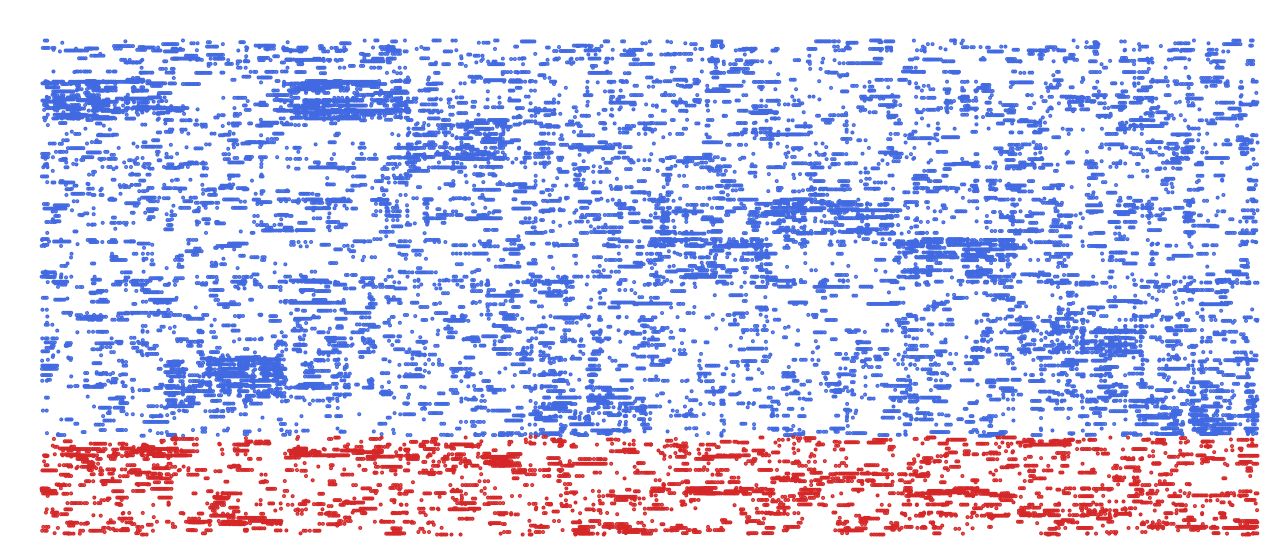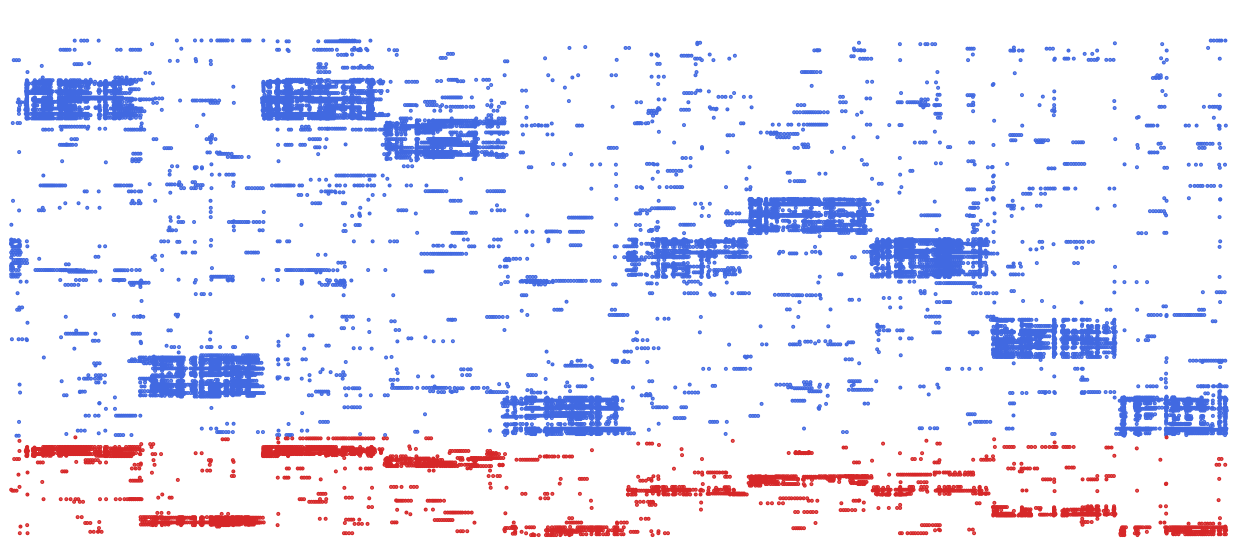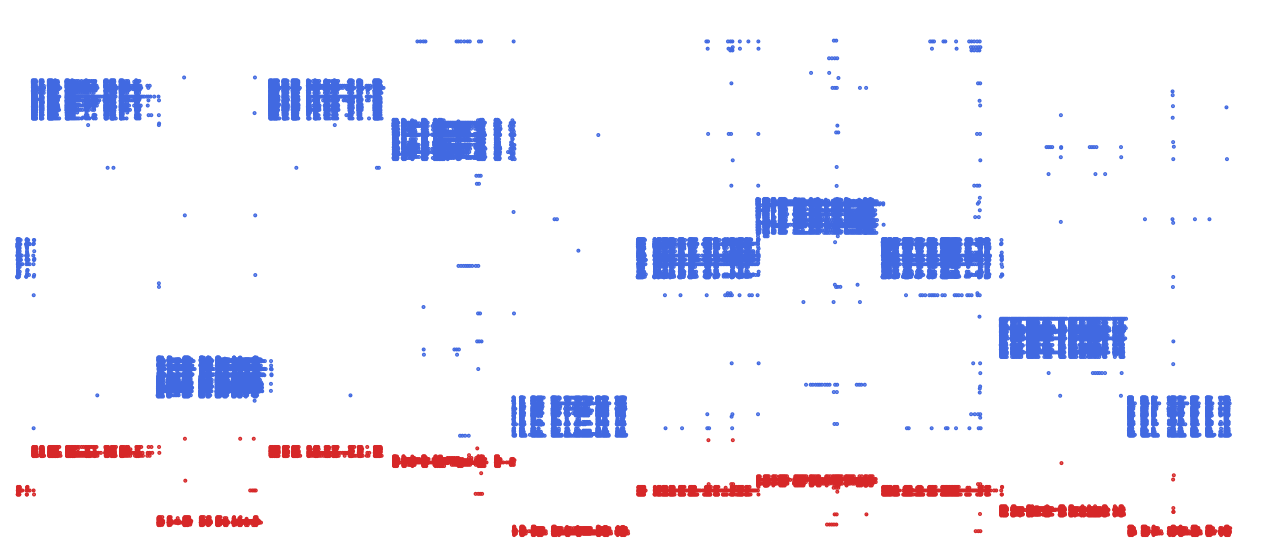

Supplement: Figure 5—source data 1. [file elife-77009-fig5-data1.zip › figure5/plots/fig5_a_raster.pdf]

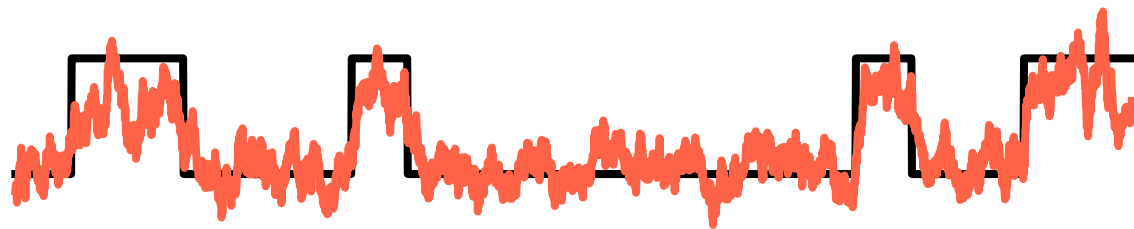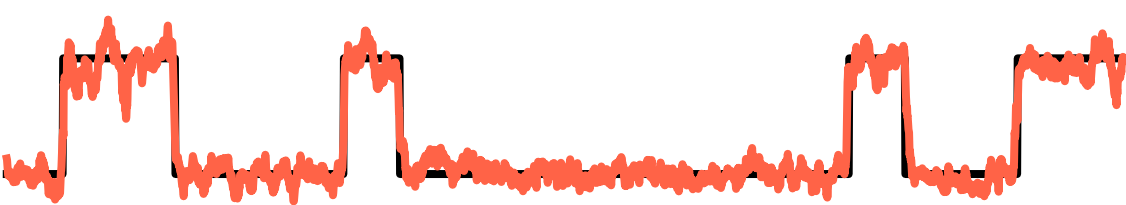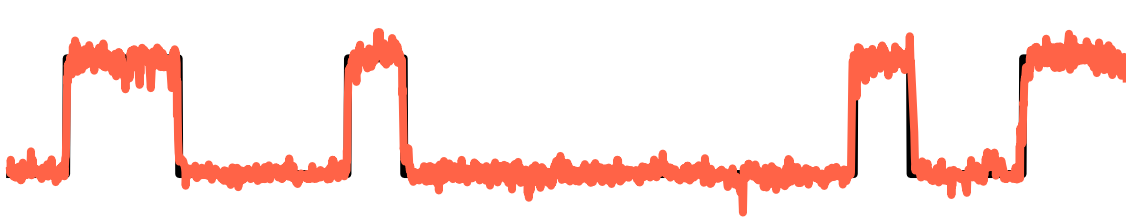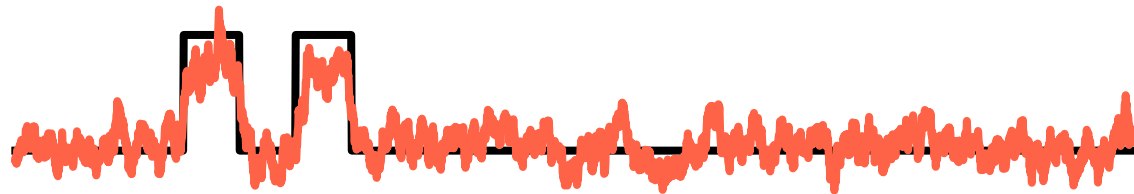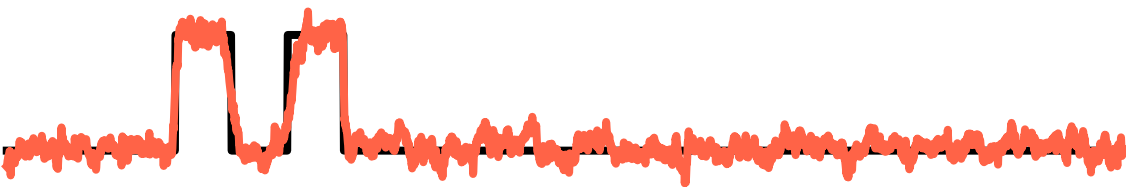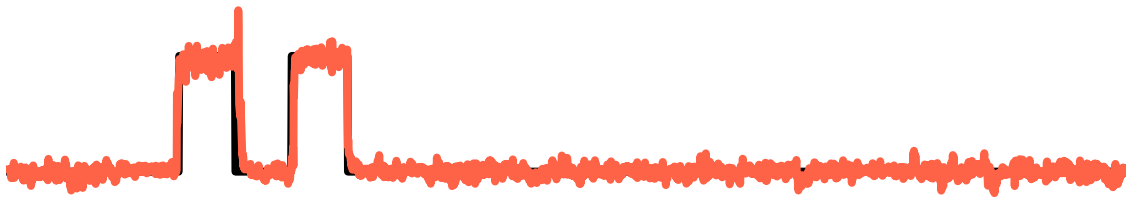

Supplement: Figure 5—source data 1. [file elife-77009-fig5-data1.zip › figure5/plots/fig5_a_readout.pdf]

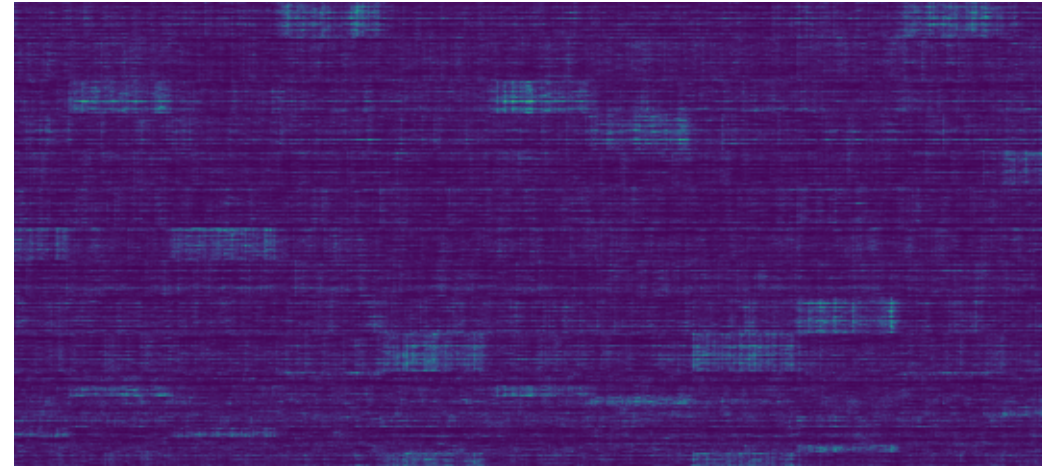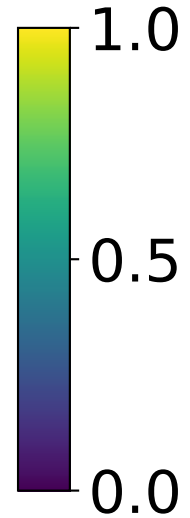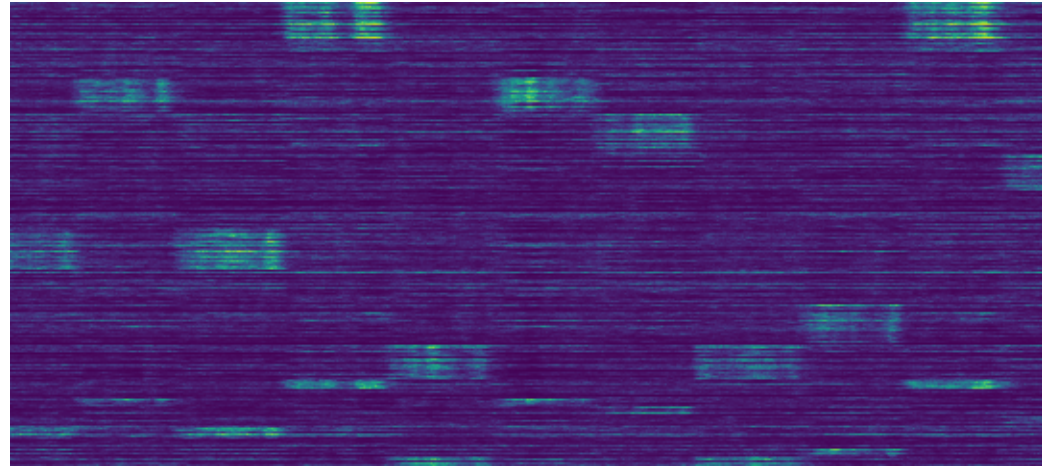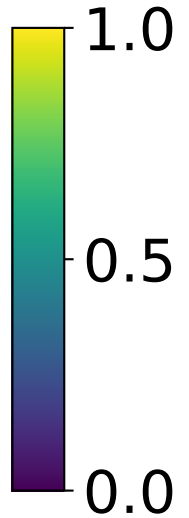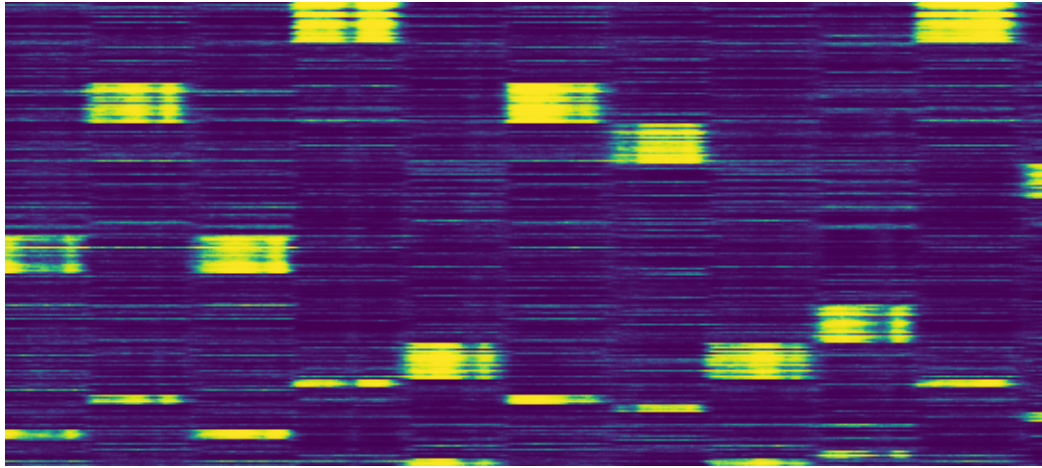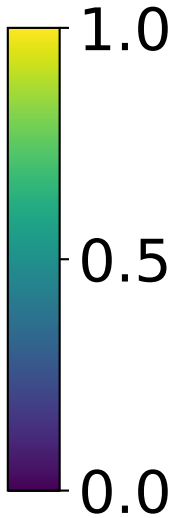

Supplement: Figure 5—source data 1. [file elife-77009-fig5-data1.zip › figure5/plots/fig5_b_activity.pdf]

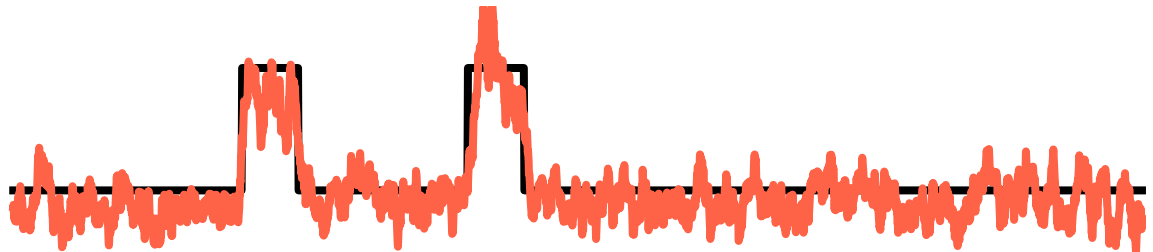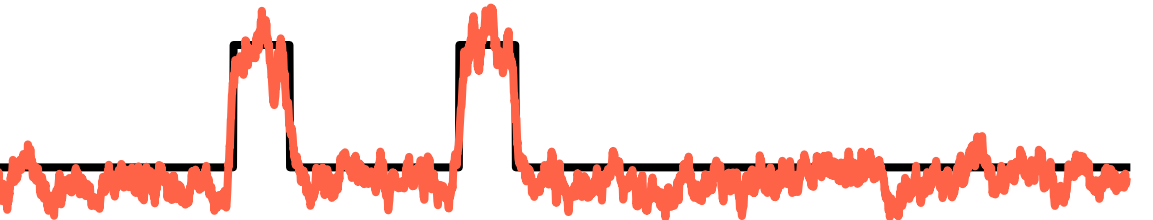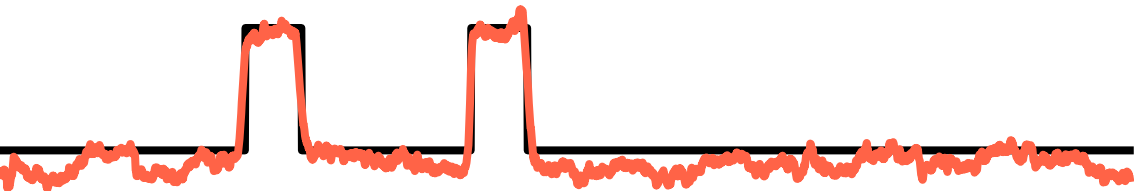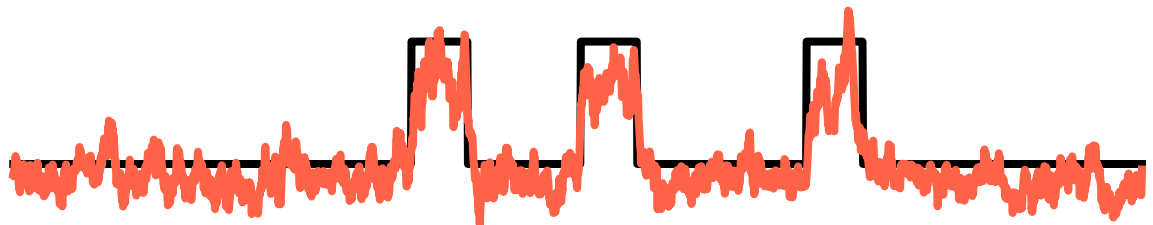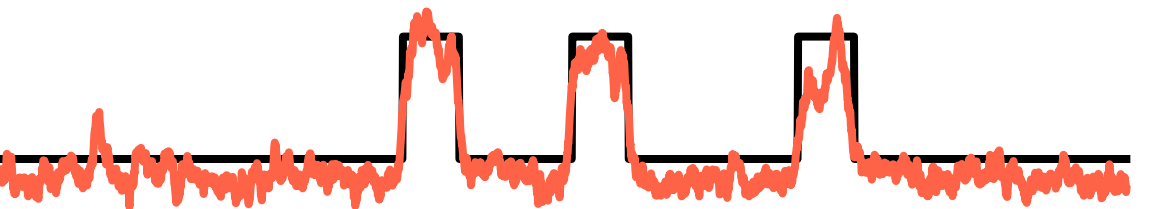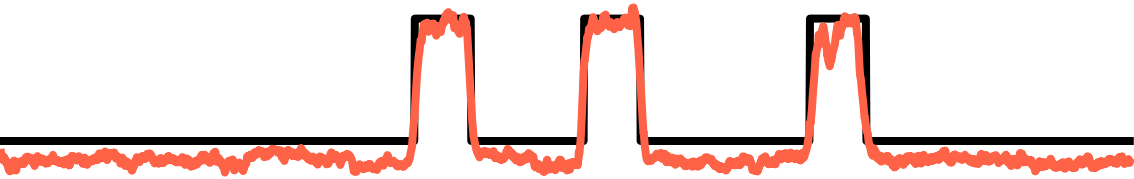

Supplement: Figure 5—source data 1. [file elife-77009-fig5-data1.zip › figure5/plots/fig5_b_readout.pdf]

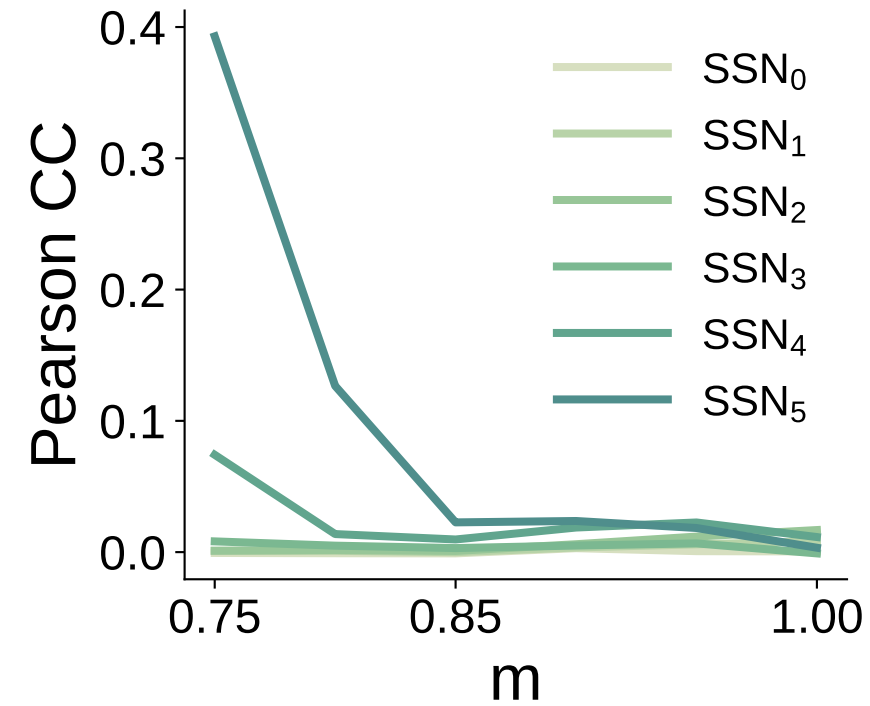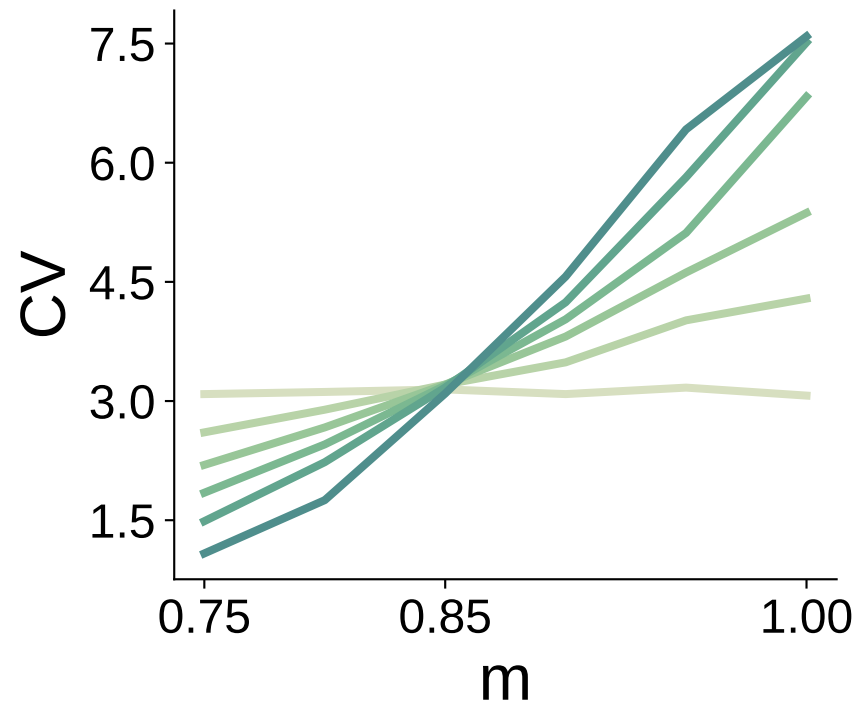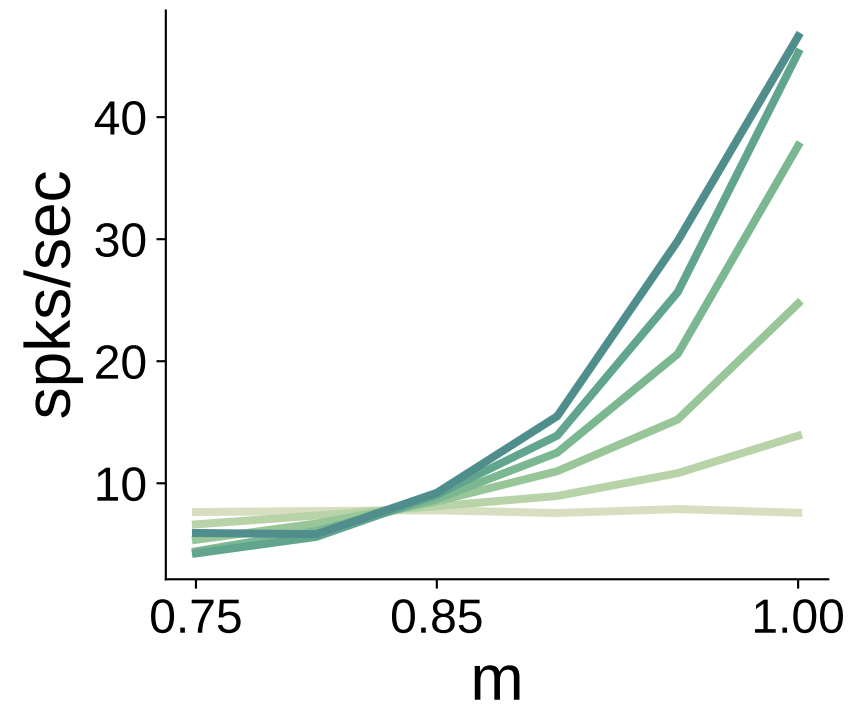

Supplement: Figure 5—source data 1. [file elife-77009-fig5-data1.zip › figure5/plots/fig5_s1.pdf]

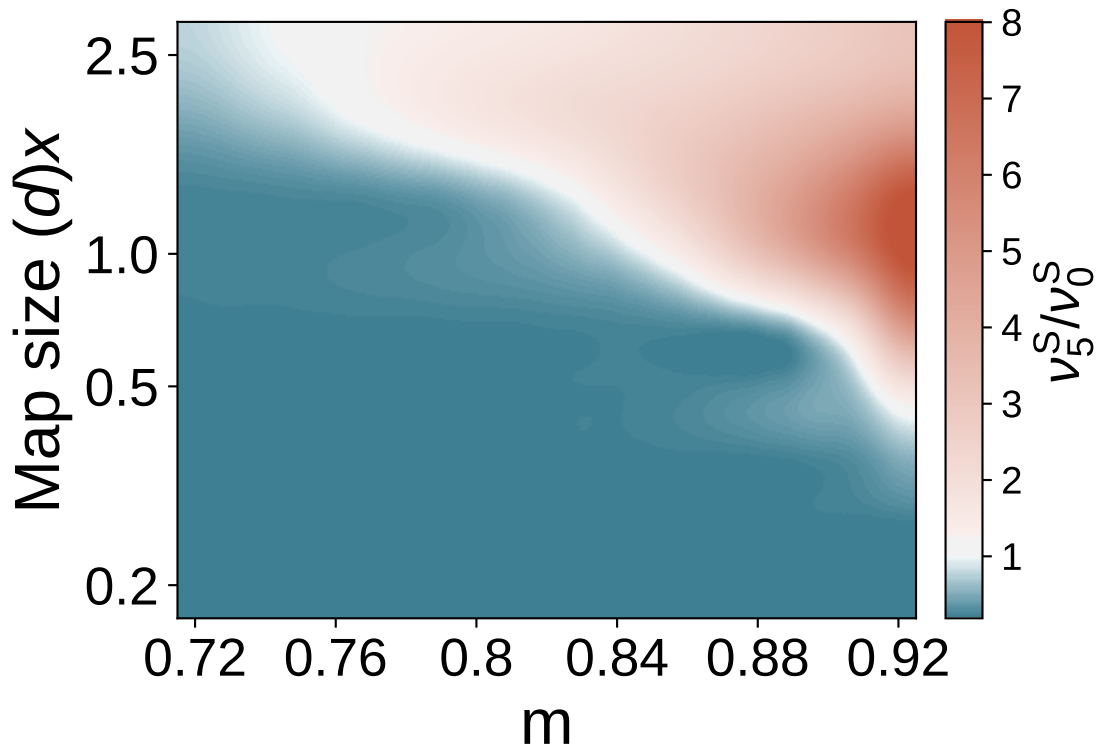

Supplement: Figure 6—source data 1. [file elife-77009-fig6-data1.zip › figure6/plots/fig6_a.pdf]

% gain

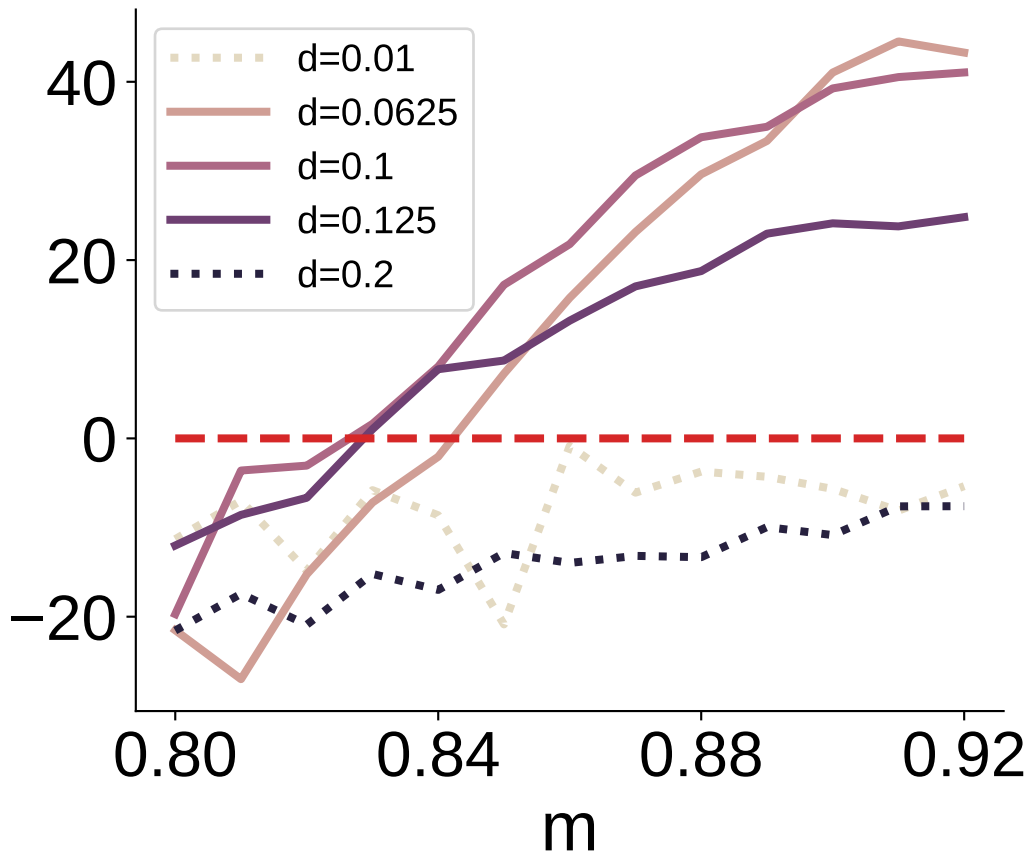

Supplement: Figure 6—source data 1. [file elife-77009-fig6-data1.zip › figure6/plots/fig6_b.pdf]

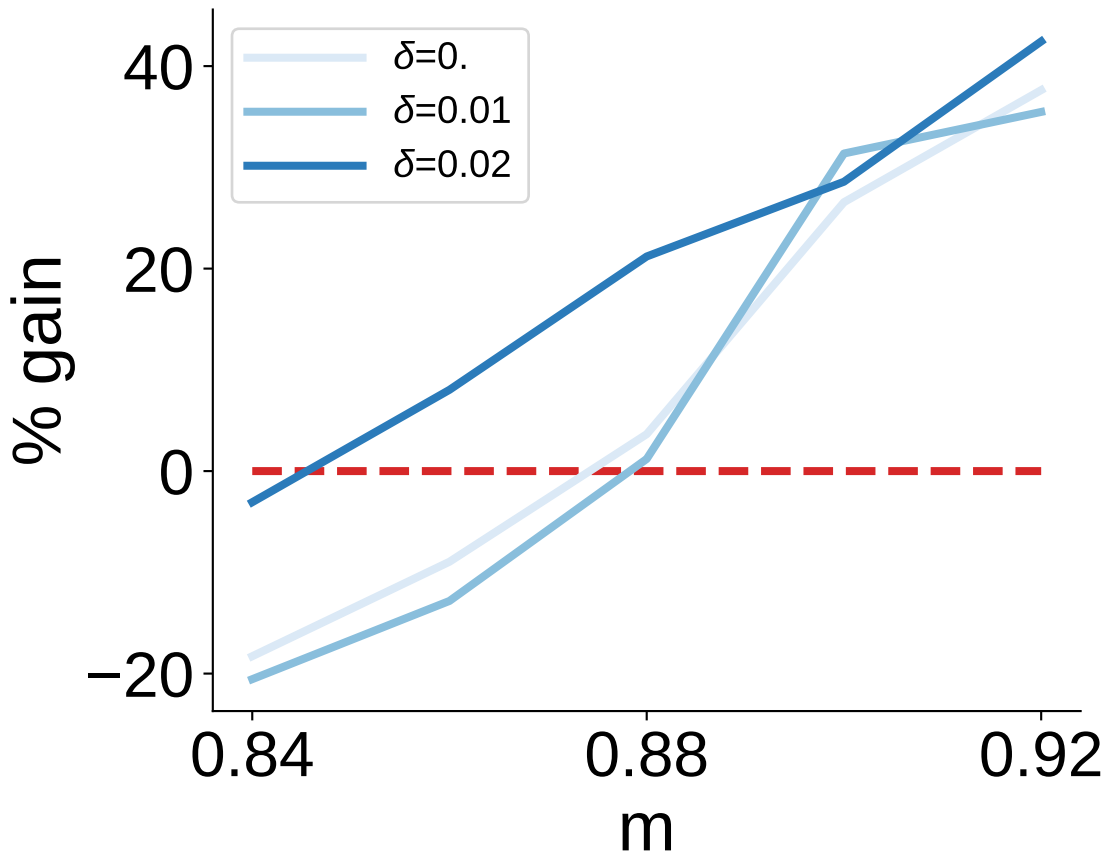

Supplement: Figure 6—source data 1. [file elife-77009-fig6-data1.zip › figure6/plots/fig6_c.pdf]

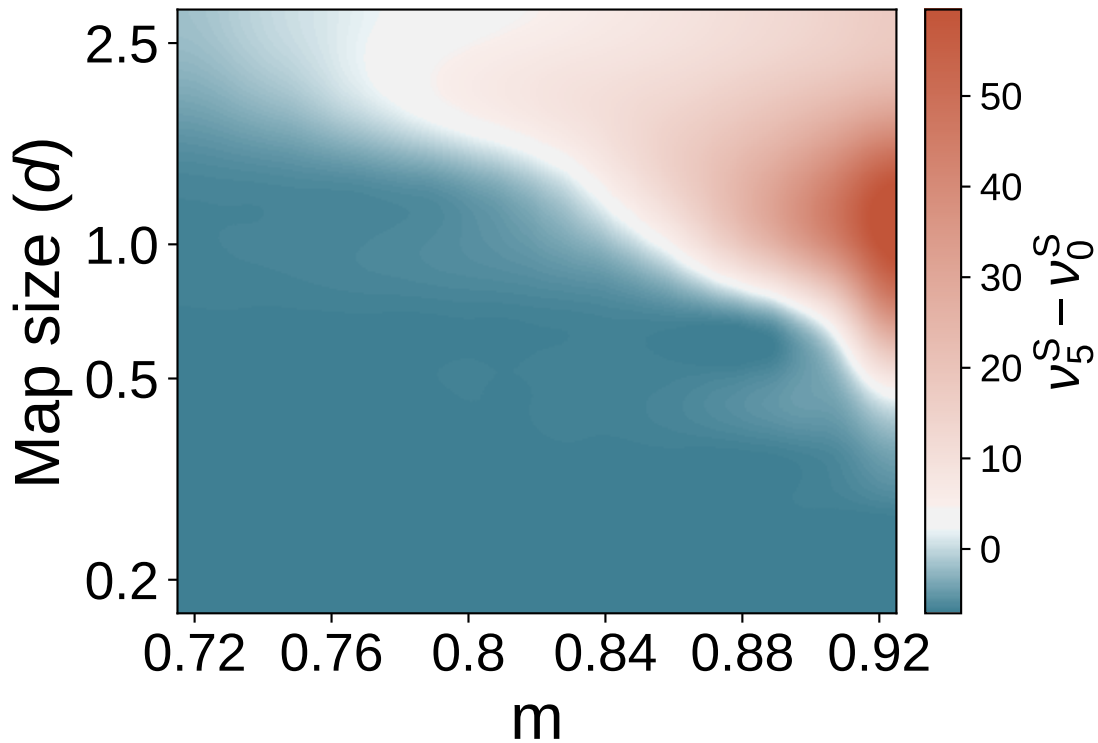

Supplement: Figure 6—source data 1. [file elife-77009-fig6-data1.zip › figure6/plots/fig6_s1_a_active.pdf]

Map size ( $d$ )

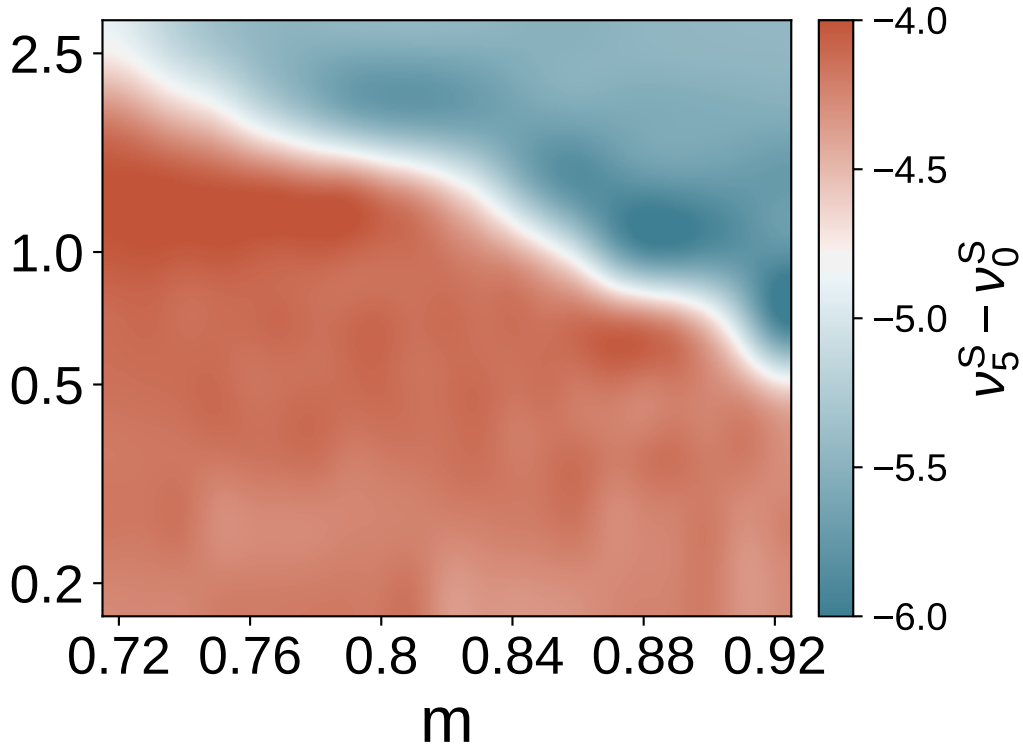

Supplement: Figure 6—source data 1. [file elife-77009-fig6-data1.zip › figure6/plots/fig6_s1_a_non_active.pdf]

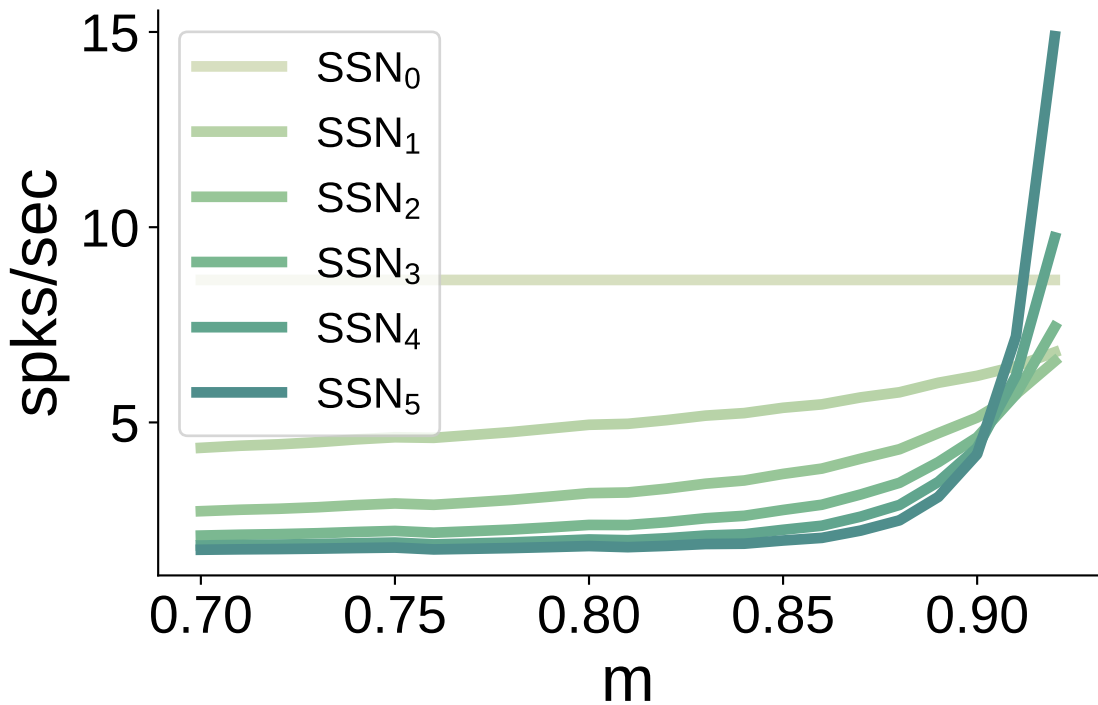

Supplement: Figure 6—source data 1. [file elife-77009-fig6-data1.zip › figure6/plots/fig6_s1_b_d=0.05.pdf]

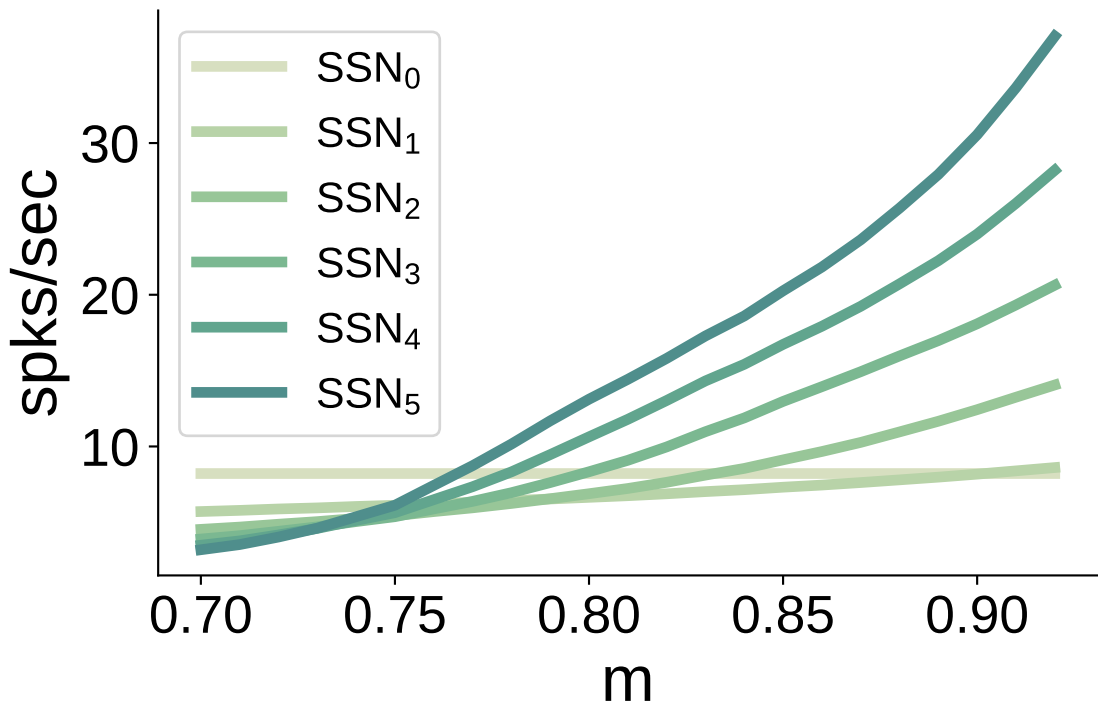

Supplement: Figure 6—source data 1. [file elife-77009-fig6-data1.zip › figure6/plots/fig6_s1_b_d=0.2.pdf]

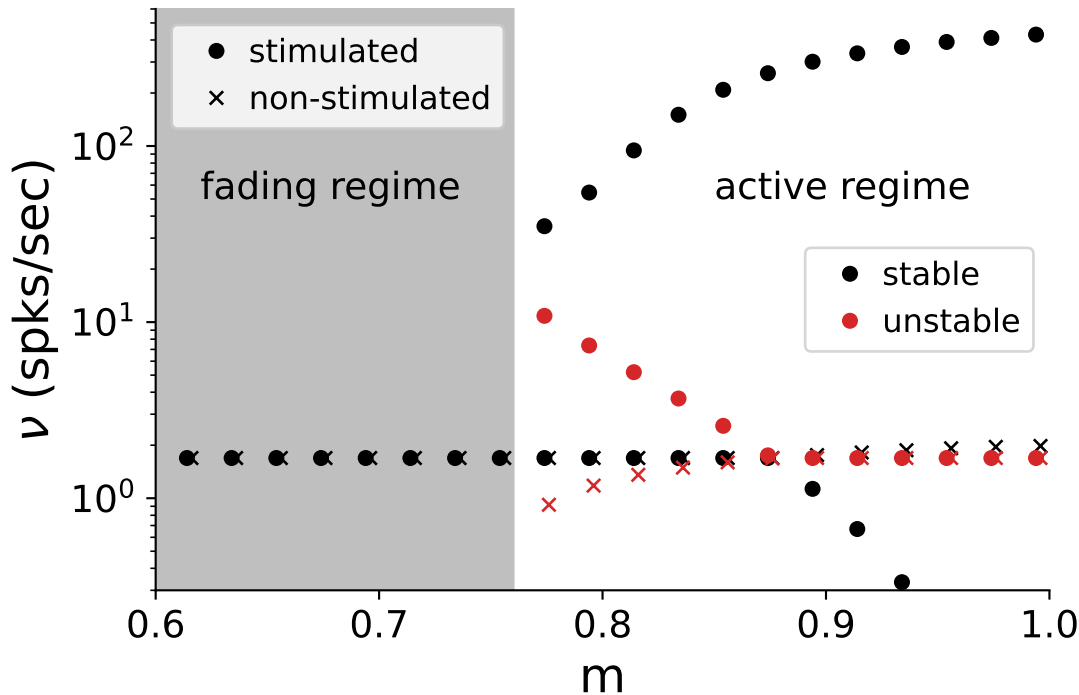

Supplement: Figure 7—source data 1. [file elife-77009-fig7-data1.zip › figure7/plots/fig7_b.pdf]

Potential

0

50

100

150

200

250

300

$\nu^S$  (spks/sec)

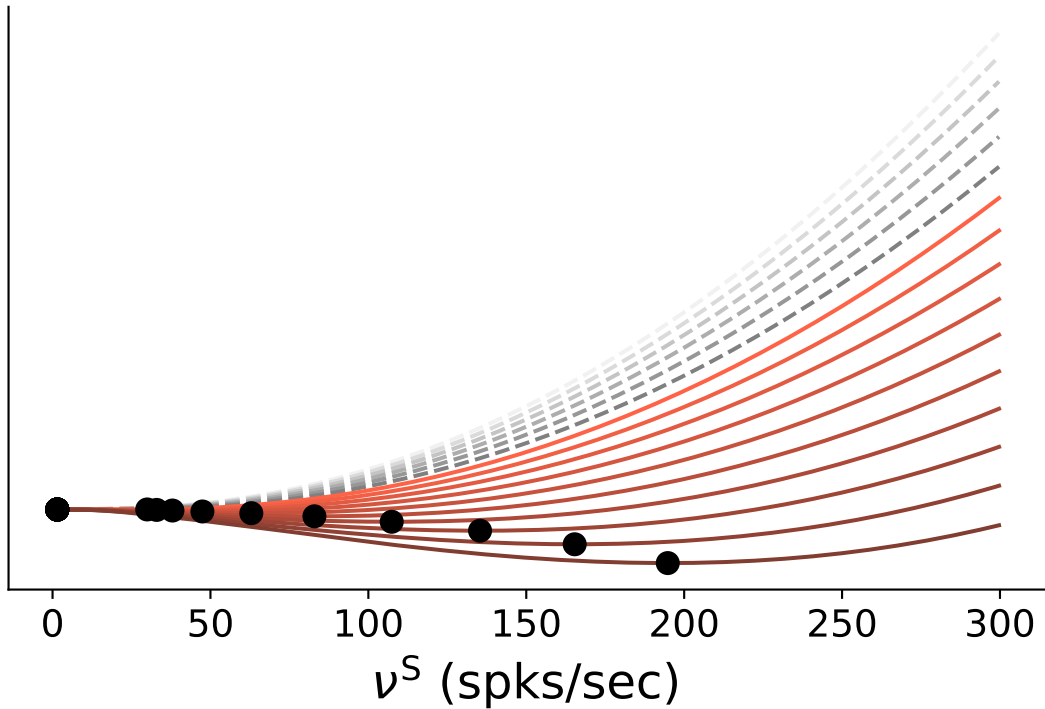

Supplement: Figure 7—source data 1. [file elife-77009-fig7-data1.zip › figure7/plots/fig7_c.pdf]

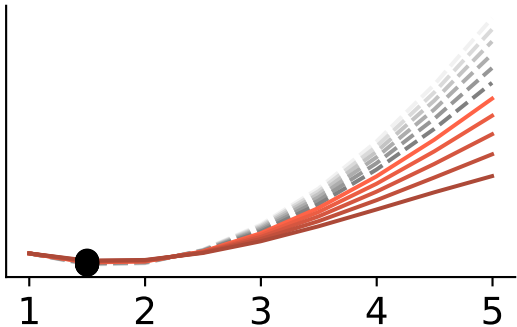

Supplement: Figure 7—source data 1. [file elife-77009-fig7-data1.zip › figure7/plots/fig7_c_zoomed.pdf]

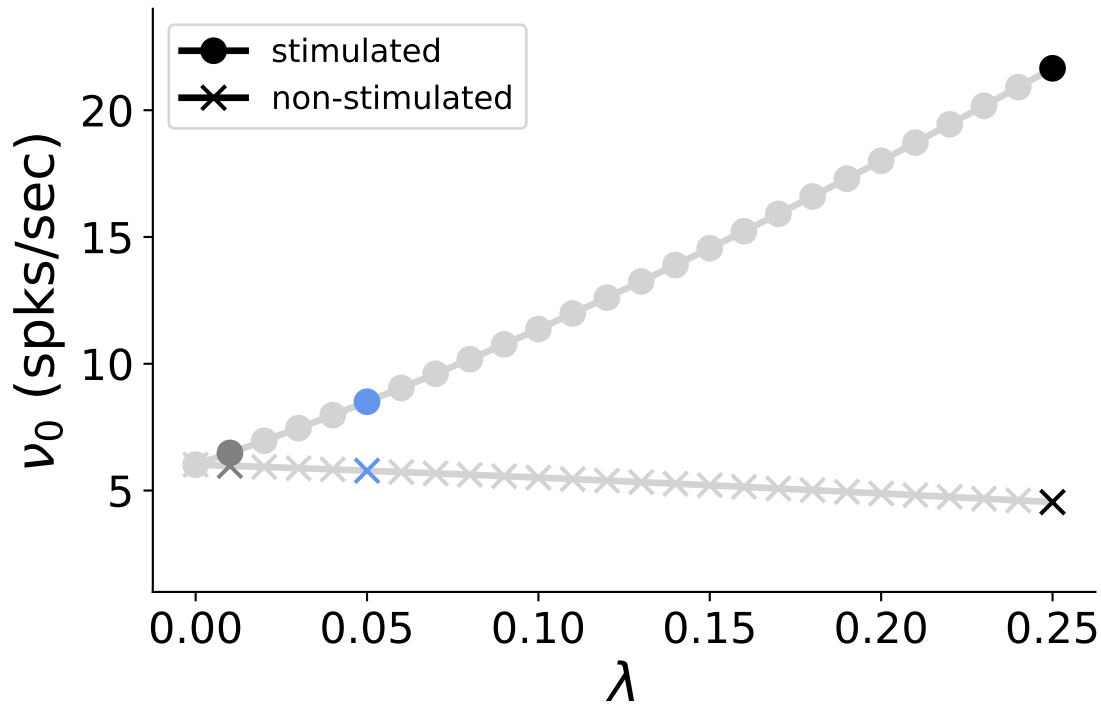

Supplement: Figure 7—source data 1. [file elife-77009-fig7-data1.zip › figure7/plots/fig7_d.pdf]

Potential

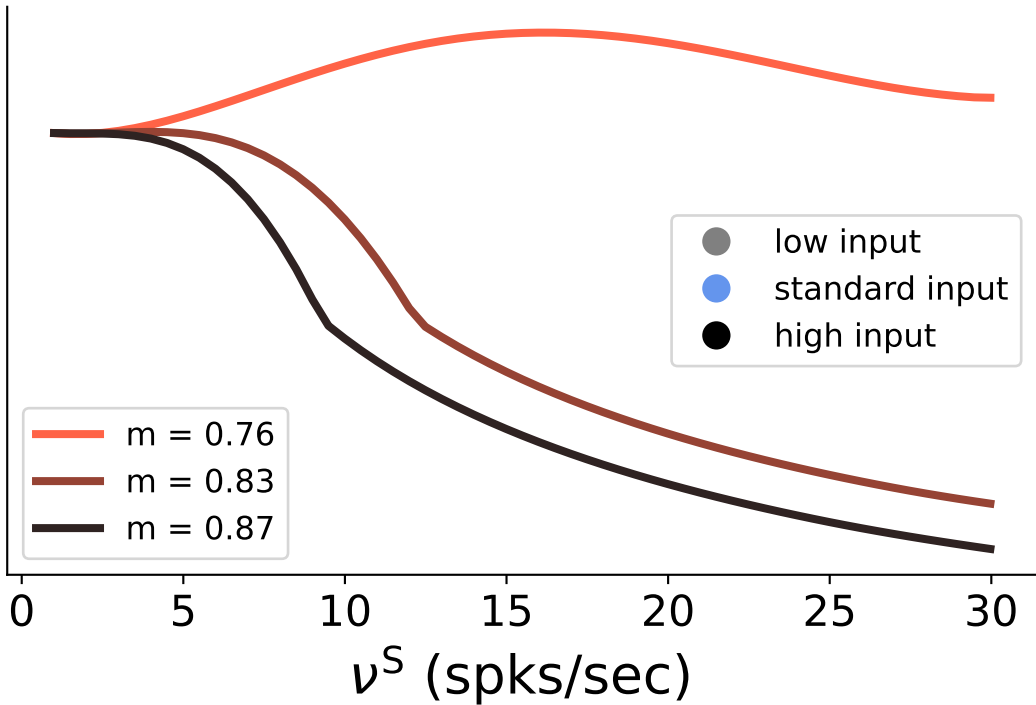

Supplement: Figure 7—source data 1. [file elife-77009-fig7-data1.zip › figure7/plots/fig7_e.pdf]

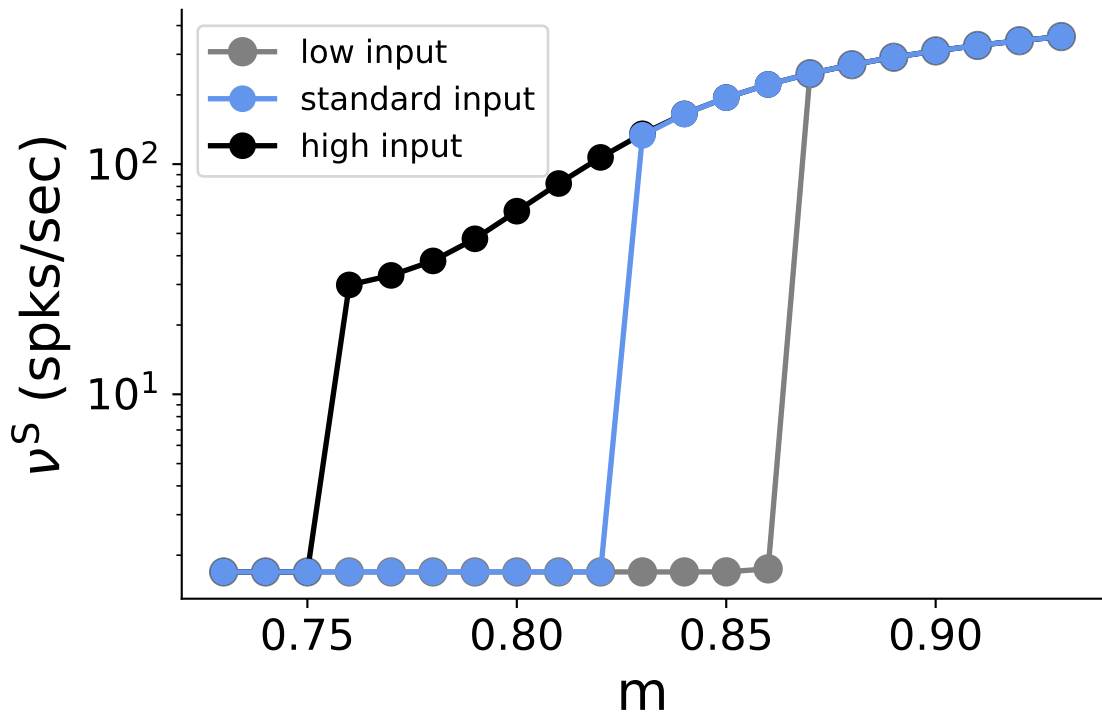

Supplement: Figure 7—source data 1. [file elife-77009-fig7-data1.zip › figure7/plots/fig7_f.pdf]

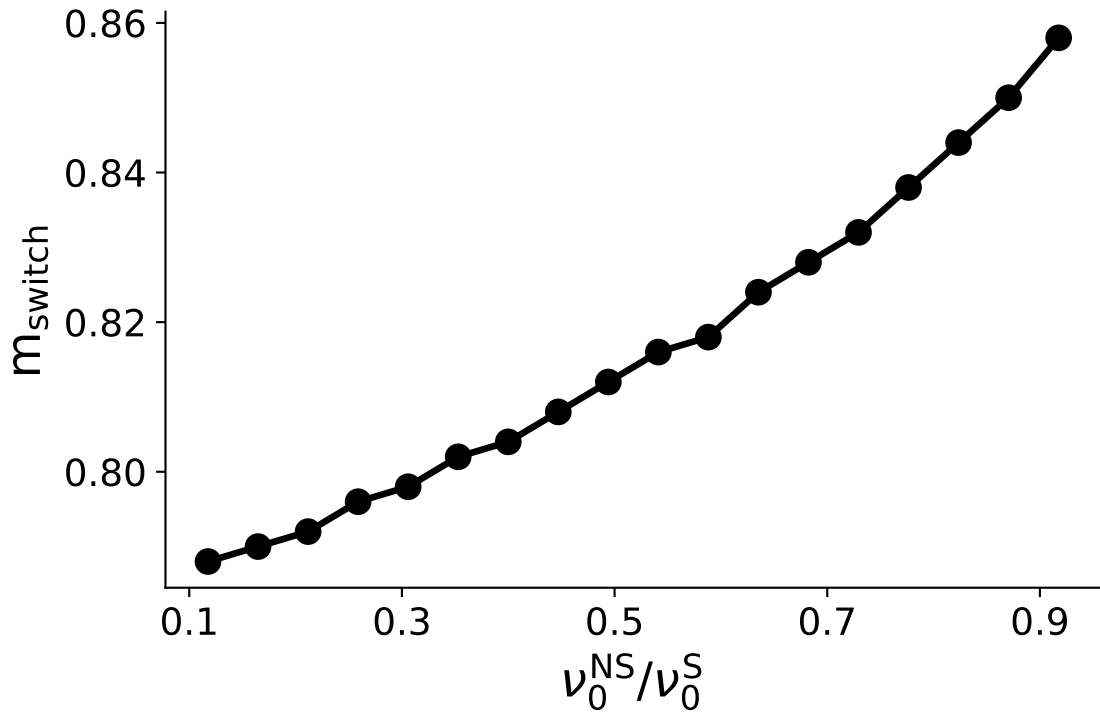

Supplement: Figure 7—source data 1. [file elife-77009-fig7-data1.zip › figure7/plots/fig7_g.pdf]

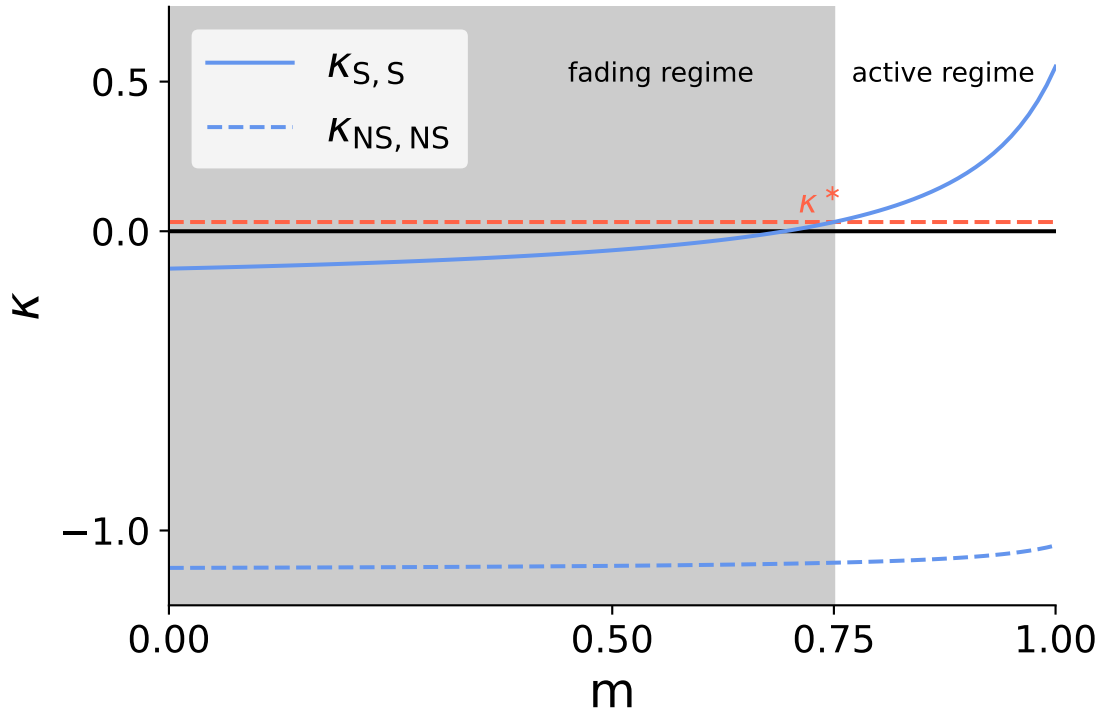

Supplement: Figure 7—source data 1. [file elife-77009-fig7-data1.zip › figure7/plots/fig7_s1_a.pdf]

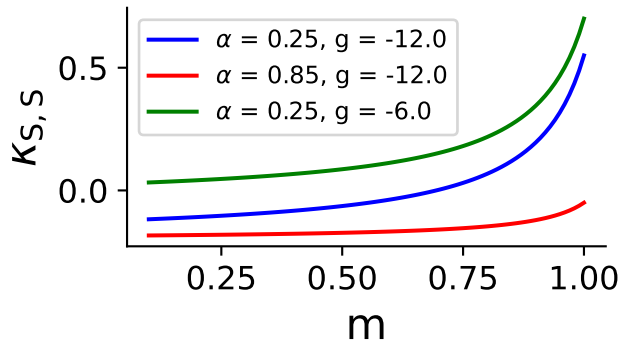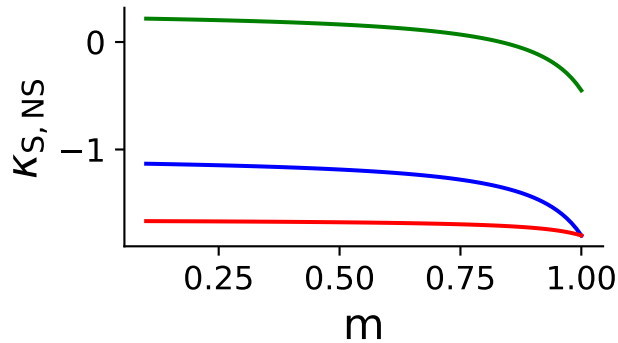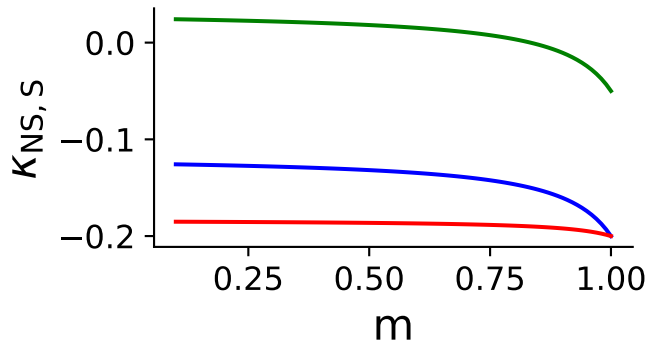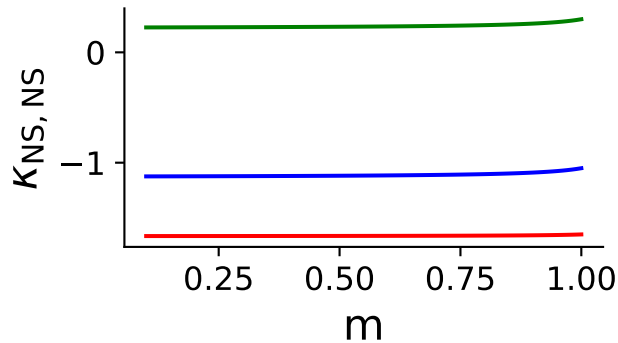

Supplement: Figure 7—source data 1. [file elife-77009-fig7-data1.zip › figure7/plots/fig7_s1_b.pdf]

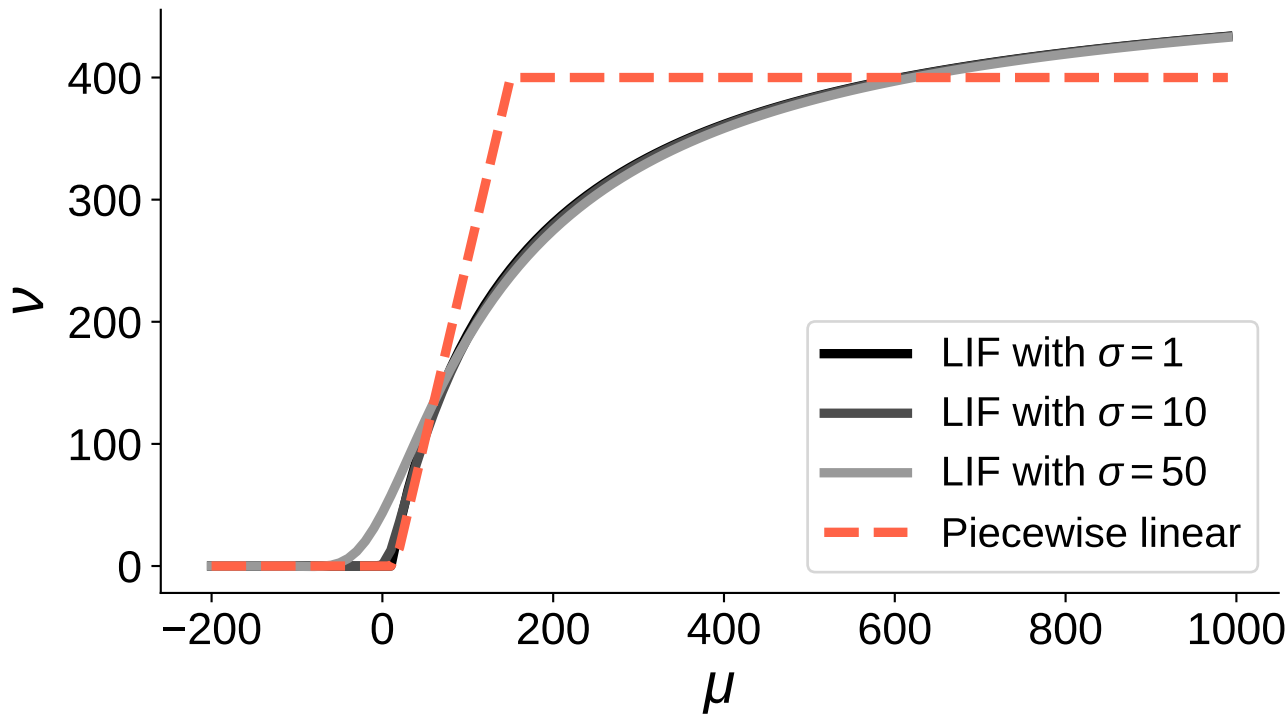

Supplement: Figure 8—source data 1. [file elife-77009-fig8-data1.zip › figure8/plots/fig8_a.pdf]

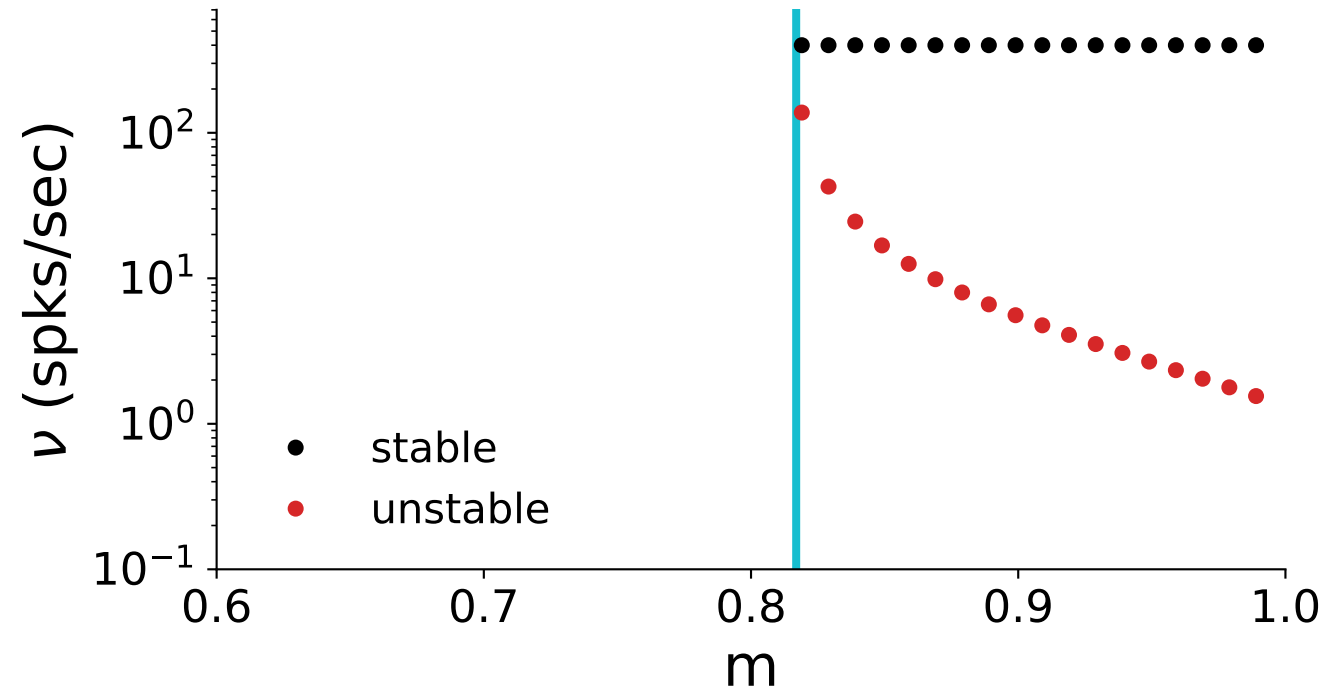

Supplement: Figure 8—source data 1. [file elife-77009-fig8-data1.zip › figure8/plots/fig8_b.pdf]

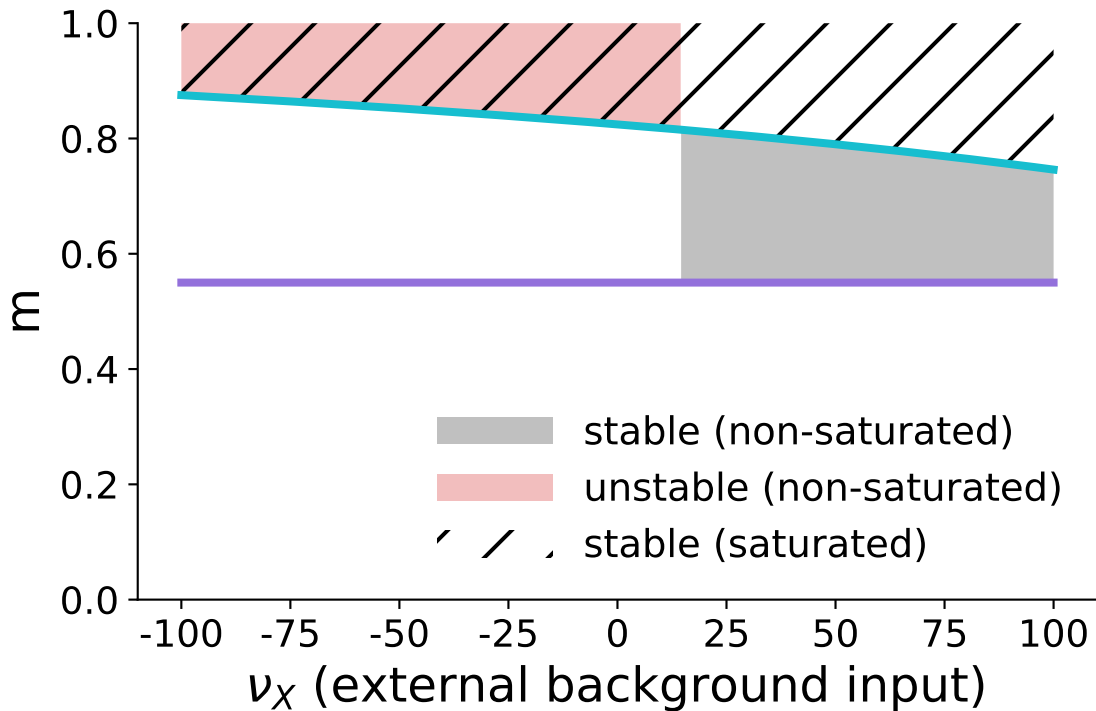

Supplement: Figure 8—source data 1. [file elife-77009-fig8-data1.zip › figure8/plots/fig8_c.pdf]

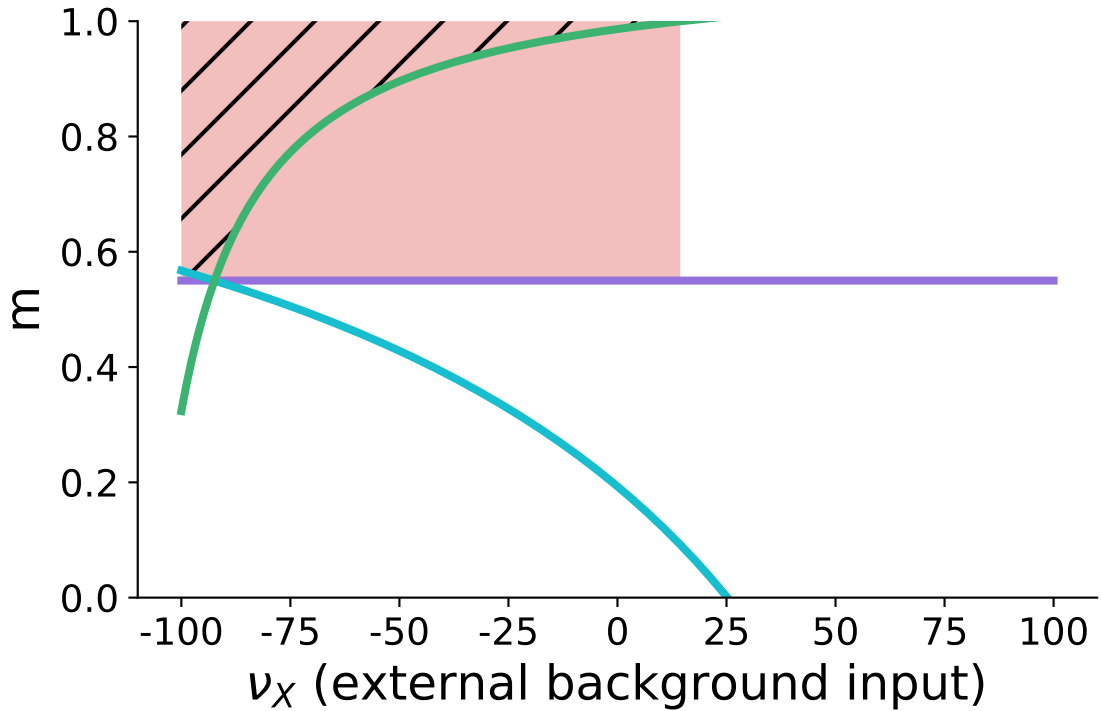

Supplement: Figure 8—source data 1. [file elife-77009-fig8-data1.zip › figure8/plots/fig8_d.pdf]

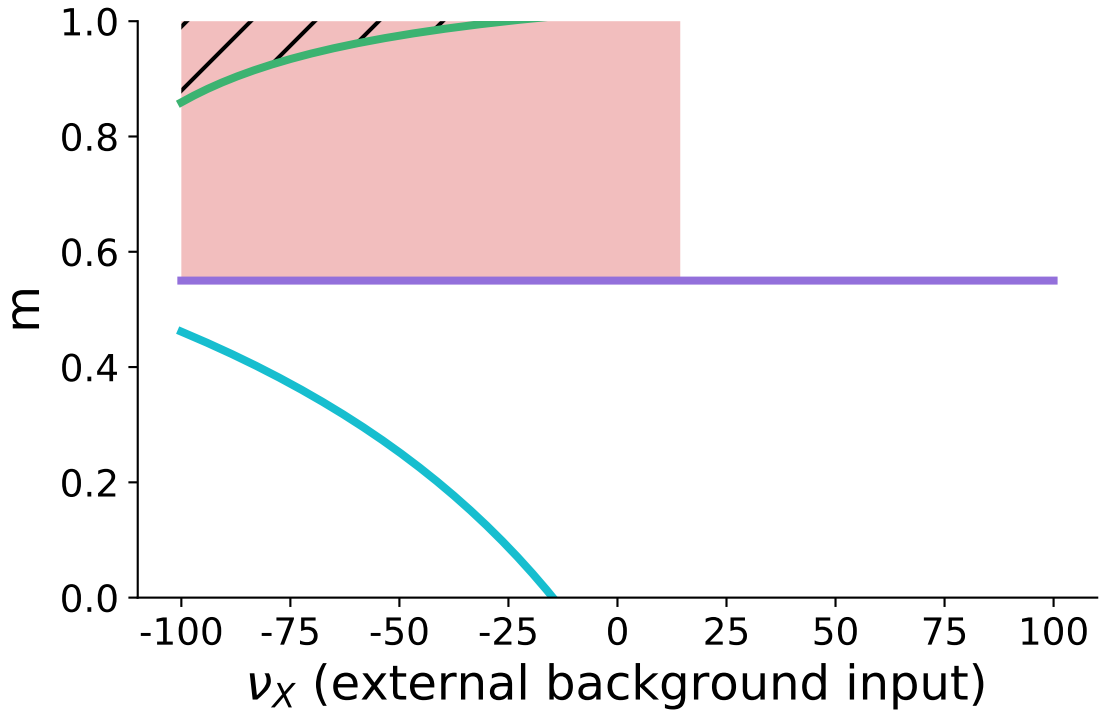

Supplement: Figure 8—source data 1. [file elife-77009-fig8-data1.zip › figure8/plots/fig8_e.pdf]

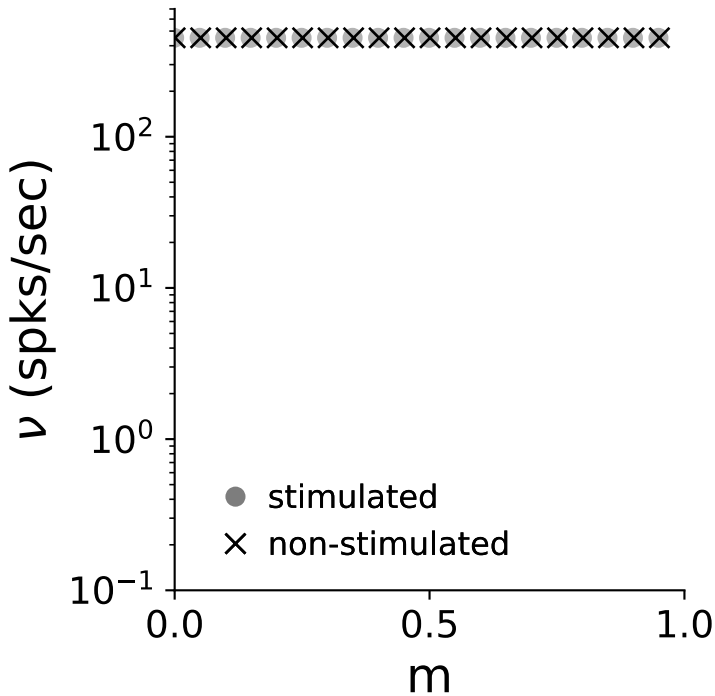

Supplement: Figure 8—source data 1. [file elife-77009-fig8-data1.zip › figure8/plots/fig8_f.pdf]

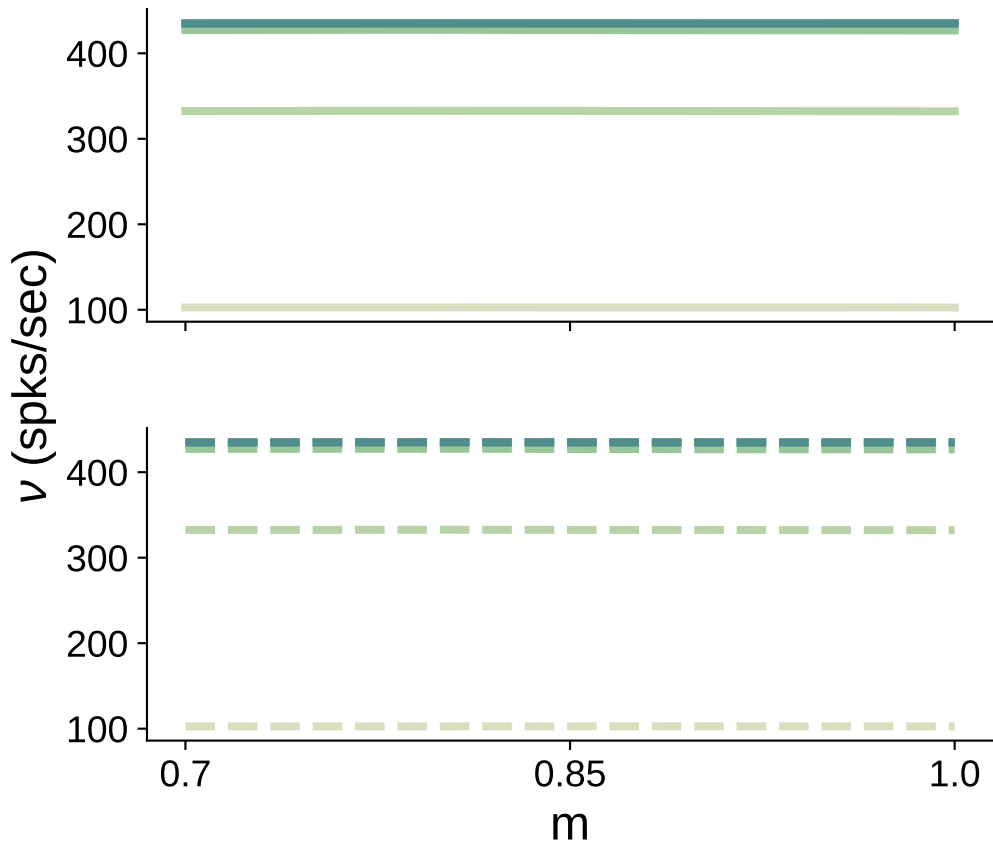

Supplement: Figure 8—source data 1. [file elife-77009-fig8-data1.zip › figure8/plots/fig8_g.pdf]

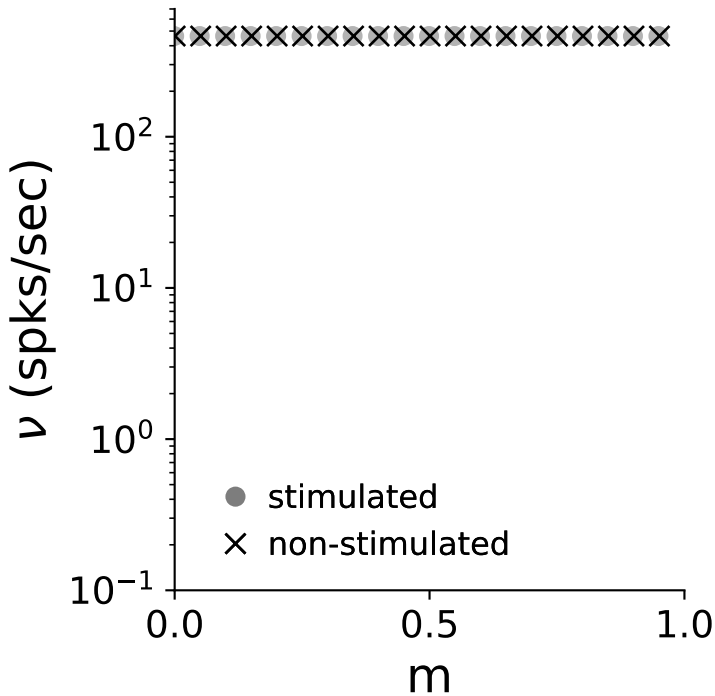

Supplement: Figure 8—source data 1. [file elife-77009-fig8-data1.zip › figure8/plots/fig8_h.pdf]

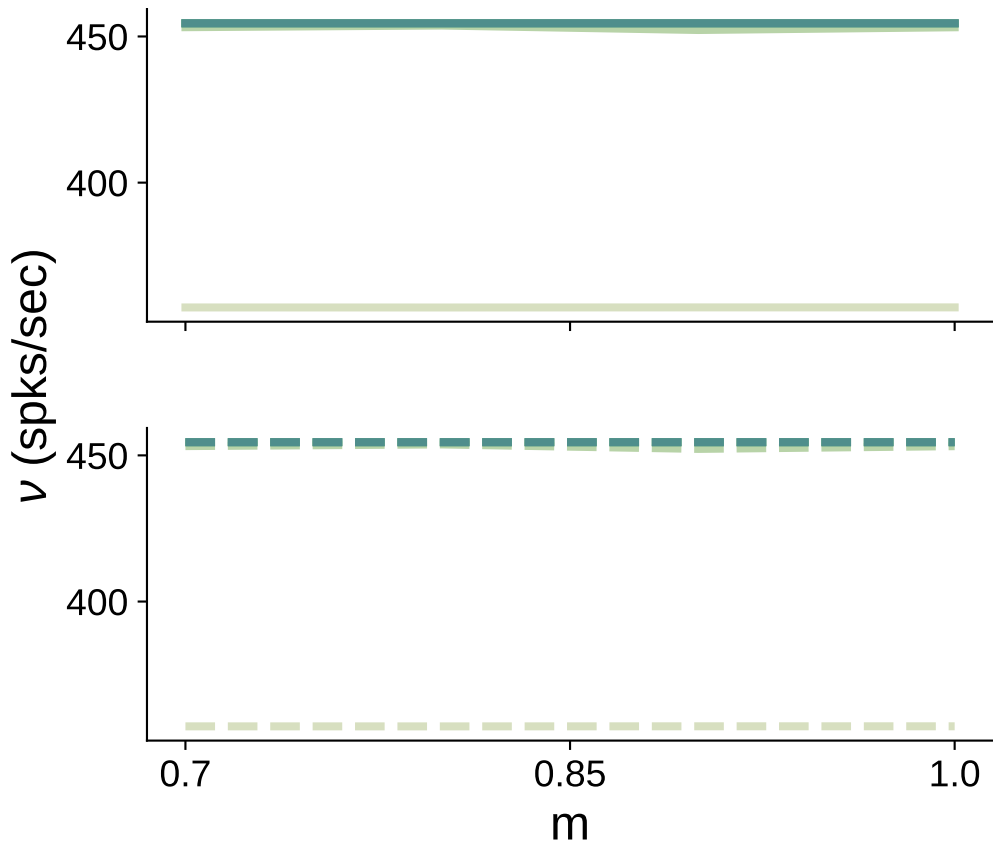

Supplement: Figure 8—source data 1. [file elife-77009-fig8-data1.zip › figure8/plots/fig8_i.pdf]

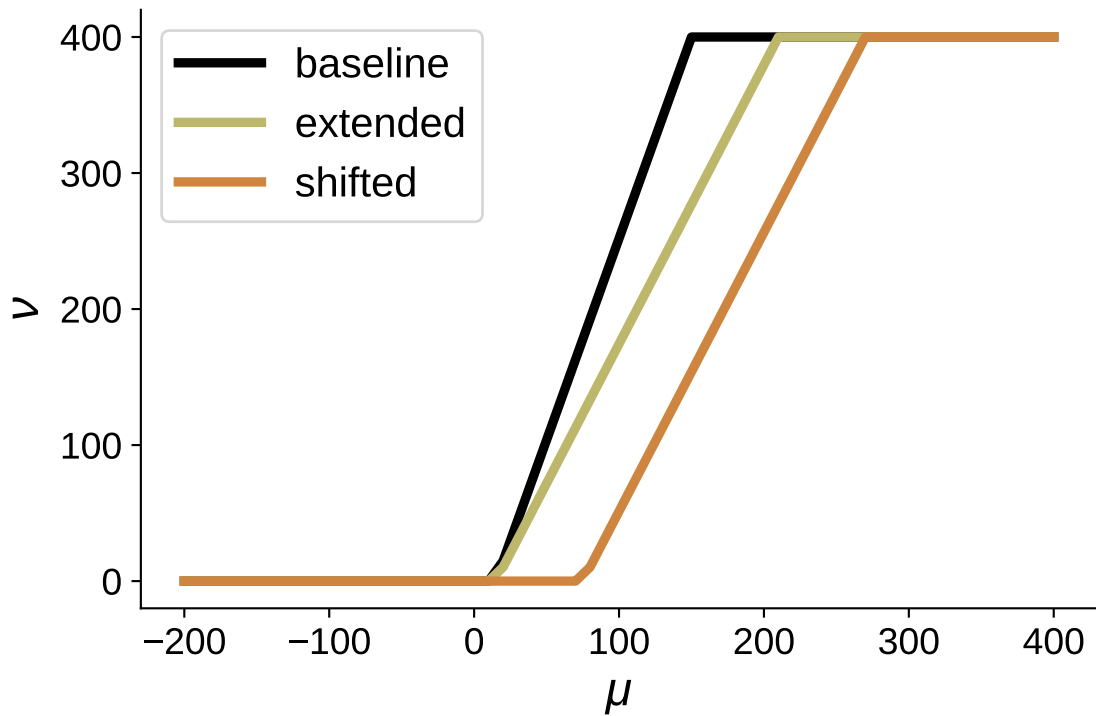

Supplement: Figure 8—source data 1. [file elife-77009-fig8-data1.zip › figure8/plots/fig8_s1_a.pdf]

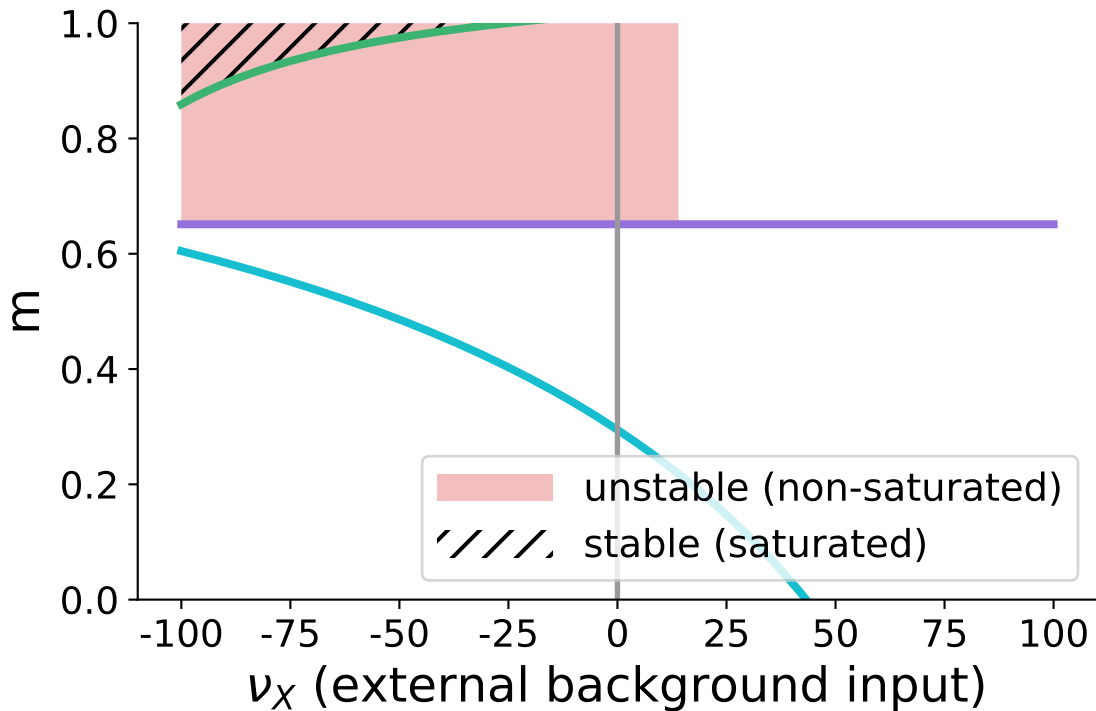

Supplement: Figure 8—source data 1. [file elife-77009-fig8-data1.zip › figure8/plots/fig8_s1_b.pdf]

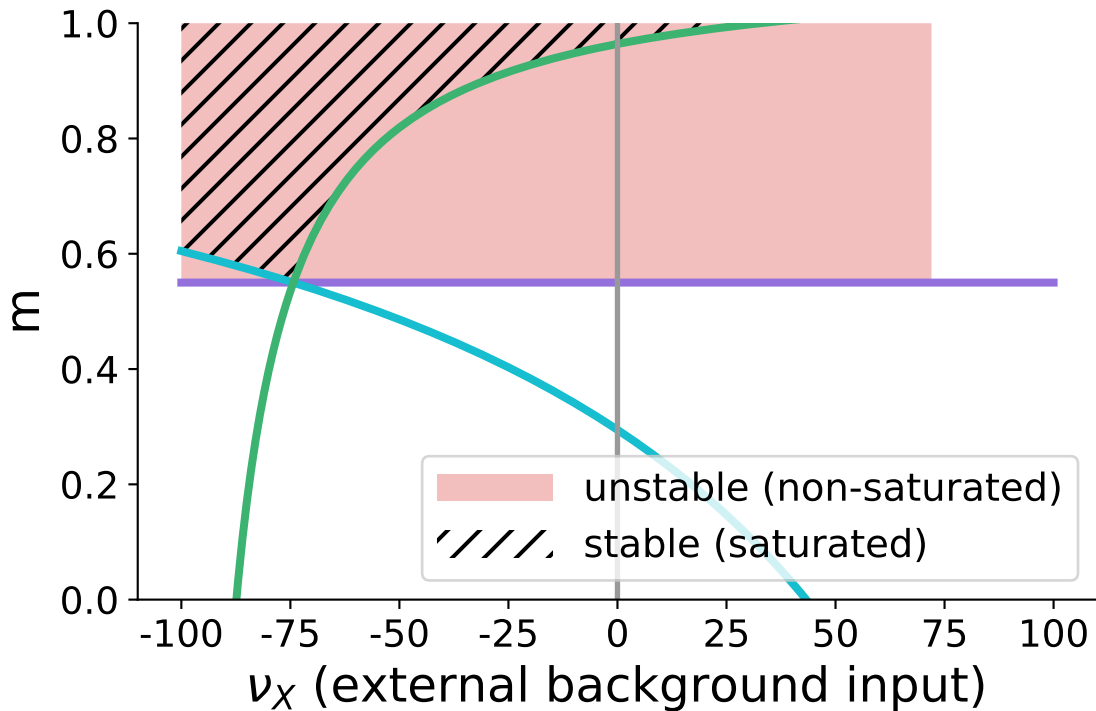

Supplement: Figure 8—source data 1. [file elife-77009-fig8-data1.zip › figure8/plots/fig8_s1_c.pdf]

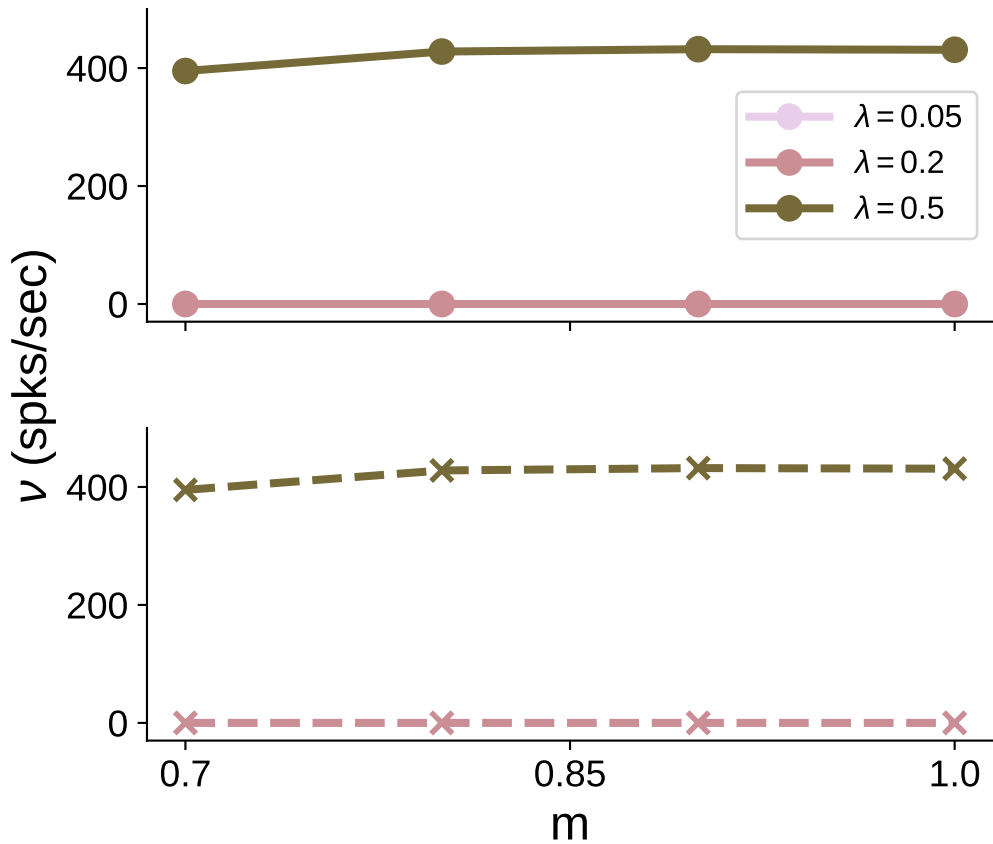

Supplement: Figure 8—source data 1. [file elife-77009-fig8-data1.zip › figure8/plots/fig8_s2_a.pdf]

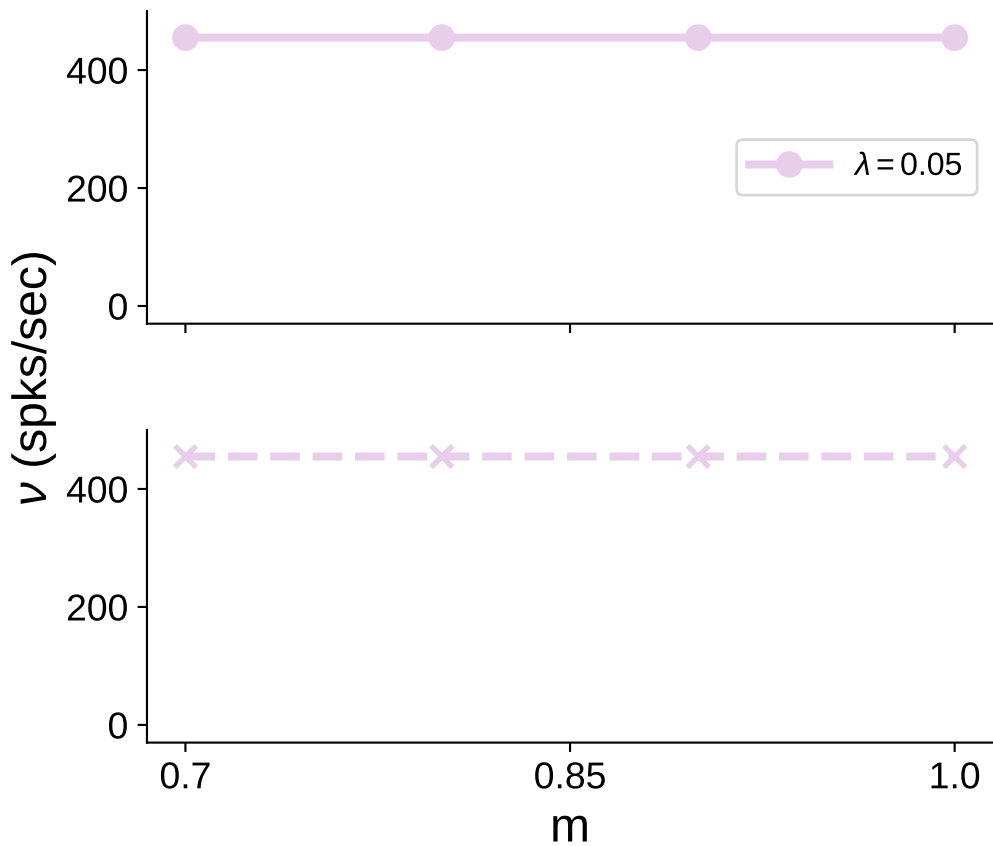

Supplement: Figure 8—source data 1. [file elife-77009-fig8-data1.zip › figure8/plots/fig8_s2_b.pdf]

Rate  $v_5$

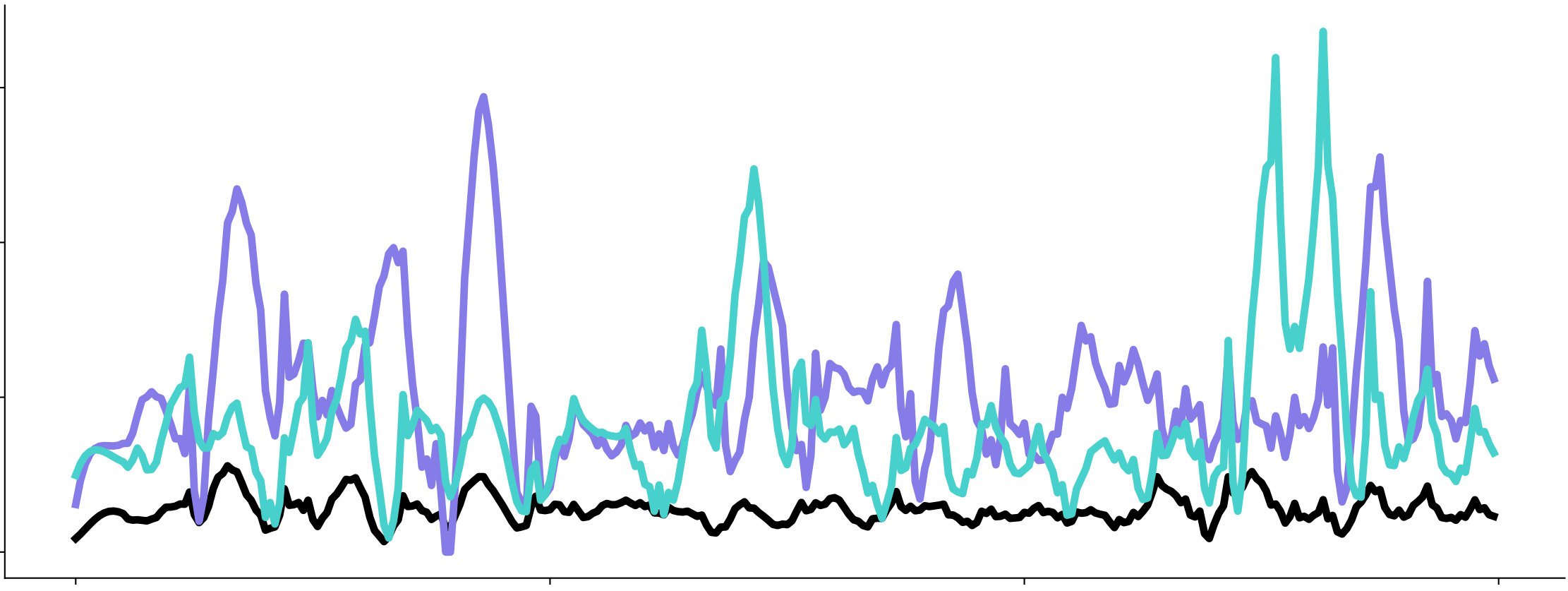

Supplement: Figure 9—source data 1. [file elife-77009-fig9-data1.zip › figure9/plots/fig9_b_coex.pdf]

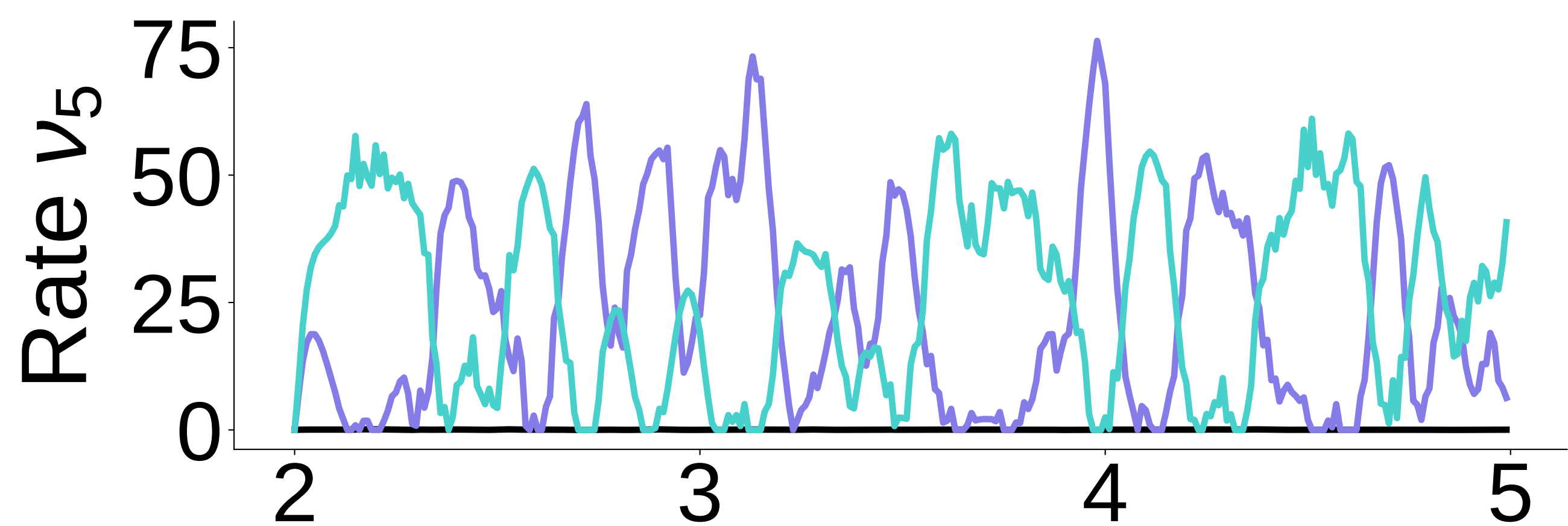

Supplement: Figure 9—source data 1. [file elife-77009-fig9-data1.zip › figure9/plots/fig9_b_wlc.pdf]

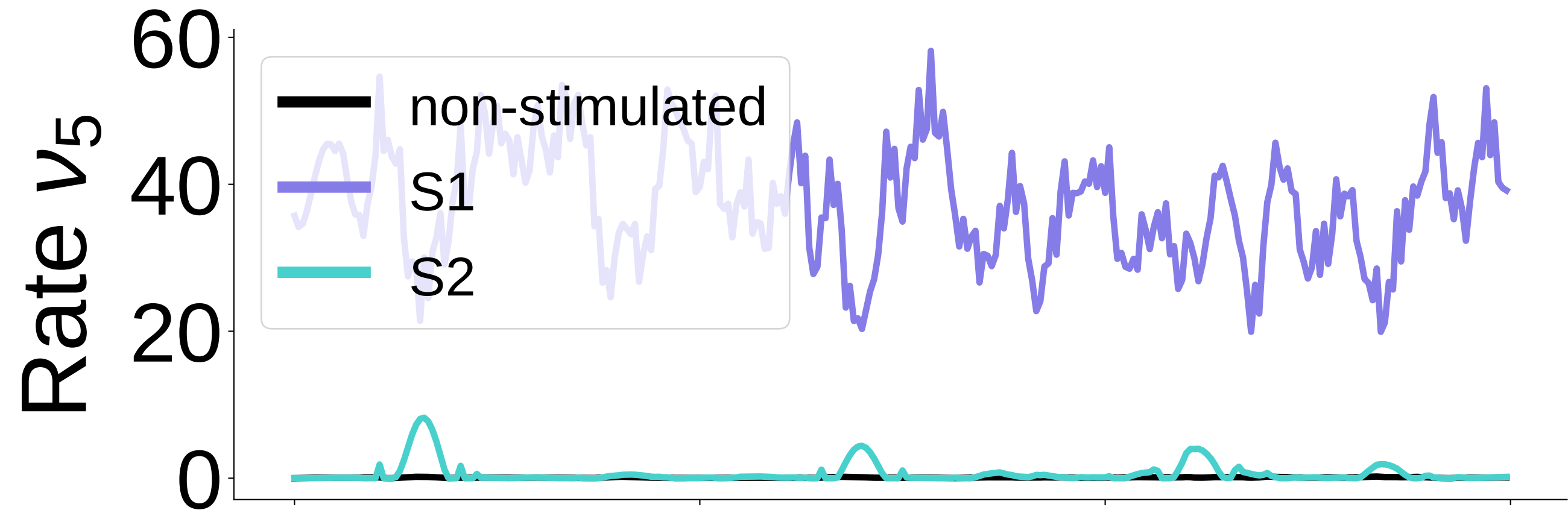

Supplement: Figure 9—source data 1. [file elife-77009-fig9-data1.zip › figure9/plots/fig9_b_wta.pdf]

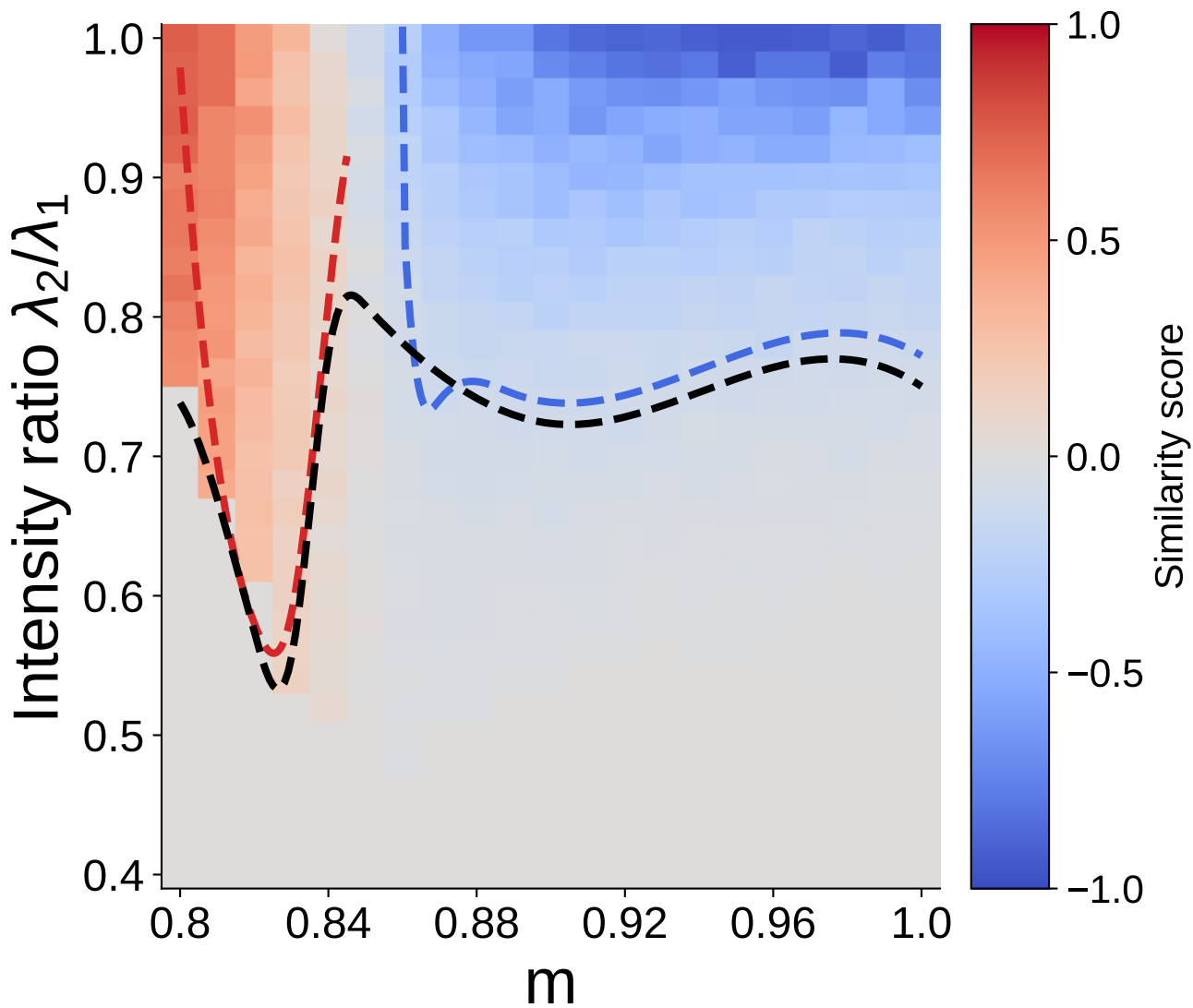

Supplement: Figure 9—source data 1. [file elife-77009-fig9-data1.zip › figure9/plots/fig9_c.pdf]

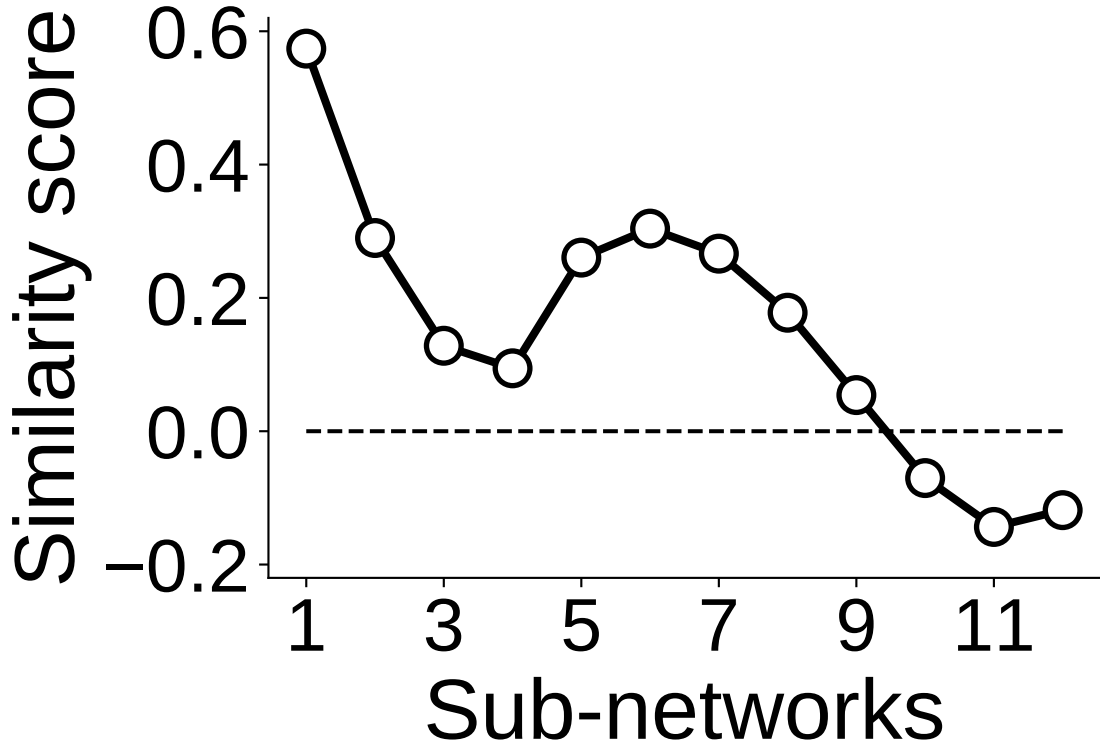

Supplement: Figure 9—source data 1. [file elife-77009-fig9-data1.zip › figure9/plots/fig9_d.pdf]

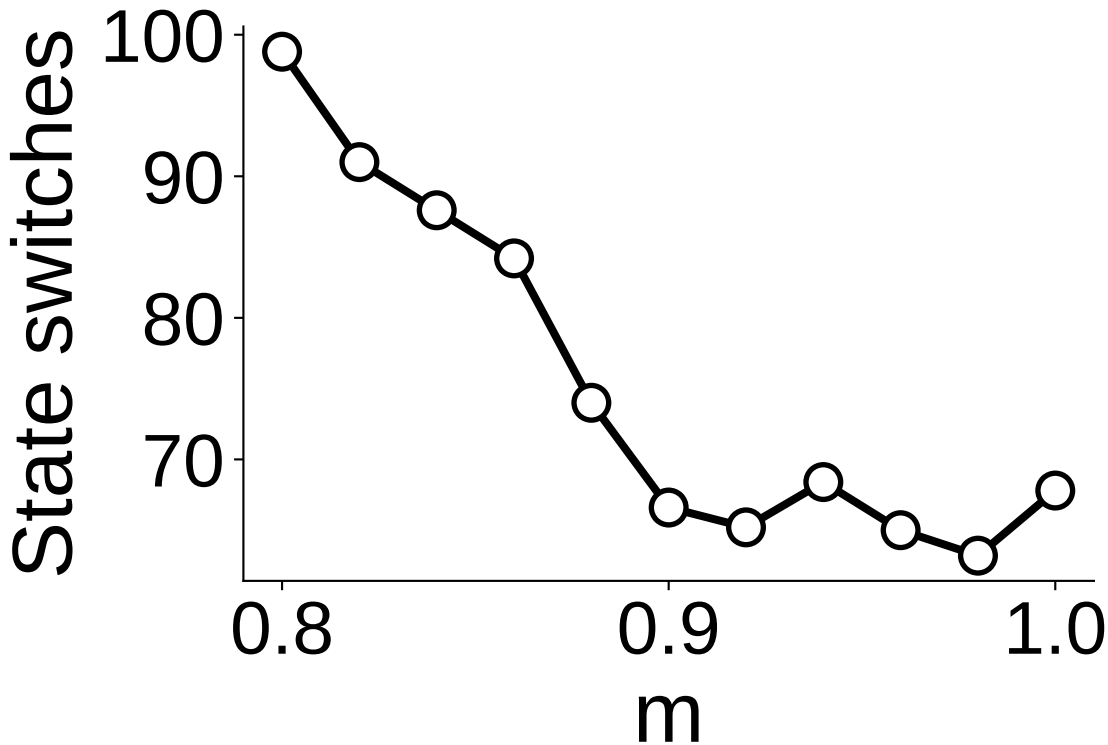

Supplement: Figure 9—source data 1. [file elife-77009-fig9-data1.zip › figure9/plots/fig9_f.pdf]

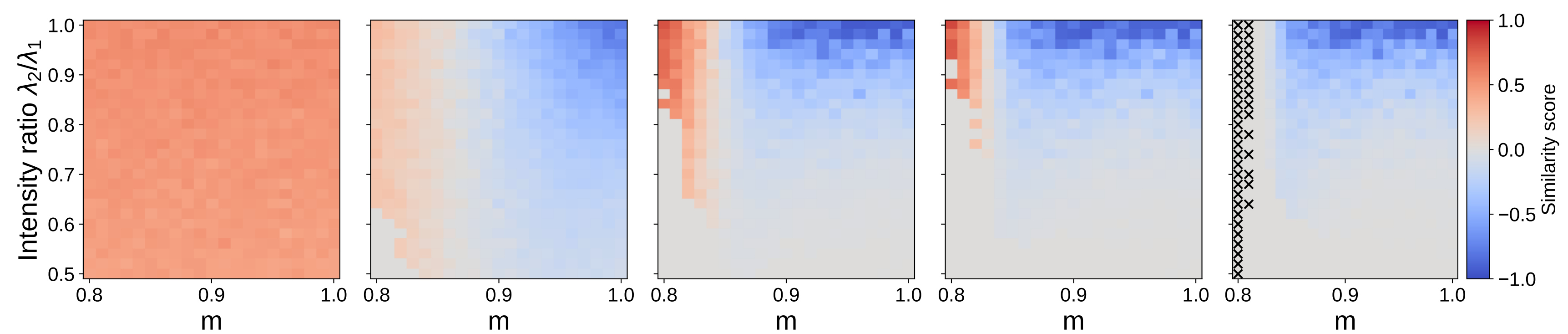

Supplement: Figure 9—source data 1. [file elife-77009-fig9-data1.zip › figure9/plots/fig9_s1.pdf]

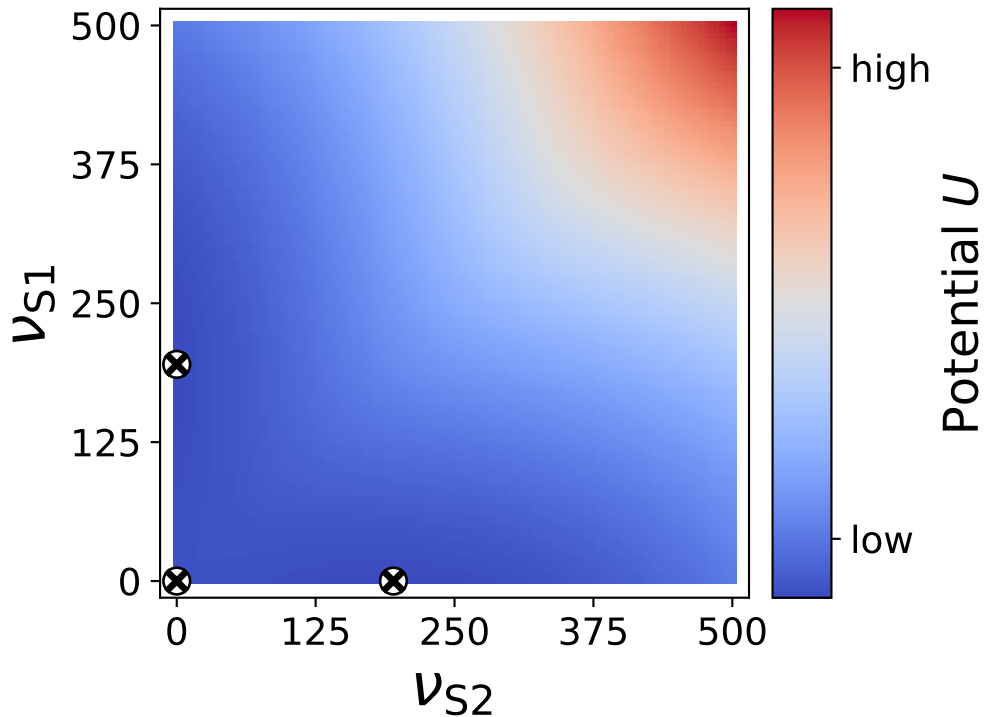

Supplement: Figure 9—source data 1. [file elife-77009-fig9-data1.zip › figure9/plots/fig9_s2_m=0.85.pdf]

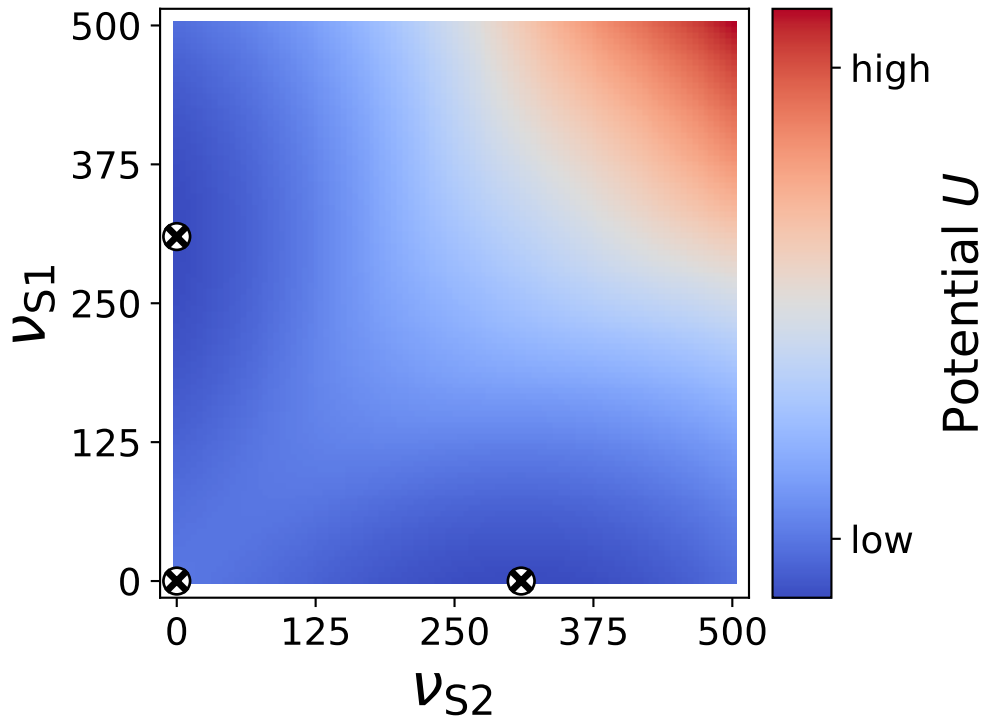

Supplement: Figure 9—source data 1. [file elife-77009-fig9-data1.zip › figure9/plots/fig9_s2_m=0.9.pdf]

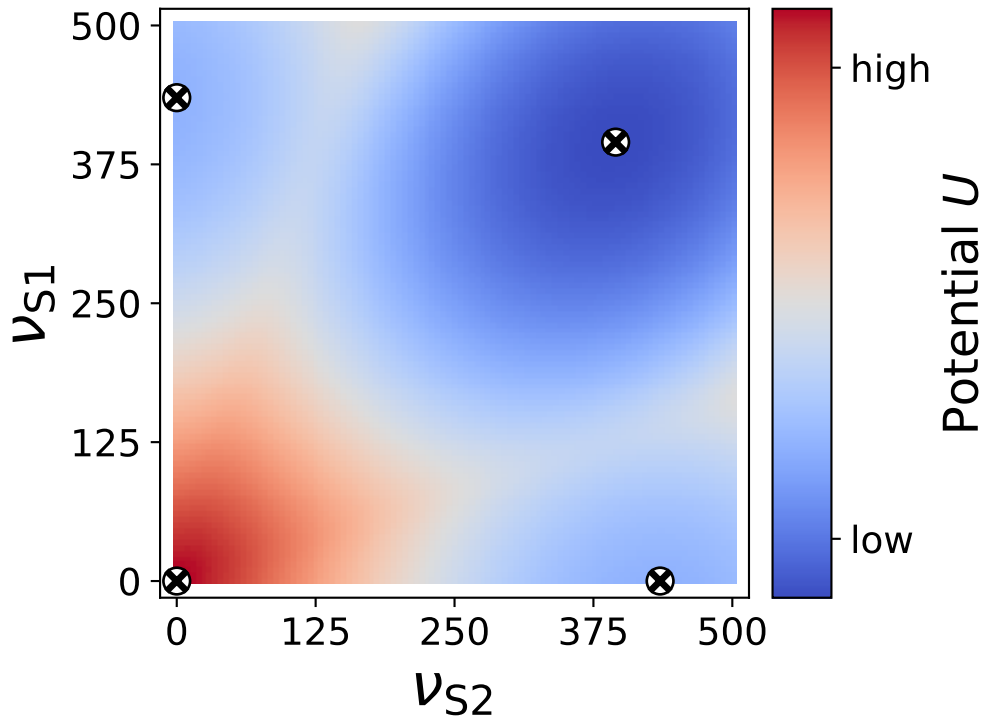

Supplement: Figure 9—source data 1. [file elife-77009-fig9-data1.zip › figure9/plots/fig9_s2_m=1.0.pdf]

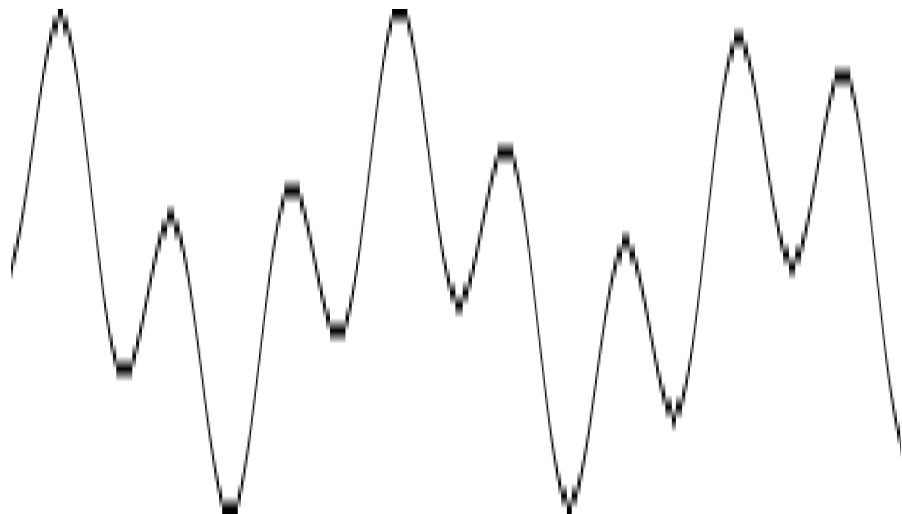

Supplement: Figure 10—source data 1. [file elife-77009-fig10-data1.zip › figure10/plots/fig10_b.pdf]

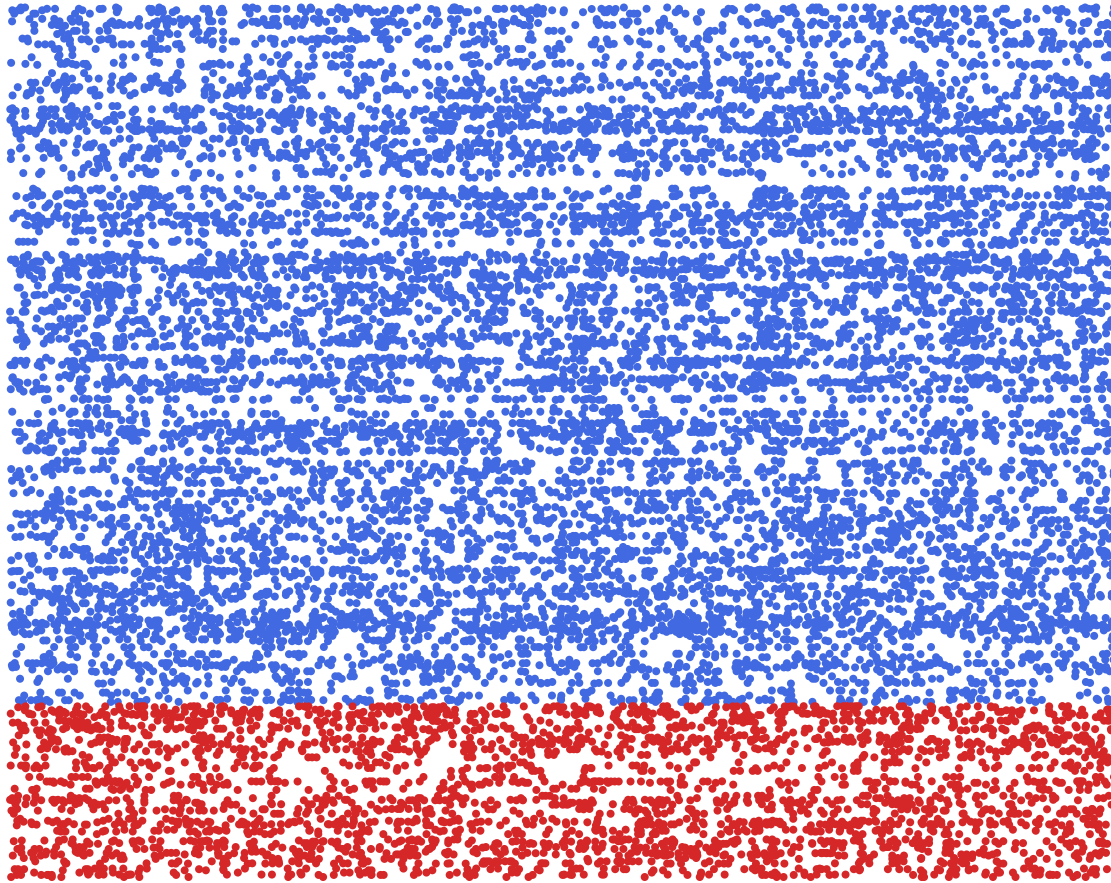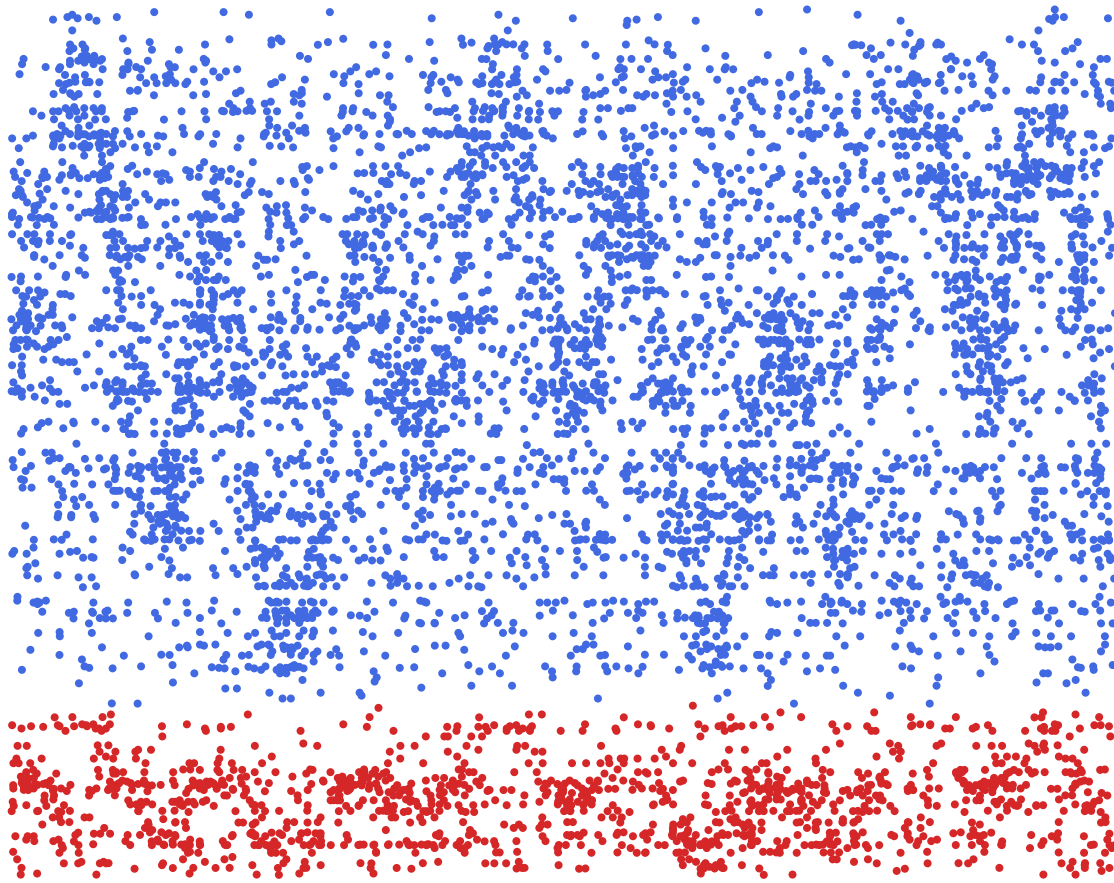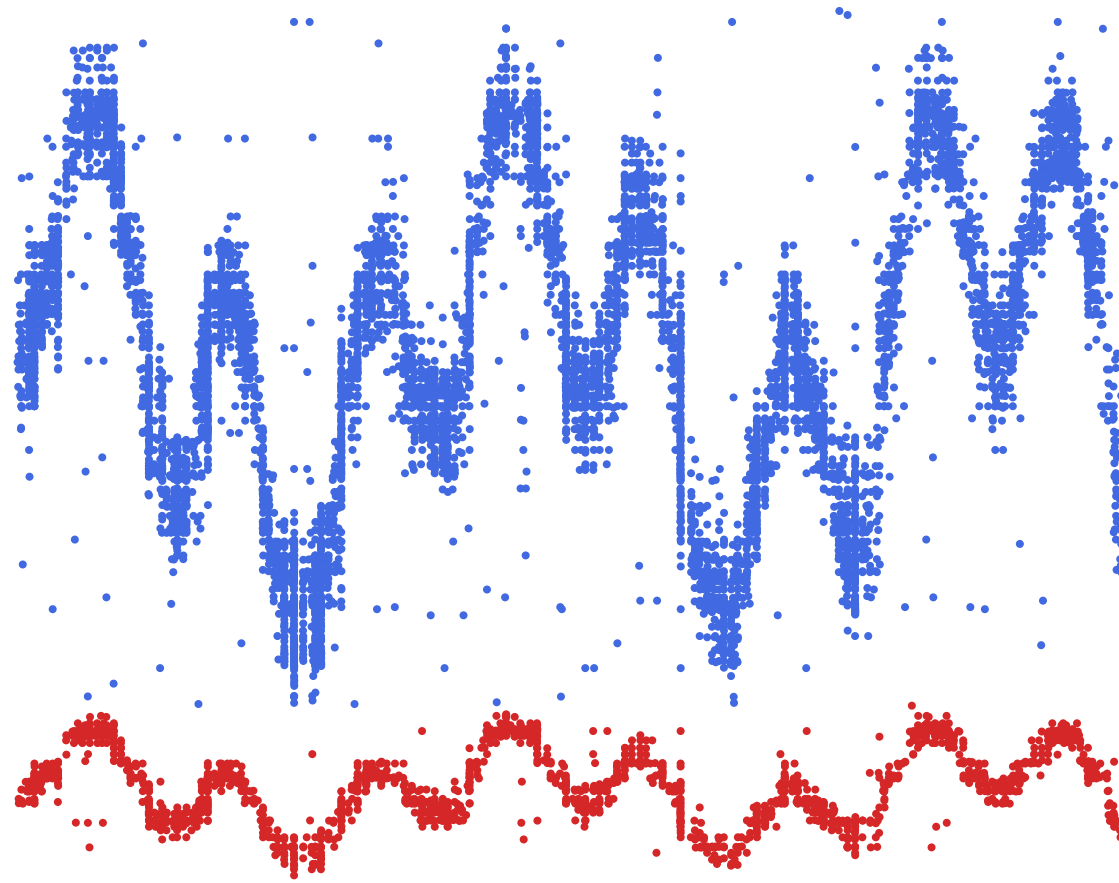

Supplement: Figure 10—source data 1. [file elife-77009-fig10-data1.zip › figure10/plots/fig10_c_raster.pdf]

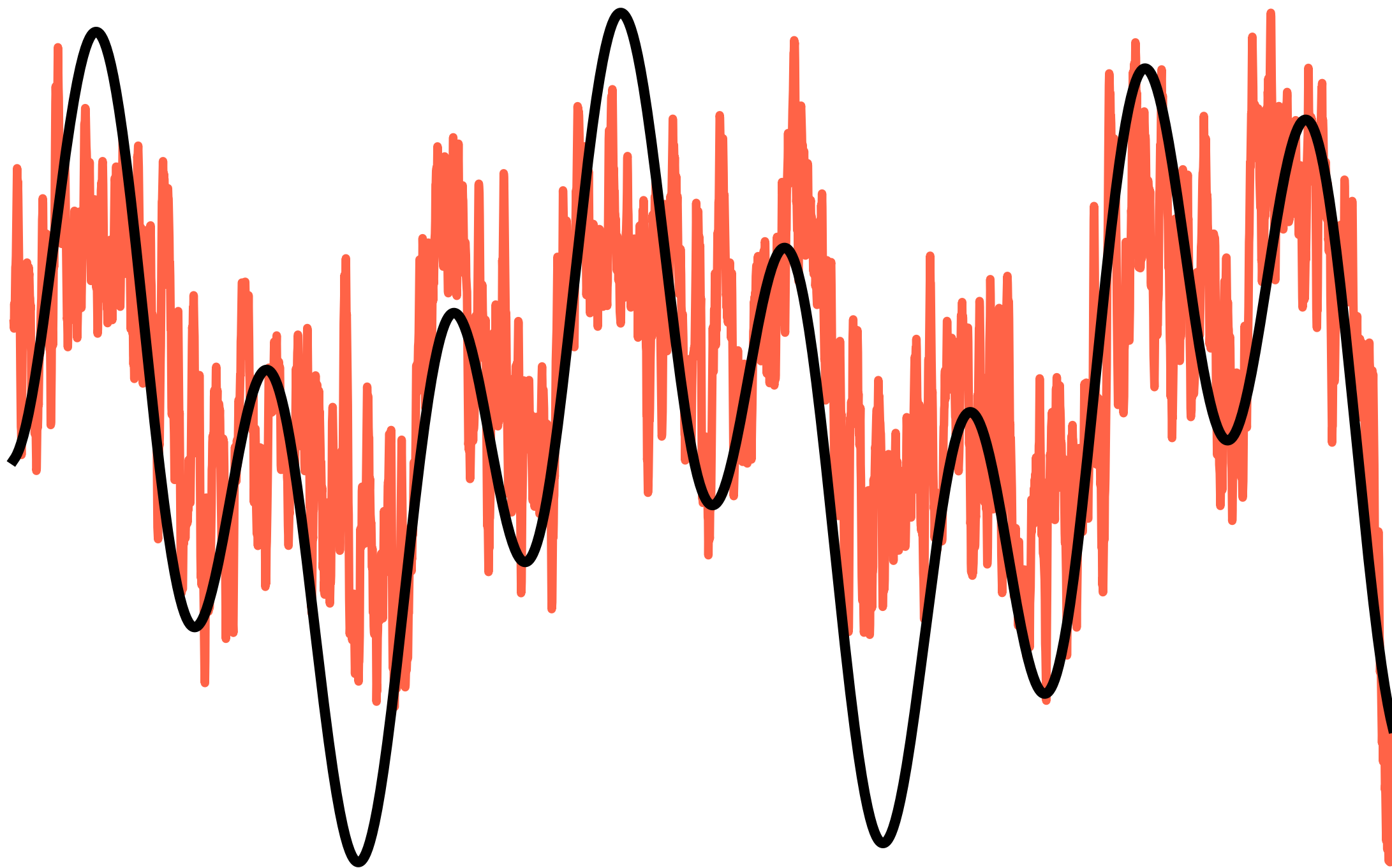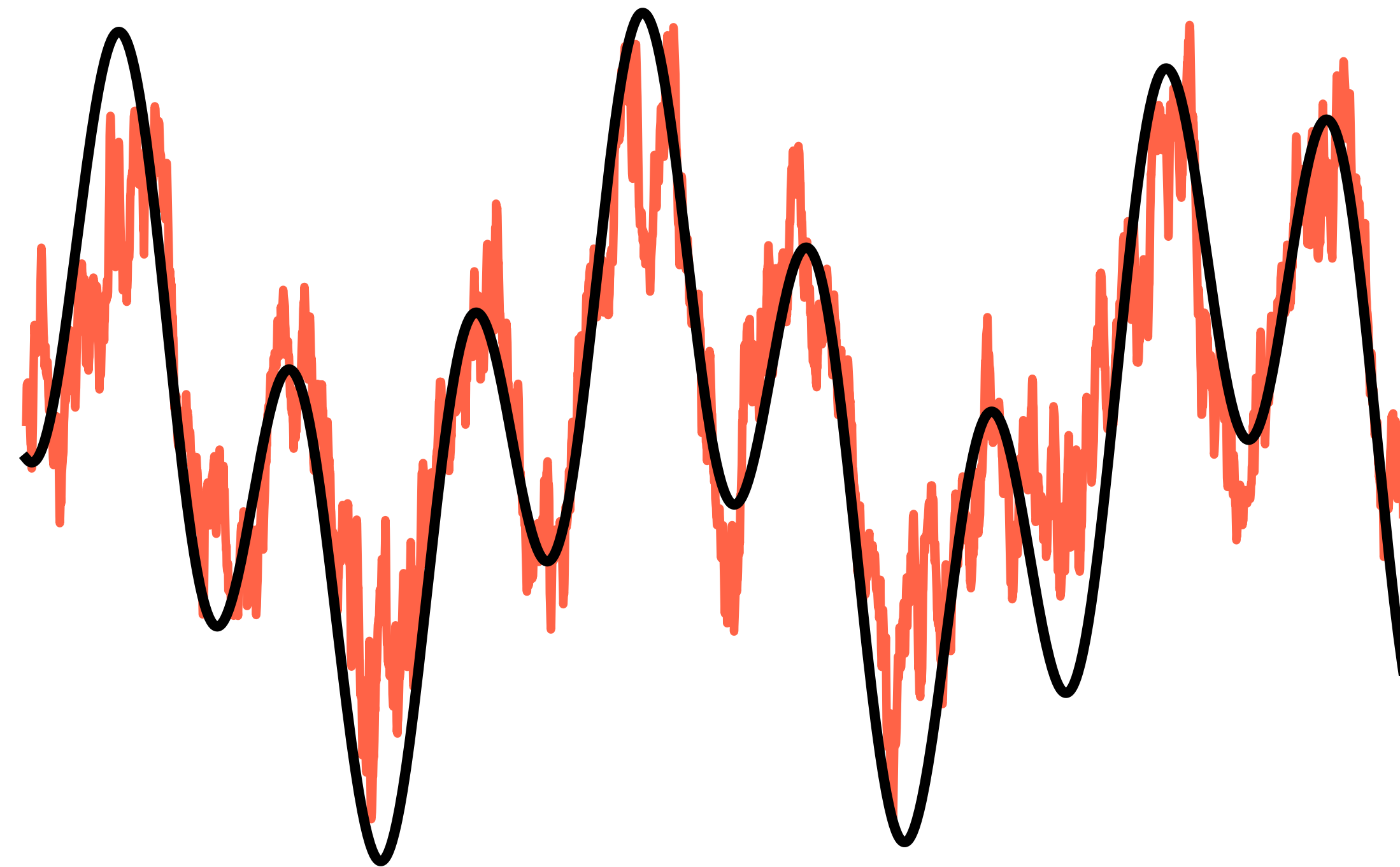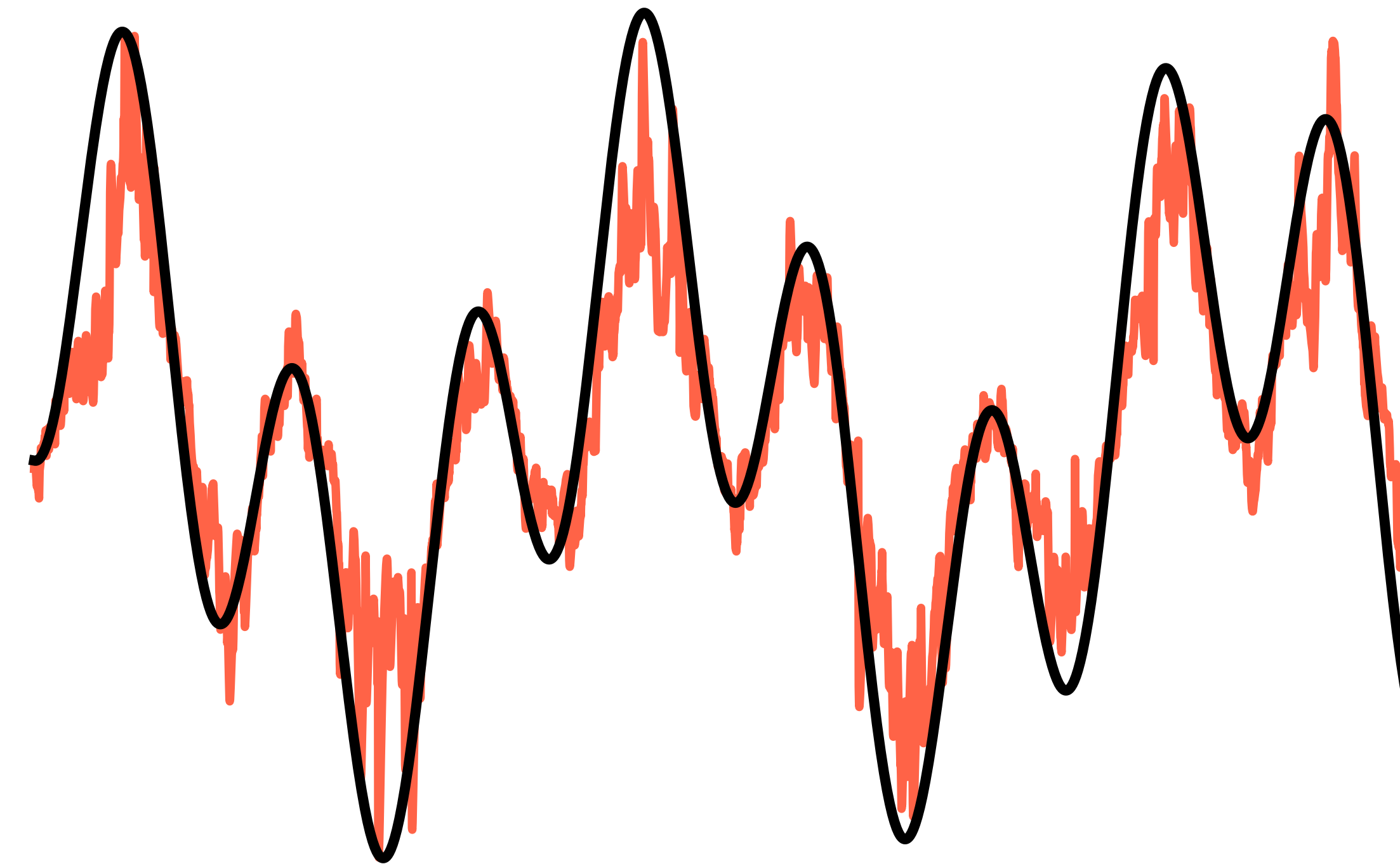

Supplement: Figure 10—source data 1. [file elife-77009-fig10-data1.zip › figure10/plots/fig10_c_readout.pdf]

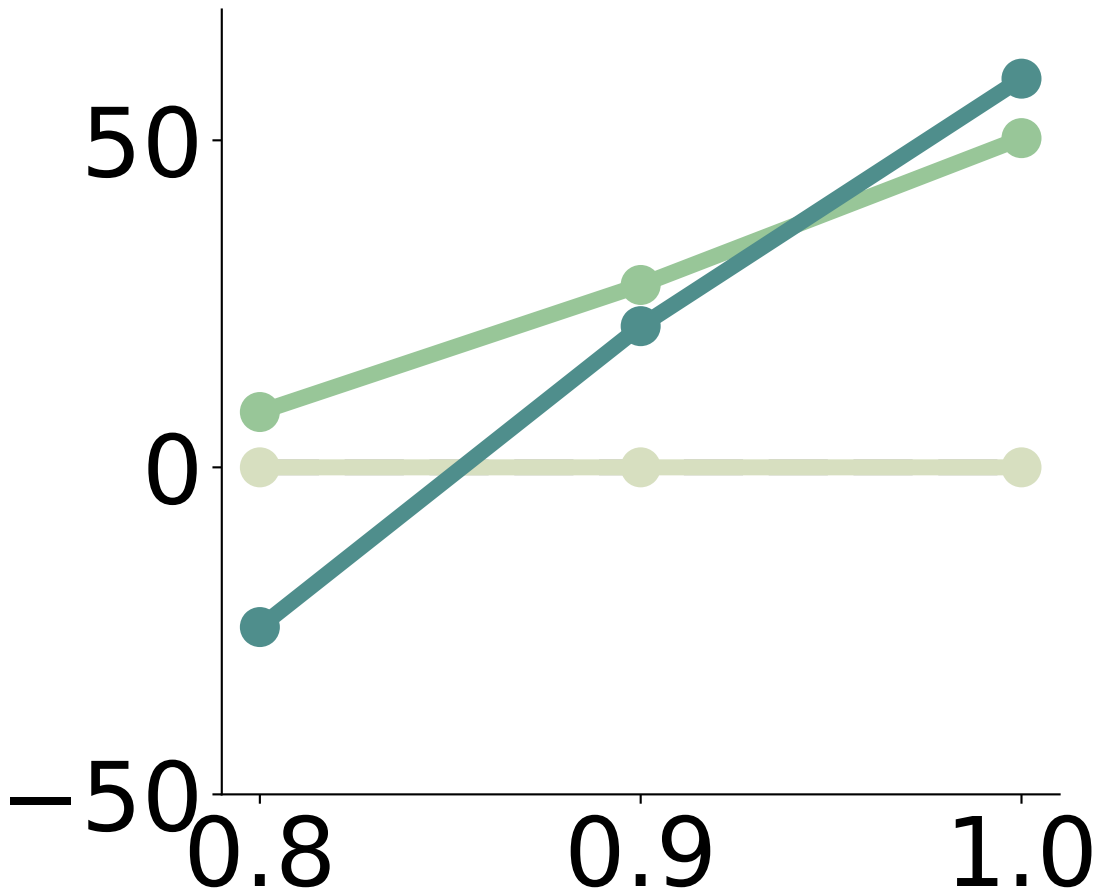

Supplement: Figure 10—source data 1. [file elife-77009-fig10-data1.zip › figure10/plots/fig10_d_perf_modules.pdf]

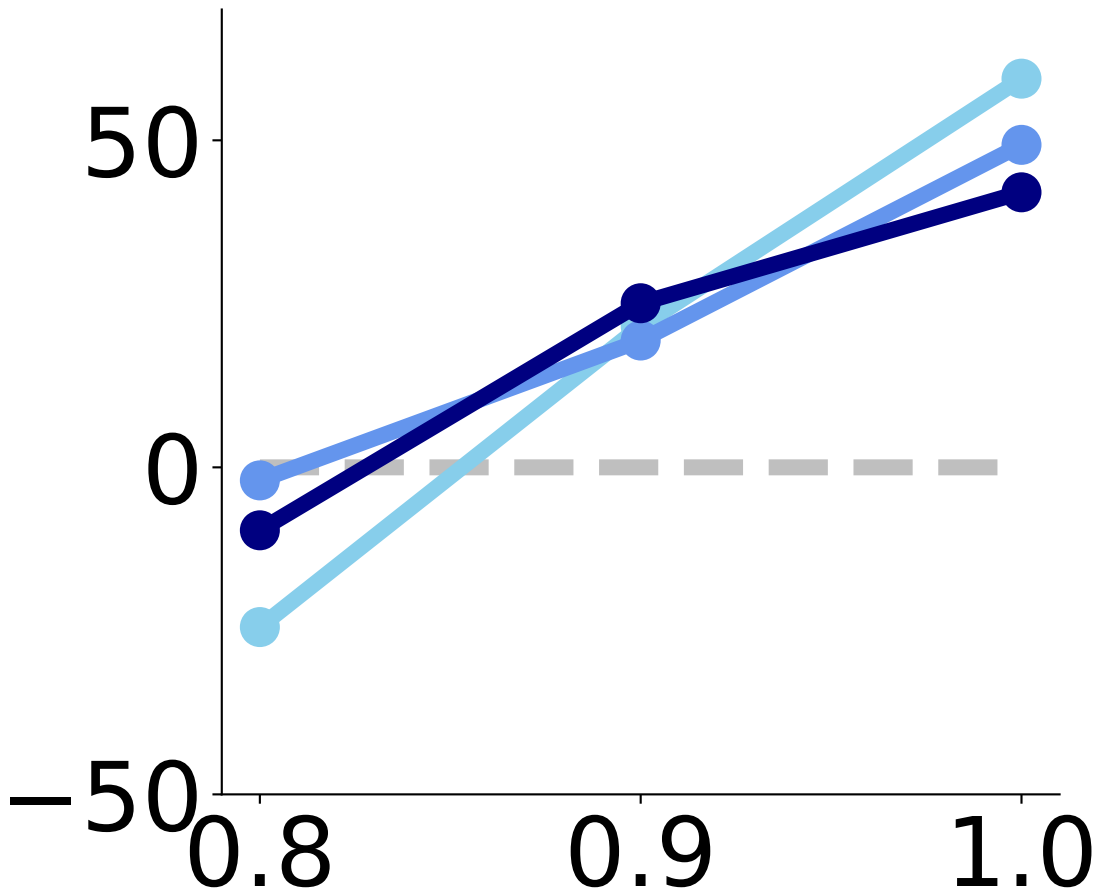

Supplement: Figure 10—source data 1. [file elife-77009-fig10-data1.zip › figure10/plots/fig10_d_perf_noise.pdf]

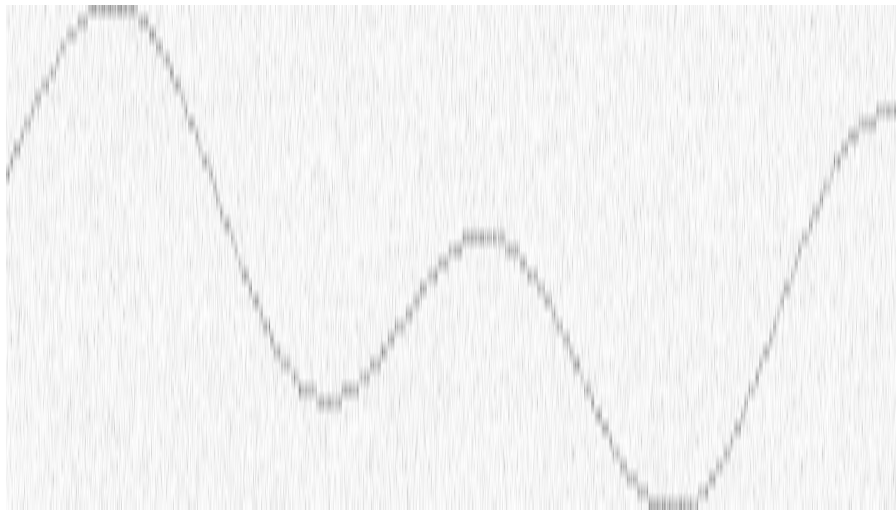

Supplement: Figure 10—source data 1. [file elife-77009-fig10-data1.zip › figure10/plots/fig10_e.pdf]

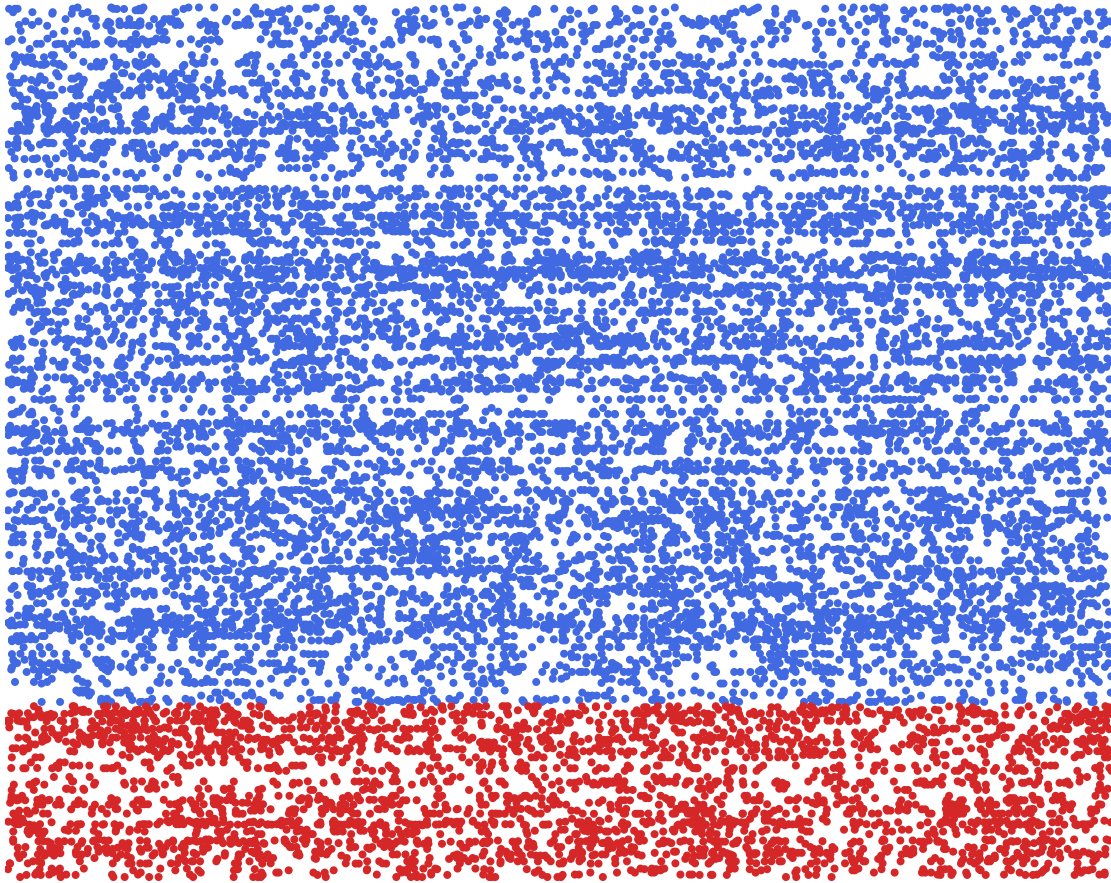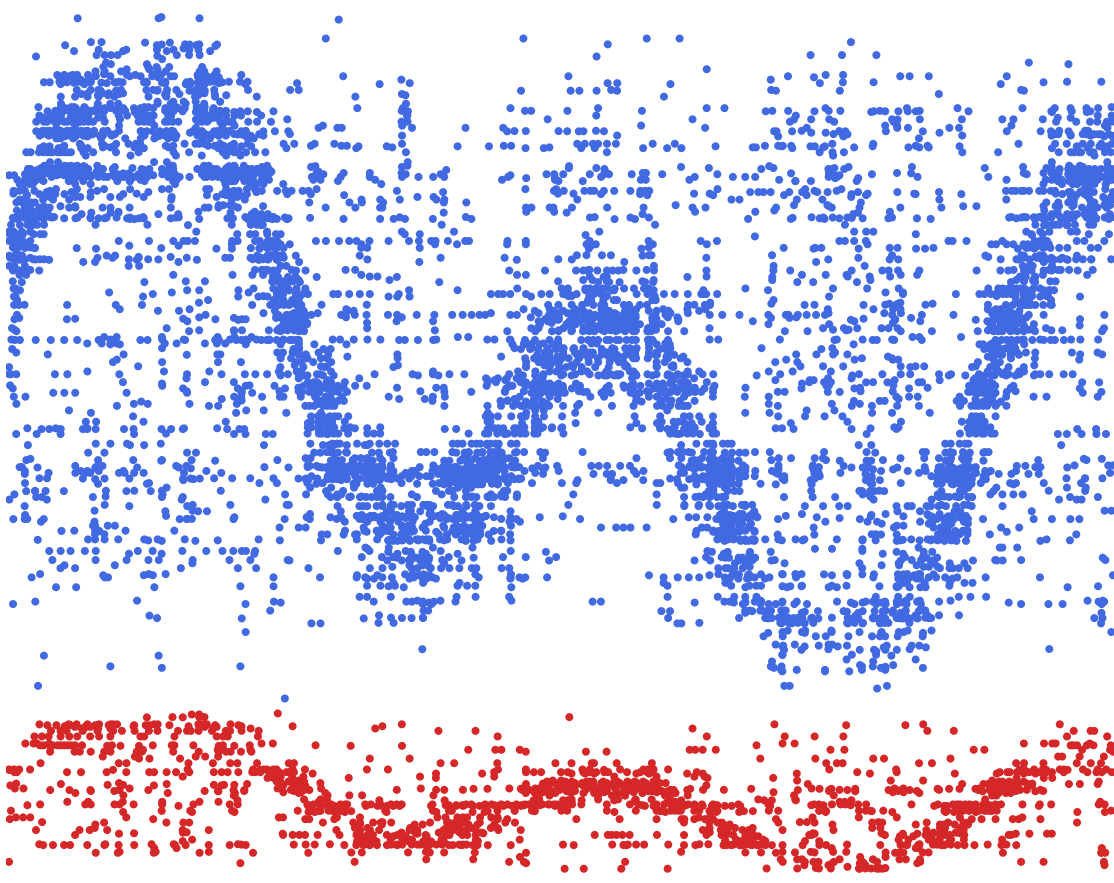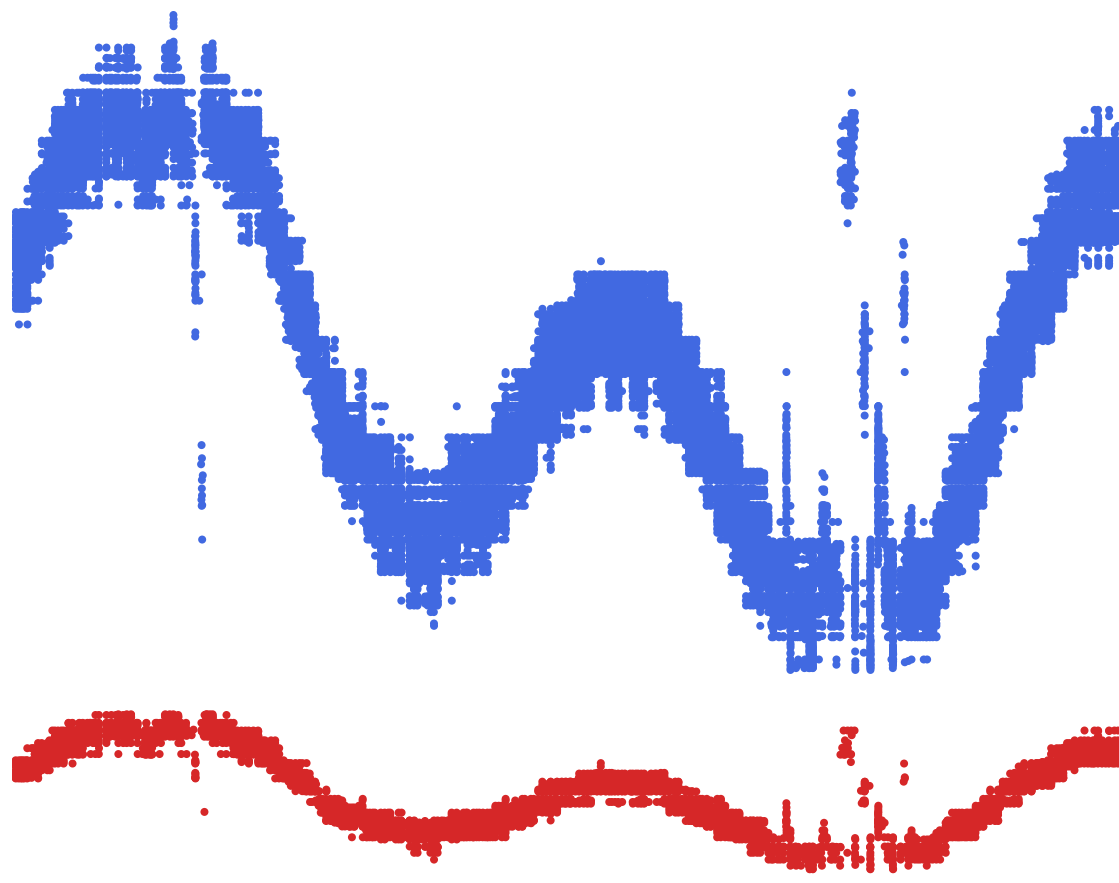

Supplement: Figure 10—source data 1. [file elife-77009-fig10-data1.zip › figure10/plots/fig10_f_raster.pdf]

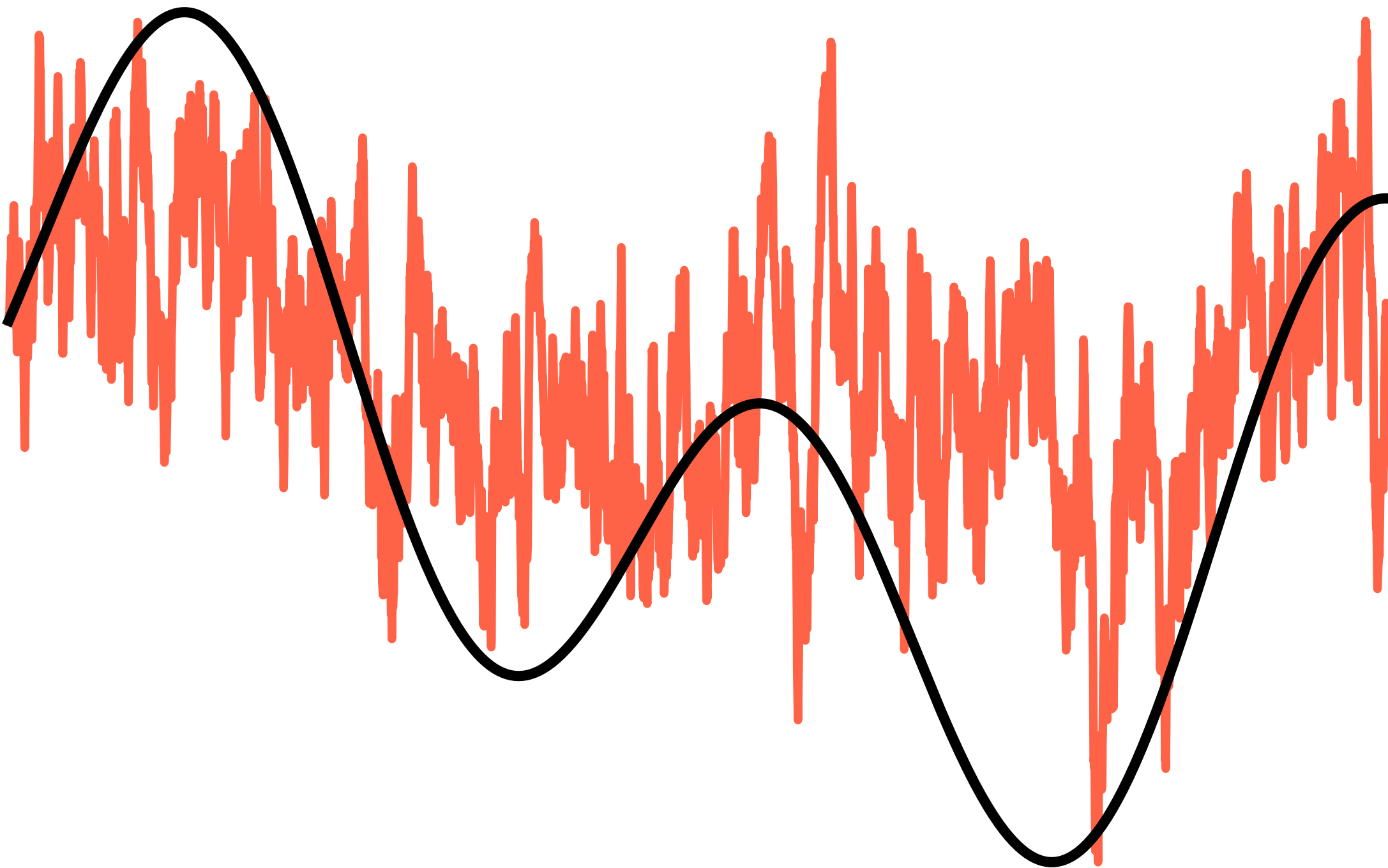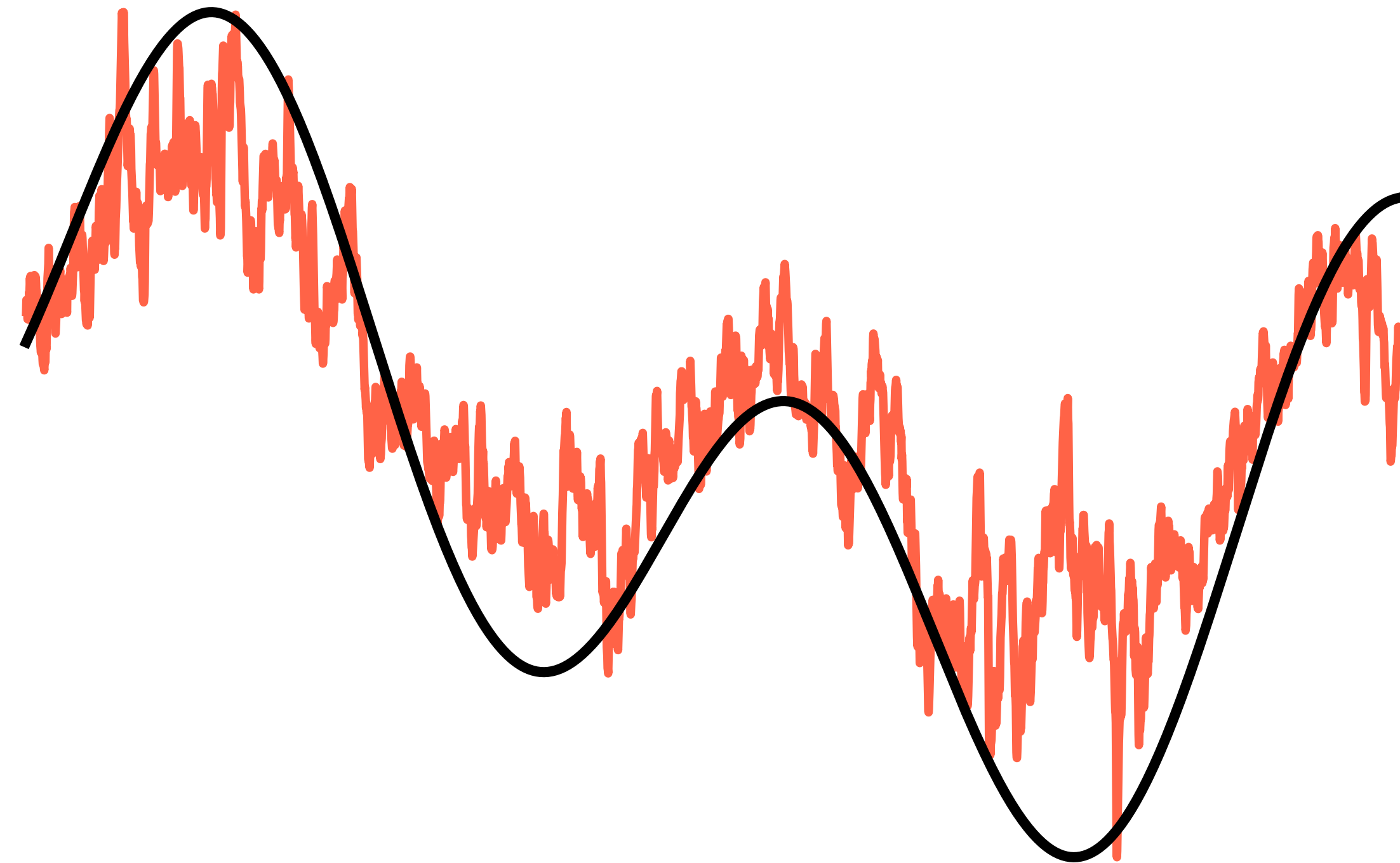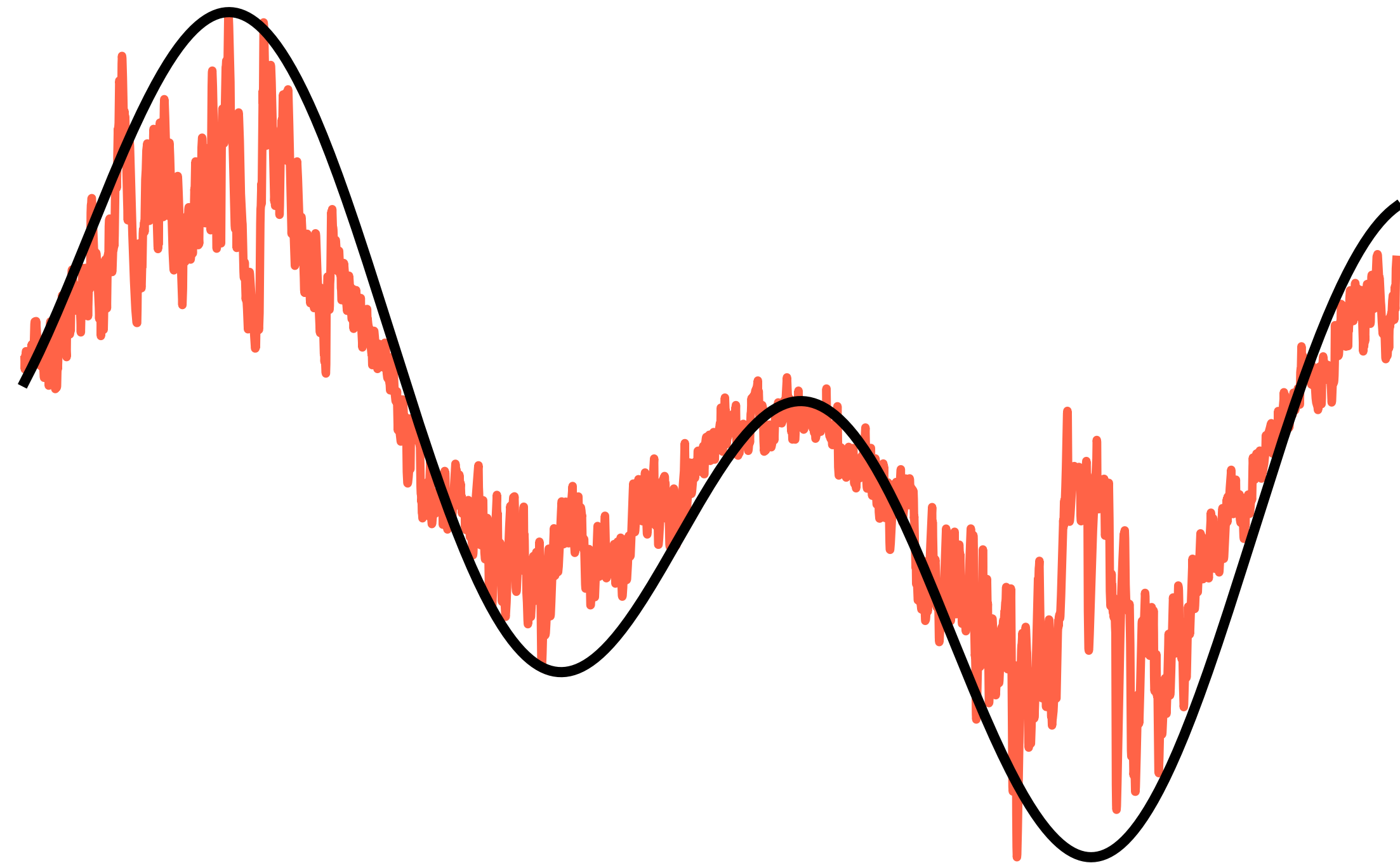

Supplement: Figure 10—source data 1. [file elife-77009-fig10-data1.zip › figure10/plots/fig10_f_readout.pdf]

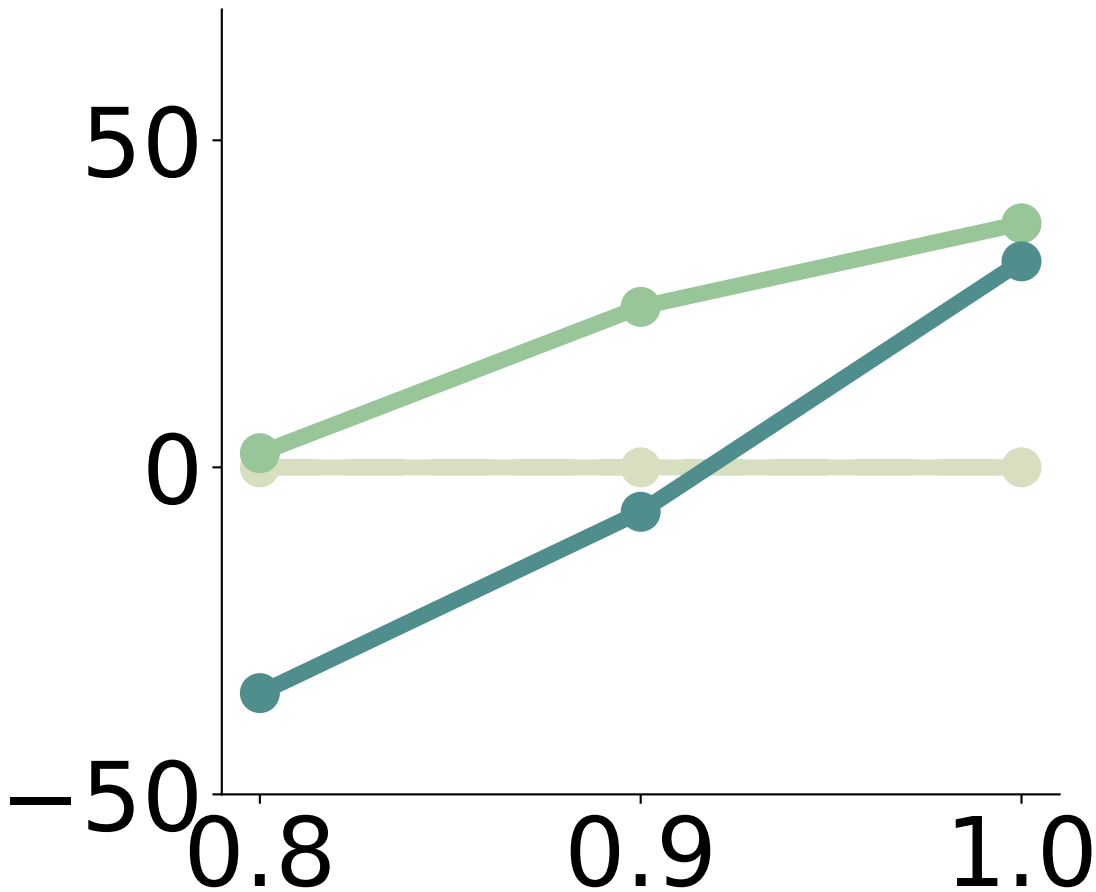

Supplement: Figure 10—source data 1. [file elife-77009-fig10-data1.zip › figure10/plots/fig10_g_perf_modules.pdf]

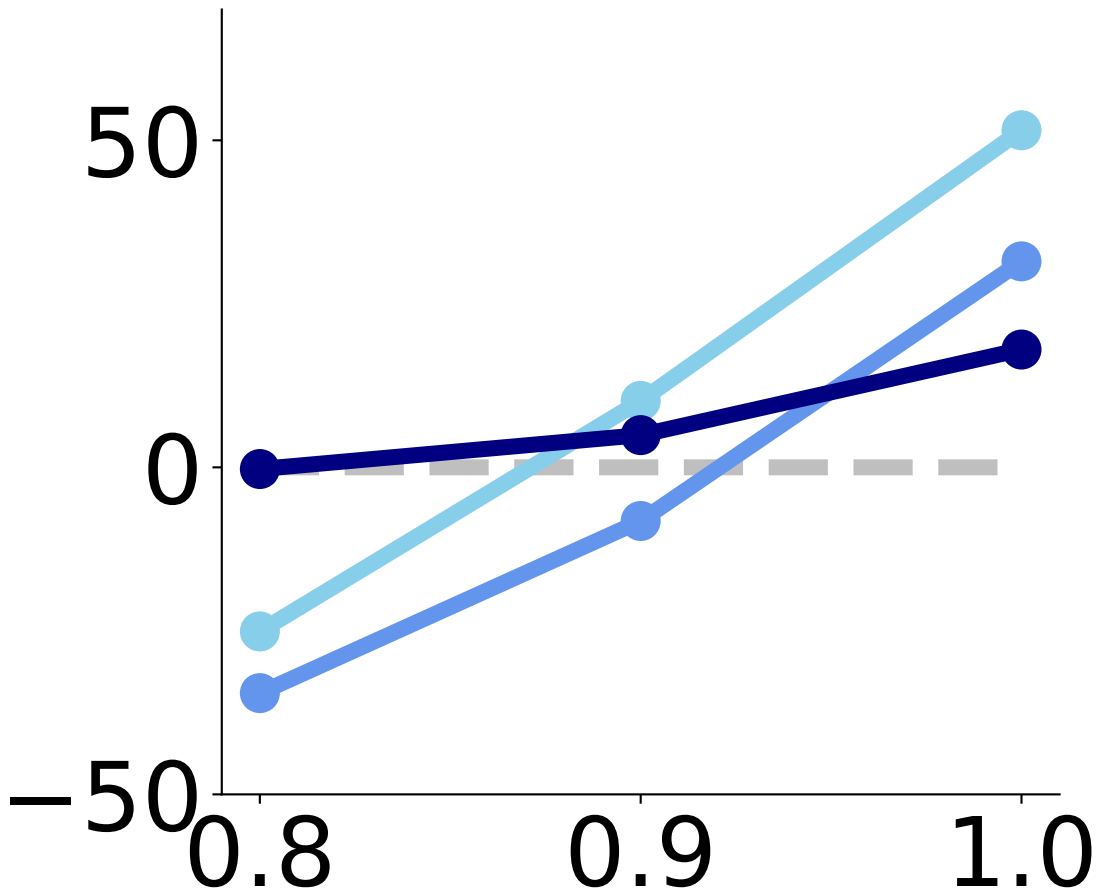

Supplement: Figure 10—source data 1. [file elife-77009-fig10-data1.zip › figure10/plots/fig10_g_perf_noise.pdf]

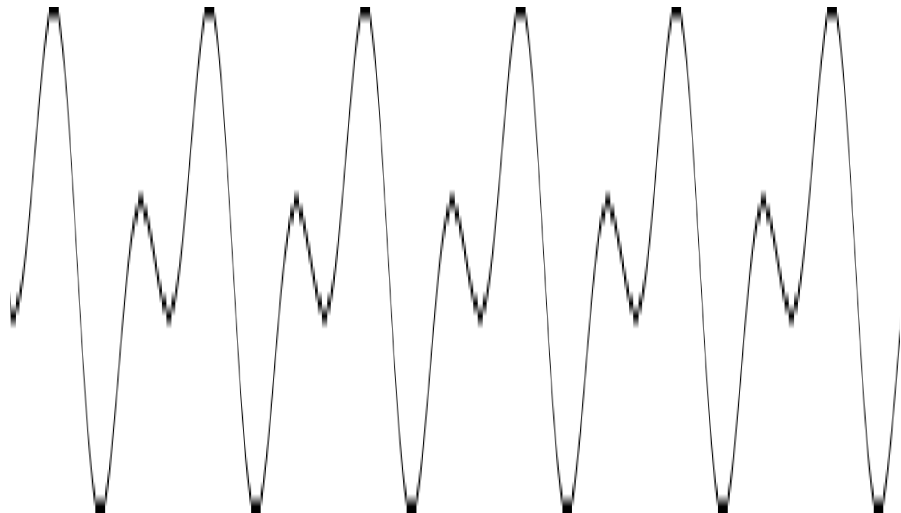

Supplement: Figure 10—source data 1. [file elife-77009-fig10-data1.zip › figure10/plots/fig10_s1_a.pdf]

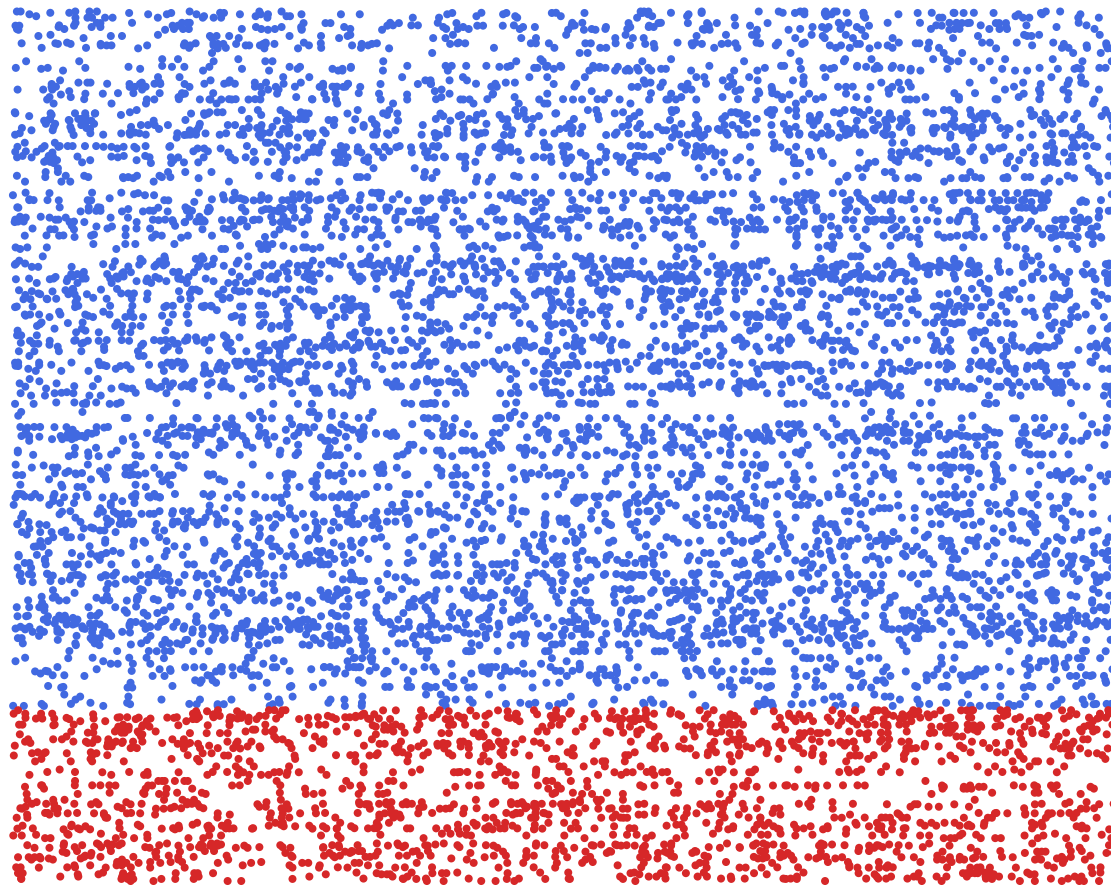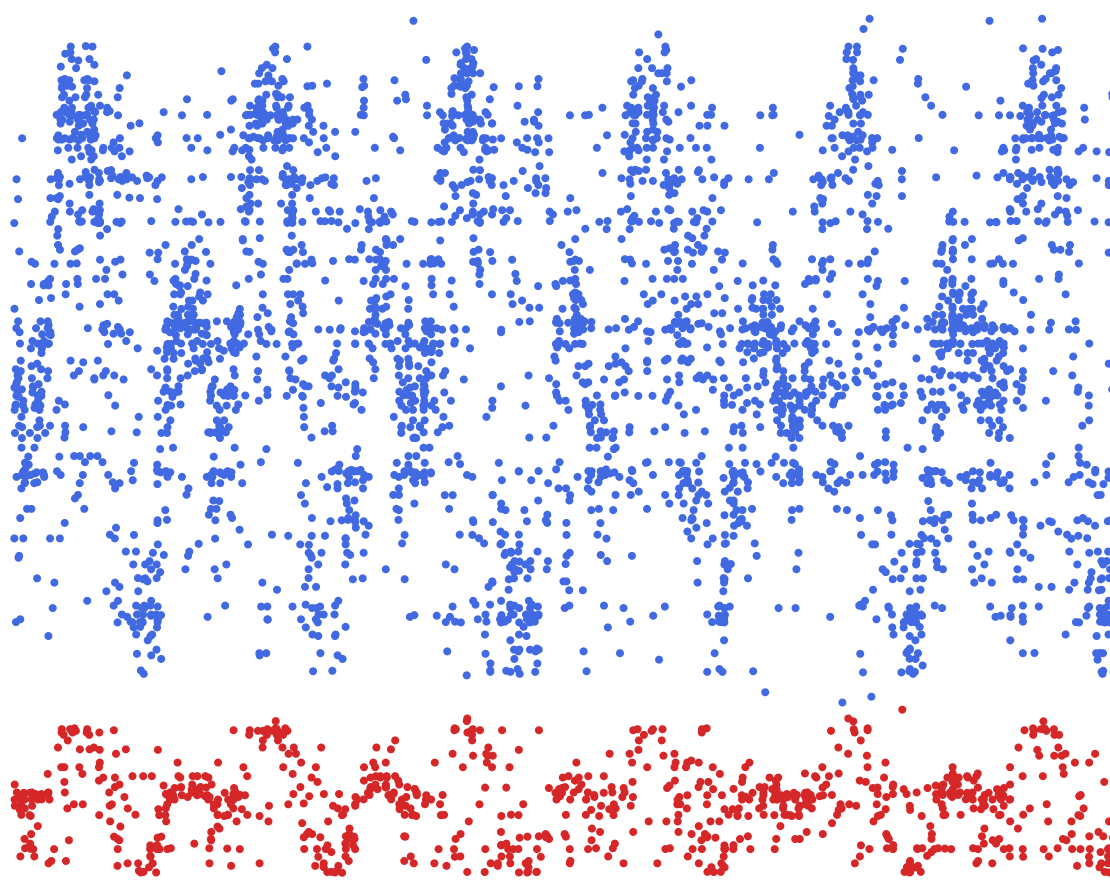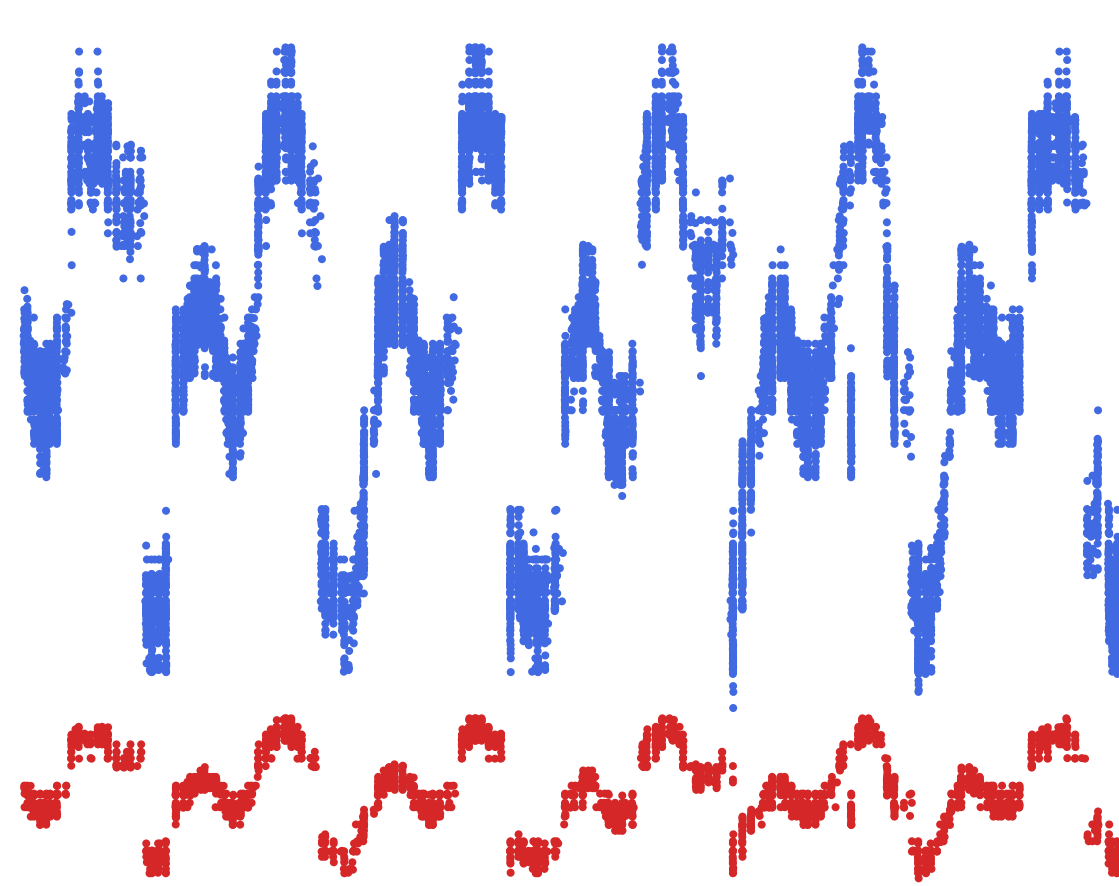

Supplement: Figure 10—source data 1. [file elife-77009-fig10-data1.zip › figure10/plots/fig10_s1_b.pdf]

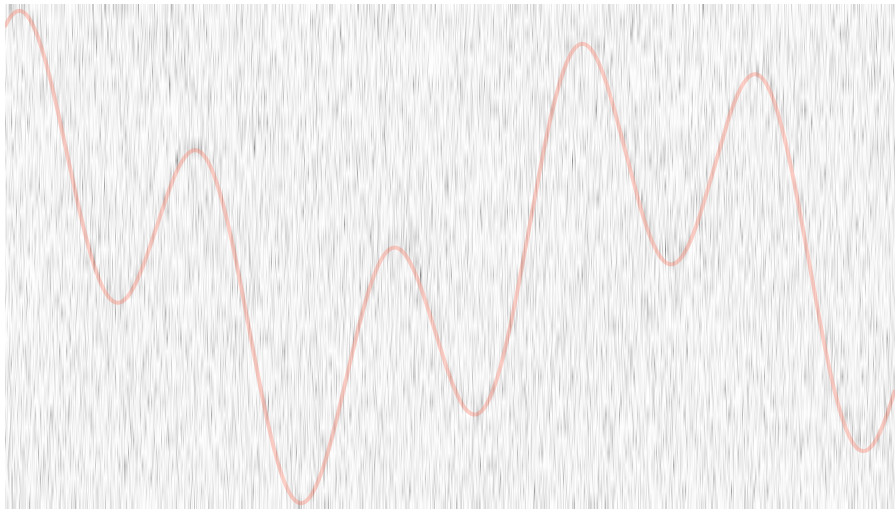

Supplement: Figure 10—source data 1. [file elife-77009-fig10-data1.zip › figure10/plots/fig10_s1_c.pdf]

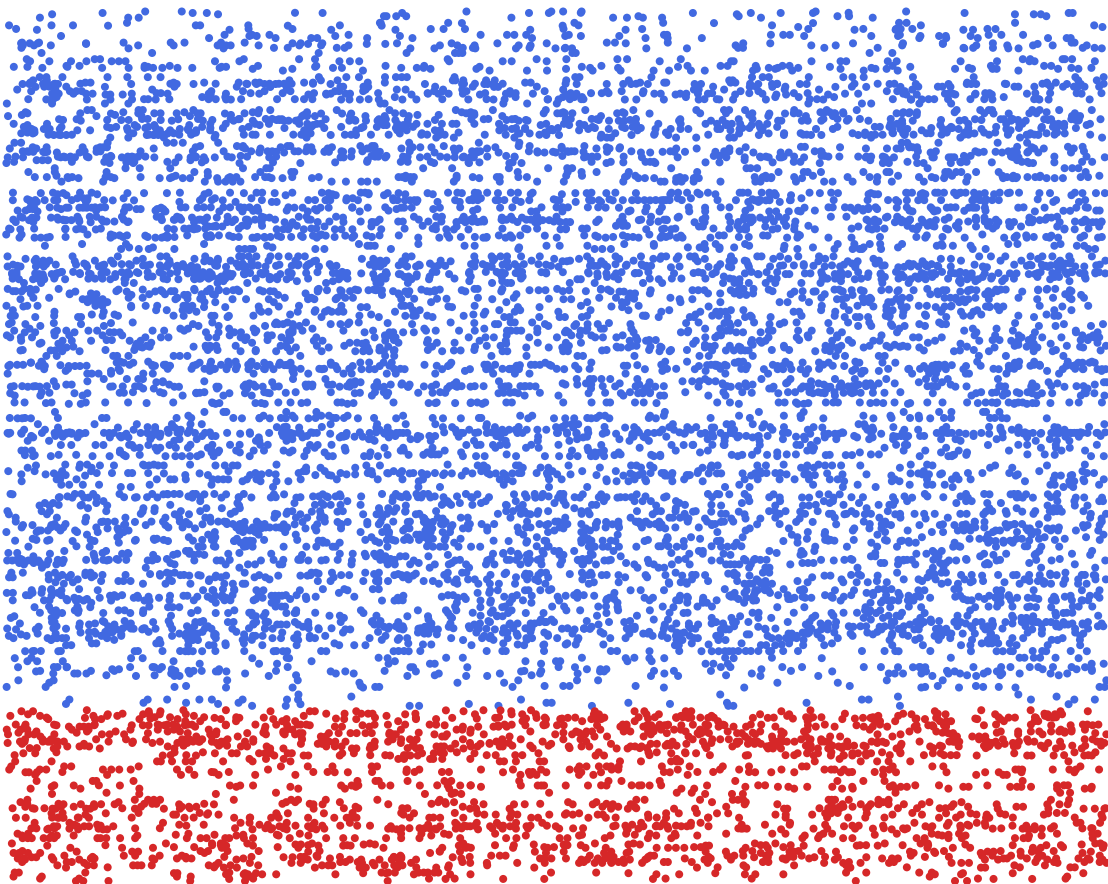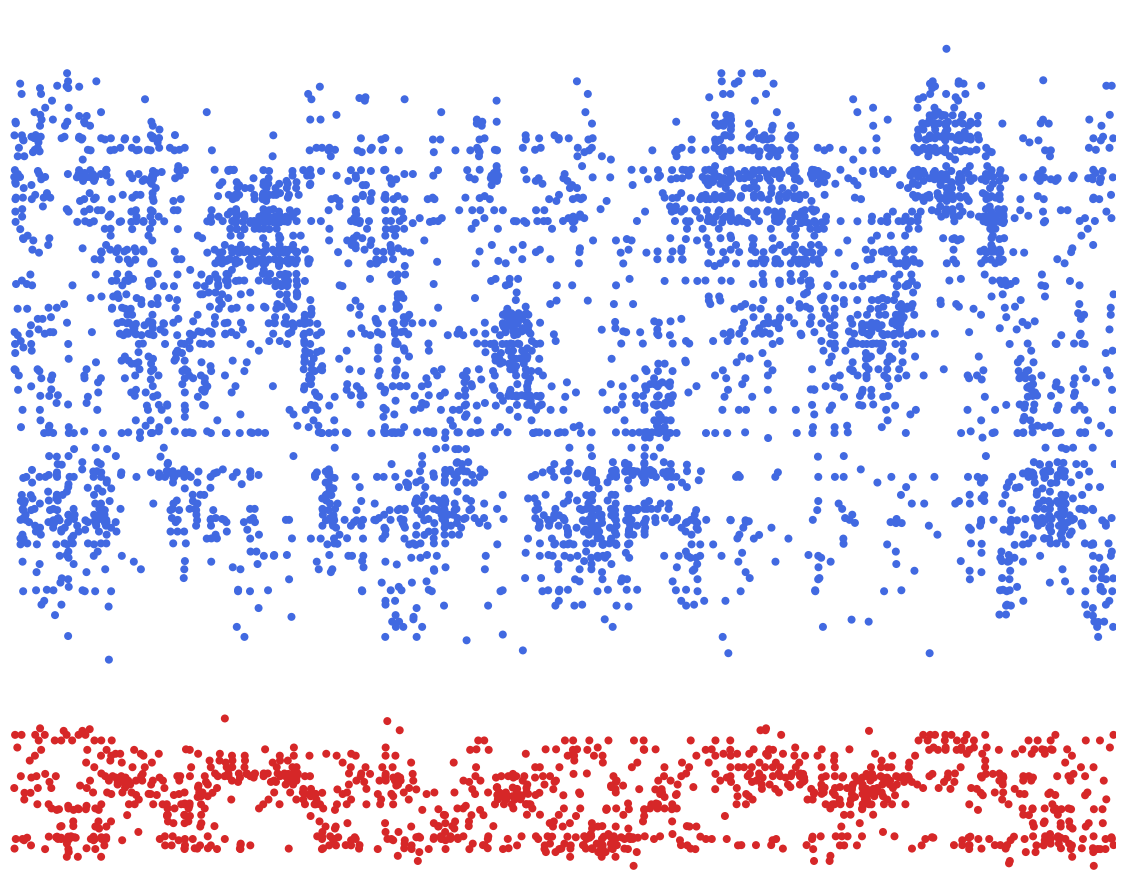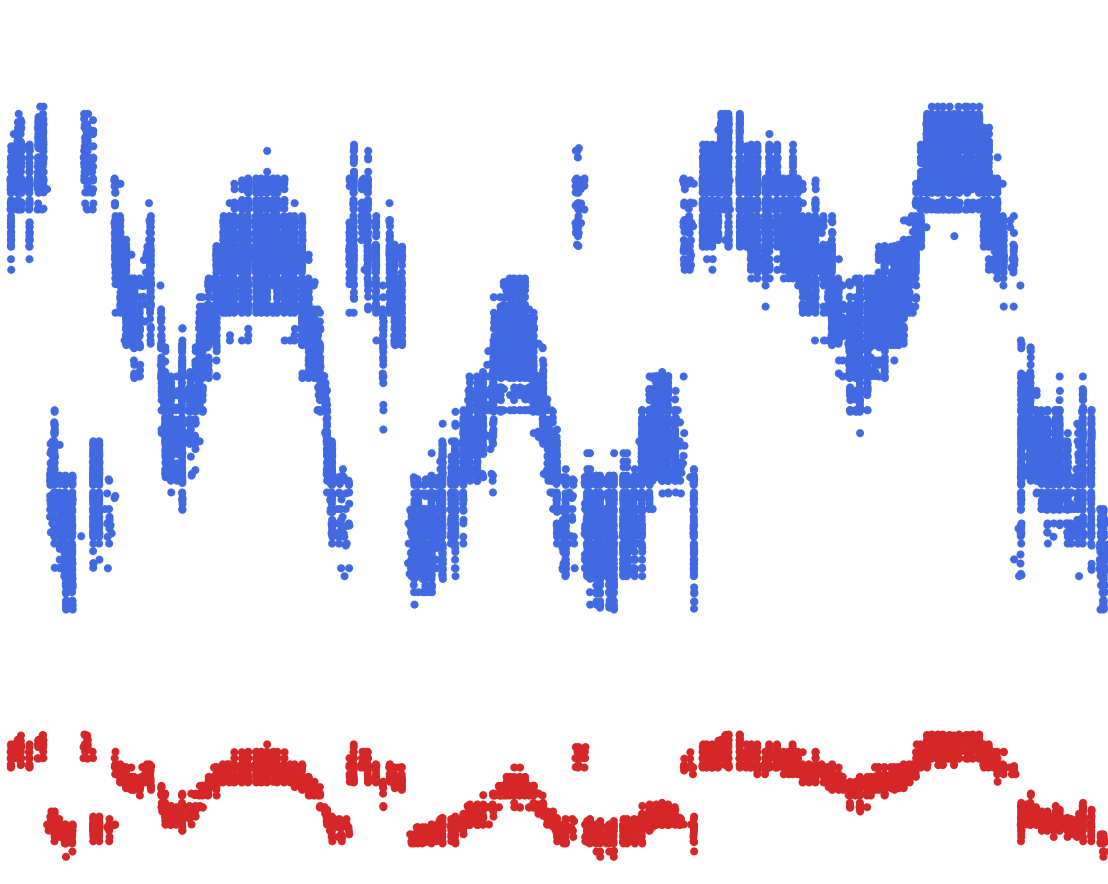

Supplement: Figure 10—source data 1. [file elife-77009-fig10-data1.zip › figure10/plots/fig10_s1_d.pdf]
