# Supplementary material for: Signal denoising through topographic modularity of neural circuits
Source: eLife. 2023 Jan 26;12:e77009. doi: 10.7554/eLife.77009 (PMC9981157; doi:10.7554/eLife.77009)

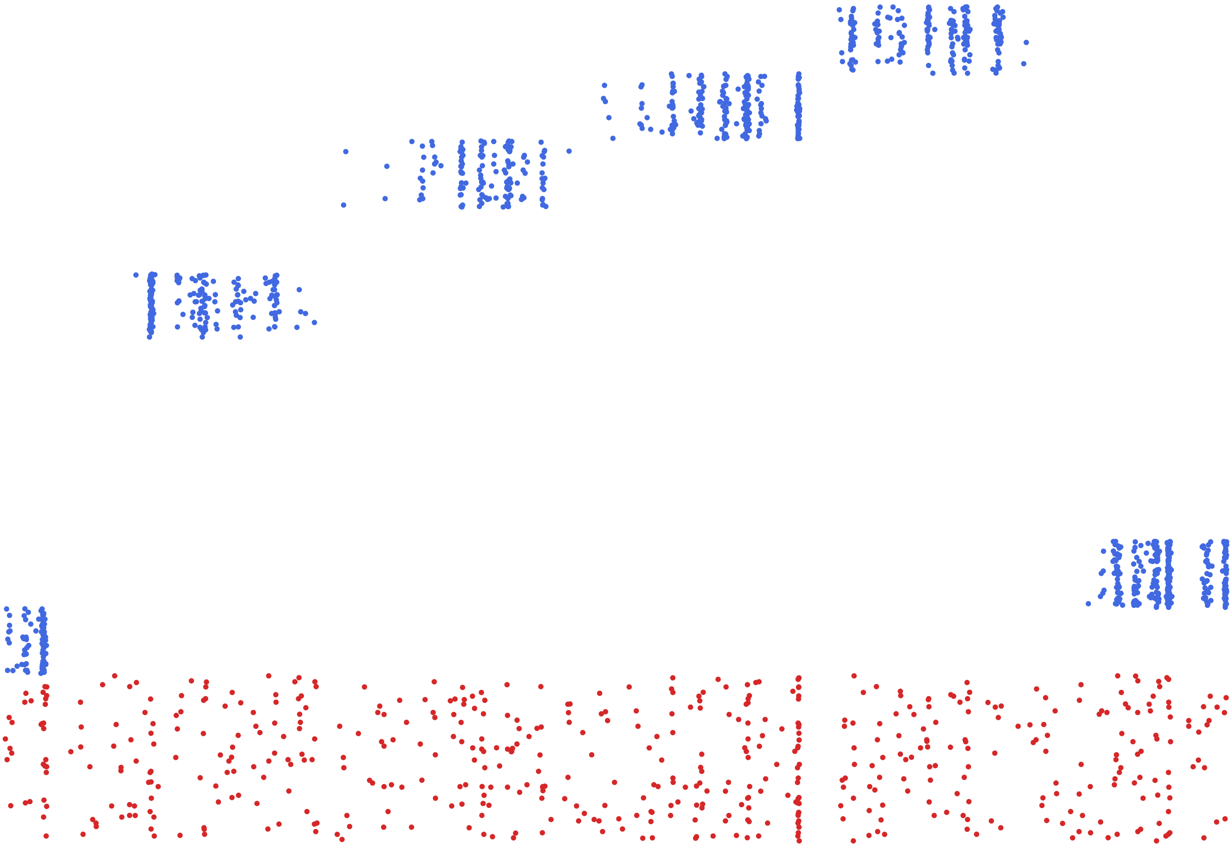

Supplement: Figure 4—source data 1. [file elife-77009-fig4-data1.zip › figure4/plots/fig4_c_right.pdf]
