# Supplementary material for: Signal denoising through topographic modularity of neural circuits
Source: eLife. 2023 Jan 26;12:e77009. doi: 10.7554/eLife.77009 (PMC9981157; doi:10.7554/eLife.77009)
Supplement: Supplementary file 1. [file elife-77009-supp1.pdf]

Supplementary File 1

**A: Model Summary**	
**Populations**	Multiple modules, each one composed of 1 excitatory and 1 inhibitory sub-population
**Topology**	None
**Connectivity**	Sparse, random recurrent connectivity with random or topographically structured feed-forward projections (fixed in-degrees)
**Neuron Model**	Leaky integrate-and-fire, fixed voltage threshold, fixed absolute refractory time, no adaptation
**Synapse Model**	Exponentially decaying postsynaptic currents, static synaptic weights, fixed delays
**Plasticity**	None
**Input**	Stochastic background spikes and inhomogeneous Poisson spikes onto $d_0 N^{\mathrm{E}}$ excitatory and $d_0 N^{\mathrm{E}}$ inhibitory neurons in SSN_0
**Measurements**	Spiking activity, membrane potentials

**B: Populations**		
**Name**	**Elements**	**Size**
E_i	`iaf_psc_exp`	8000
I_i	`iaf_psc_exp`	2000

**C: Neuron Models**	
**Name**	Leaky integrate-and-fire (LIF) neuron (`iaf_psc_exp`)
**Subthreshold Dynamics**	if $(t > t^* + \tau_{\mathrm{ref}})$<br>$\tau_{\mathrm{m}} \frac{dV_i(t)}{dt} = (V_{\mathrm{rest}} - V_i(t)) + R_{\mathrm{m}} \left(I_i^{\mathrm{E}}(t) + I_i^{\mathrm{I}}(t) + I_i^{\mathrm{X}}(t) \right)$<br>else<br>$V(t) = V_{\mathrm{reset}}$
**Spiking**	If $V(t-) < V_{\mathrm{th}}$ OR $V(t+) \geq V_{\mathrm{th}}$<br>1. set $t^* = t$ 2. emit spike with time stamp t^*

**D: Synapse Models**	
**Synaptic Transmission**	$\tau_\beta \frac{dI_i^\beta(t)}{dt} = -I_i(t) + \tau_\beta \hat{I}_\beta \sum_j \sum_k \delta(t - t_j^k)$<br>with postsynaptic potential $\mathrm{PSP}_{ij}(t) = \hat{I}_\beta \frac{R_{\mathrm{m}} \tau_\beta}{\tau_\beta - \tau_{\mathrm{m}}} \left(e^{-t/\tau_\beta} - e^{-t/\tau_{\mathrm{m}}} \right) \Theta(t)$<br>and Heaviside function $\Theta(t) = \begin{cases} 1 & t \geq 0 \\ 0 & \text{else} \end{cases}$.<br>The synaptic efficacy (weight) corresponds to the PSP amplitude:<br>$J_\beta = \hat{I}_\beta R_{\mathrm{m}} \frac{\tau_\beta}{\tau_\beta - \tau_{\mathrm{m}}} \left(\left[\frac{\tau_{\mathrm{m}}}{\tau_\beta} \right]^{\frac{-\tau_m}{\tau_m - \tau_\beta}} - \left[\frac{\tau_{\mathrm{m}}}{\tau_\beta} \right]^{\frac{-\tau_\beta}{\tau_{\mathrm{m}} - \tau_\beta}} \right)$

**E: Input**		
**Type**	**Target**	**Description**
poisson_generator	E_0, I_0	Total rate $\nu_{\mathrm{X}} \cdot \mathrm{K}_{\mathrm{X}}$
poisson_generator	E_i, I_i for $i > 0$	Total rate $0.25 \cdot \nu_{\mathrm{X}} \cdot \mathrm{K}_{\mathrm{X}}$
inhomogeneous_poisson_generator	$\mathrm{E}_0^{(k)}$, $\mathrm{I}_0^{(k)}$ for $S_k \in S$<br>$\mathrm{E}_0^{'(j)}$, $\mathrm{I}_0^{'(j)}$ for $S'_j \in S'$	Inhomogeneous Poisson process with rate ν_{stim}, changing every 200 ms

**F: Measurements**
Spiking activity, membrane potentials

Table 1: Tabular description of current-based (baseline) network model after [94].
