# Supplementary material for: Signal denoising through topographic modularity of neural circuits
Source: eLife. 2023 Jan 26;12:e77009. doi: 10.7554/eLife.77009 (PMC9981157; doi:10.7554/eLife.77009)
Supplement: Supplementary file 2. [file elife-77009-supp2.pdf]

Supplementary File 2

A: Populations		
**Name**	**Value**	**Description**
N^{E}	8000	Excitatory population size in each module
N^{I}	2000	Inhibitory population size in each module
**B: Connectivity**		
**Name**	**Value**	**Description**
ϵ	0.1	Baseline connection probability
α	0.25	Connection scaling factor for $\mathrm{SSN}_{i>0}$
p_{x}	ϵ	Connection probability for background noise input in SSN_0
	$\alpha\epsilon$	Scaled connection probability for background input in $\mathrm{SSN}_i, i > 0$
σ_{i}	$(1-\alpha) * \epsilon$	Fixed density of feed-forward projection matrices
p_{c}	$(1-m) * p_0$	Feed-forward connection probability within topographic maps
p_0	$(1-m) * p_{\mathrm{c}}$	Feed-forward connection probability between SPs on different topographic maps

B: Neuron Model		
**Name**	**Value**	**Description**
C_{m}	250 pF	Membrane capacitance
E_L	−70 mV	Resting membrane potential
τ_{m}	20 ms	Membrane time constant
V_{th}	−55 mV	Membrane potential threshold for action-potential firing
V_{reset}	−60 mV	Reset potential
τ_{ref}	2 ms	Absolute refractory period
**C: Synapse Model**		
τ_{E}	2 ms	Synaptic decay time constant for excitatory synapses
τ_{I}	2 ms	Synaptic decay time constant for inhibitory synapses
d	1.5 ms	Synaptic transmission delay
$\hat{I}_{\mathrm{E}}$	32.78 pA	Peak excitatory current
$\hat{I}_{\mathrm{I}}$	g32.78 pA	Peak inhibitory current
J_{E}	0.2 mV	EPSP amplitude
J_{I}	g0.2 mV	IPSP amplitude
g	−12	Scaling factor for the inhibitory synapses

Table 2: Summary of all the model parameters for the current-based network. For more details, see [15].
