# Supplementary material for: Signal denoising through topographic modularity of neural circuits
Source: eLife. 2023 Jan 26;12:e77009. doi: 10.7554/eLife.77009 (PMC9981157; doi:10.7554/eLife.77009)
Supplement: Supplementary file 3. [file elife-77009-supp3.pdf]

Supplementary File 3

**A: Populations**		
**Name**	**Value**	**Description**
N^{E}	8000	Excitatory population size in each module
N^{I}	2000	Excitatory population size in each module
**B: Connectivity**		
**Name**	**Value**	**Description**
d	1.5 ms	Synaptic transmission delay
$\overline{g}_{\mathrm{E}}$	1nS	Excitatory synaptic conductance
$\overline{g}_{\mathrm{I}}$	$g\overline{g}_{\mathrm{E}}$nS	Inhibitory synaptic conductance
g	-16	Scaling factor for the inhibitory synapses
ϵ	0.1	Baseline connection probability
α	0.25	Connection scaling factor for $\mathrm{SSN}_{i>0}$
p_{x}	ϵ	Connection probability for background noise input in SSN_0
	$\alpha\epsilon$	Scaled connection probability for background input in $\mathrm{SSN}_i, i > 0$
σ_{i}	$(1-\alpha) * \epsilon$	Fixed density of feed-forward projection matrices
p_{c}	$(1-m) * p_0$	Feed-forward connection probability within topographic maps
p_0	$(1-m) * p_{\mathrm{c}}$	Feed-forward connection probability between SPs on different topographic maps
**B: Neuron Model**		
**Name**	**Value**	**Description**
C_{m}	250 pF	Membrane capacitance
E_L	-70 mV	Resting membrane potential
τ_{m}	15 ms	Membrane time constant
V_{th}	-50 mV	Membrane potential threshold for action-potential firing
V_{reset}	-60 mV	Reset potential
τ_{ref}	2 ms	Absolute refractory period
g_{L}	16.7nS	Leak conductance
**C: Synapse Model**		
τ_{E}	5 ms	Synaptic decay time constant for excitatory synapses
τ_{I}	10 ms	Synaptic decay time constant for inhibitory synapses
V_{E}	0 mV	Excitatory reversal potential
V_{I}	-80 mV	Inhibitory reversal potential

Table 3: Parameter values for the conductance-based model.
