# Supplementary material for: Signal denoising through topographic modularity of neural circuits
Source: eLife. 2023 Jan 26;12:e77009. doi: 10.7554/eLife.77009 (PMC9981157; doi:10.7554/eLife.77009)
Supplement: Supplementary file 4. [file elife-77009-supp4.pdf]

Supplementary File 4

A: Model Summary	
**Populations**	Multiple modules, each one composed of 1 excitatory and 1 inhibitory sub-population
**Topology**	None
**Connectivity**	Sparse, random recurrent connectivity with modular topographic feed-forward projections (fixed in-degrees)
**Neuron Model**	Rate neuron with shifted tanh gain function
**Synapse Model**	Delayed rate connection
**Plasticity**	None
**Input**	Uniformly distributed input onto $d_0 N^{\mathrm{E}}$ excitatory and $d_0 N^{\mathrm{E}}$ inhibitory neurons in SSN_0
**Measurements**	Unit output (rate)

B: Populations		
**Name**	**Elements**	**Size**
E_i	rate neuron	2400
I_i	rate neuron	600

C: Neuron Models	
**Name**	Rate neuron
**Differential equation**	$\boldsymbol{\tau_x} \frac{\mathrm{d}\boldsymbol{x}}{\mathrm{dt}} = -\boldsymbol{x} + W\boldsymbol{r} + W^{\mathrm{in}}\boldsymbol{u} - \boldsymbol{b}^{\mathrm{rec}} + \sqrt{2\boldsymbol{\tau_x}}\sigma_{\mathrm{X}}\xi$ $\boldsymbol{r} = 0.5(1 + \tanh(\boldsymbol{x}))$

D: Input		
**Type**	**Target**	**Description**
random uniform distribution	E_0, I_0	Step signal input to SSN_0, changing every 200 ms, with amplitude 0.8
Gaussian white noise	E_i, I_i for $i \in \{0..5\}$	Intrinsic unit noise

E: Measurements
Unit output (rate)

Table 4: Description of the rate model.
