# Supplementary material for: Signal denoising through topographic modularity of neural circuits
Source: eLife. 2023 Jan 26;12:e77009. doi: 10.7554/eLife.77009 (PMC9981157; doi:10.7554/eLife.77009)
Supplement: Supplementary file 5. [file elife-77009-supp5.pdf]

Supplementary File 5

A: Populations		
**Name**	**Value**	**Description**
N^{E}	2400	Number of excitatory units in each module
N^{I}	600	Number of inhibitory units in each module
**B: Connectivity**		
**Name**	**Value**	**Description**
d	1 ms	Synaptic transmission delay
ϵ	0.2	Baseline connection probability
w_{in}	$\sim U(0.9, 1.0)$	Input weights
w_{in}	$\sim N_{\mathrm{tr}}(0, 1/\sqrt{\epsilon N}) > 0$	Recurrent and feed-forward weights drawn from a normal distribution truncated to positive values
g	-6	Scaling factor for the inhibitory synapses
**B: Neuron Model**		
**Name**	**Value**	**Description**
τ	10 ms	Unit time constant
b	1	Bias term
σ_{X}	1.5	Scaling term for unit noise

Table 5: Rate model parameters.
